# GNPS2 molecular networking reveals metabolic diversity in fungi isolated from protease-rich fruits

**DOI:** 10.1007/s13659-026-00615-1

**Published:** 2026-04-07

**Authors:** Vitor de Souza Mazucato, Winner Duque Rodrigues, Ludmilla Tonani, Marcia Regina von Zeska Kress, Paulo Cezar Vieira

**Affiliations:** 1 Department of BioMolecular Sciences, Ribeirão Preto School of Pharmaceutical Sciences, University of São Paulo, Ribeirão Preto, SP Brazil; 2 Department of Clinical Analysis, Toxicology, and Food Science, Ribeirão Preto School of Pharmaceutical Sciences, University of São Paulo, Ribeirão Preto, SP Brazil

**Keywords:** *Fusarium*, Metabolomics, GNPS2, Natural product, Phytopathogenic

## Abstract

Fungi are a rich source of chemical diversity for bioprospecting, yet most of their metabolic potential remains unexplored. This study investigated the metabolic profile of phytopathogenic fungi of the genus *Fusarium*, isolated from fruits with high protease content, such as papaya and pineapple. Comparative metabolomics was used to evaluate how culture substrates and co-cultivation with the endophyte *Neofusicoccum ribis* modulate secondary metabolite production. Molecular Networking analysis (GNPS2) revealed an impressive diversity of 173 annotated metabolites, including one previously undescribed compound. Metabolite production was highly dependent on the substrate and fungal strain, showing a strong correlation between phylogeny and metabolic profiles. Several extracts displayed significant inhibitory activity against the protease papain, with some co-cultivation combinations further enhancing this effect. These findings highlight how environmental conditions and microbial interactions activate silent biosynthetic pathways, confirming these fungi as promising sources for bioactive molecule discovery.

**Graphical Abstract:**

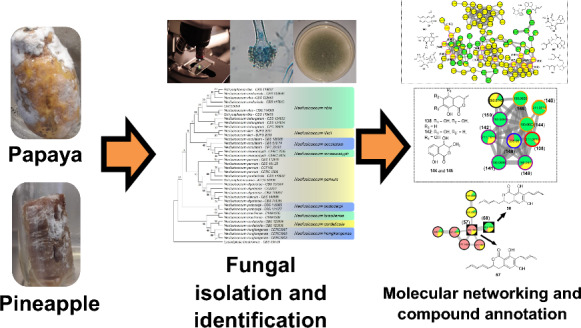

**Supplementary Information:**

The online version contains supplementary material available at 10.1007/s13659-026-00615-1.

## Introduction

Fungi are versatile organisms and primary sources of bioactive natural products, with significant applications in drug discovery, especially for antibiotics and antifungals [1]. It is estimated that approximately 42% of microbial bioactive molecules are of fungal origin, reflecting their remarkable chemical diversity. Yet, their chemical potential remains largely untapped. While up to 13.2 million fungal species are estimated to exist, only about 150,000 have been described, and less than 7% of these have had their secondary metabolites investigated [2, 3]. Therefore, isolating fungi directly from nature is fundamental to access organisms with unique chemical profiles adapted to specific ecological niches. Obtaining fungal strains from sources such as host fruits enables the discovery of metabolites with previously unknown biological activities, often activated only under specific environmental conditions or in ecological contexts of competition [3].

In the present study, twelve fungal strains were isolated from fruits with high protease content and activity, with particular emphasis on five representatives of the genus *Fusarium*. These strains were selected not only for their remarkable ecological adaptability but also for their importance as phytopathogens, known to cause significant economic losses in agriculture [4–6]. In Brazil, *Fusarium fujikuroi*, *F. guttiforme*, and *F. graminearum* infect major crops like papaya, pineapple, and corn, causing up to 40% losses in fruit production [7–9]. Thus, elucidating the chemistry of the metabolites produced by these fungi may contribute both to improving disease management and to the prospection of new bioactive molecules.

Proteases play central roles in diverse biological processes, contributing both to metabolic regulation and to the defense of organisms against invasive microorganisms. In the biomedical field, their importance is underscored by the development of protease inhibitors as therapeutic strategies against severe diseases, including cancer and viral infections. A notable example is COVID-19, in which SARS-CoV-2 relies on its proteases, such as Mpro and PLpro, for replication, making these enzymes strategic targets for antiviral development [10–15].

In plants, proteases play a crucial role in plant-pathogen interactions. Fruits like papaya and pineapple contain high levels of proteases such as papain and bromelain, which defend the plant by degrading microbial proteins and inhibiting fungal invasion [16–19]. Nevertheless, despite this biochemical barrier, these fruits remain susceptible to pathogen colonization, suggesting that fungi have developed efficient resistance mechanisms. A well-documented example is *Fusarium proliferatum* in pineapples, which produces inhibitors capable of blocking plant proteases such as papain, thereby promoting infection and revealing a co-evolutionary process between host and pathogen [20, 21]. In this context, fungal extracts were assessed for their ability to inhibit papain, our model protease.

However, despite their recognized biotechnological potential, the metabolic profile of *Fusarium* isolates from tropical fruits and their response to specific environmental conditions remains poorly explored. In particular, it is unknown how the culture substrate—which mimics different nutritional sources from the natural environment—and microbial interactions influence the activation of their biosynthetic pathways and the production of bioactive molecules, such as protease inhibitors. To investigate the metabolic potential of these strains, the *Fusarium* isolates were cultivated in four distinct media prepared from rice, wheat, corn, and sugarcane bagasse (SCB).Varying the substrate aimed to mimic different environmental conditions, thereby stimulating diverse biosynthetic pathways, including silent ones [22–25]. Additionally, six *Fusarium* strains were co-cultured with the papaya endophyte *Neofusicoccum ribis* on rice medium. Co-culture strategies have proven effective in triggering the expression of otherwise inactive metabolic pathways, promoting the production of unique compounds that emerge from microbial species interactions [26].

To characterize the metabolites produced under different conditions, we applied comparative metabolomics approaches using the GNPS platform. This approach, combined with co-cultivation strategies, aims precisely to address this gap, unveiling the hidden chemical diversity of these fungi and their capacity to produce metabolites with relevant bioactivity under stimulated conditions.

## Results and discussion

### Fungal isolation

The methodology employed for isolating endophytic and phytopathogenic fungi proved effective for obtaining new environmental fungal strains for the laboratory collection. The isolates were initially evaluated based on macroscopic colony characteristics, allowing grouping according to macromorphological similarities. One representative strain from each group was selected for further analyses (Fig. S1). These strains were assigned the code LMC2300 and subjected to micromorphological examination (Fig. S2). Distinctive conidiogenous structures enabled preliminary characterization, confirming the presence of five *Fusarium* species and two *Aspergillus* species [27, 28]. For strain LMC23009, however, it was not possible to photograph the reproductive structures due to the specific developmental pattern of this species.

For precise identification, phylogenetic trees were constructed for each isolate using reference loci obtained from the literature [27–35] (Tables S1-8). The dataset included fungal species names, strain codes, and corresponding GenBank accession numbers for each locus. Distinct fungal strains, along with an outgroup, were incorporated in each phylogenetic reconstruction. Maximum Likelihood (ML) analysis enabled species-level resolution, with fungal strain codes, host fruit, and isolation conditions detailed in Table 1.

**Table 1:** Molecular identification of fungal isolates from papaya and pineapple, with corresponding GenBank accession numbers

Isolate code	Fungal species	Host fruit	Ecological role	Target gene/Region	GenBank accession number
LMC23006	N. ribis	Papaya	Endophytic	ITS; TUB2; RPB2	PV719604; PV727023; PV727017
LMC23007.1	*Gilbertella persicaria*	Papaya	Phytopathogenic	*ITS*	PX287134
LMC23007.2	*Fusarium falciforme*	Papaya	Phytopathogenic	*RPB2*	PV727018
LMC23008	*Fusarium petroliphilum*	Papaya	Phytopathogenic	*RPB2*	PV727019
LMC23009	*Mucor circinelloides*	Papaya	Endophytic	*ITS*	PX314507
LMC23011	*Aspergillus flavus*	Papaya	Phytopathogenic	*cmd*	PX361046
LMC23012	Fusarium sacchari	Papaya	Phytopathogenic	RPB2	PV727020
LMC23014	*Talaromyces funiculosus*	Pineapple	Endophytic	*ITS*	PX287135
LMC23015	*F. sacchari*	Pineapple	Phytopathogenic	*RPB2*	PV727021
LMC23017	*Trichoderma* sp*.*	Pineapple	Endophytic	*-*	-
LMC23018	*Fusarium verticillioides*	Pineapple	Phytopathogenic	*RPB2*	PV727022
LMC23020	*Aspergillus terreus*	Pineapple	Phytopathogenic	*ITS*	PV089058

*ITS* internal transcribed spacer (*ITS*) region of ribosomal DNA, *TUB2* tubulin, *RPB2* second largest subunit of the DNA-dependent RNA polymerase II, *cmd* calmodulin

One isolate, LMS23017 (*Trichoderma* sp.), could not be resolved to the species level, indicating the need for additional loci to achieve accurate classification. Notably, a higher proportion of phytopathogenic fungi was recovered compared to endophytic strains, suggesting the predominance of opportunistic environmental fungi that proliferate under favorable conditions.

The predominance of *Fusarium* species among the isolated fungi was particularly notable, with five out of the twelve obtained fungal specimens belonging to this genus. This finding underscores the ecological prevalence of *Fusarium*, a fungus renowned for its ability to persist in soil for extended periods while awaiting favorable conditions for development and proliferation. Such ecological resilience is attributed mainly to its capacity to produce resistant conidia that remain viable until environmental factors such as humidity, temperature, and the availability of suitable hosts trigger their germination and subsequent growth [4, 6, 36].

**Fig. 1:**
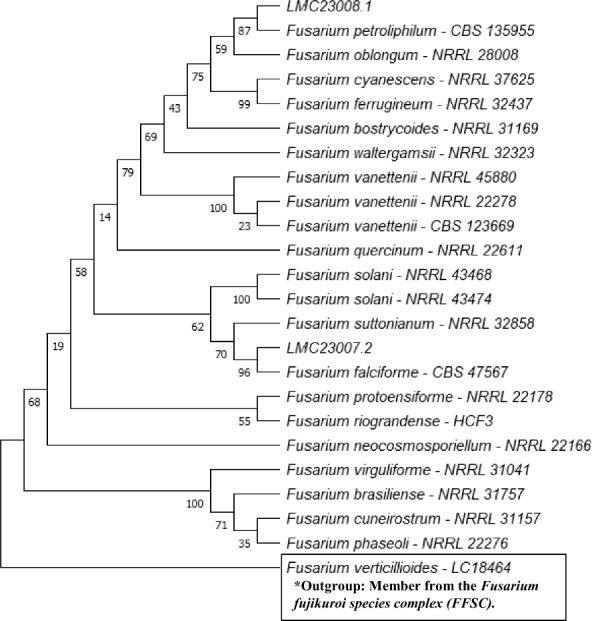
Phylogenetic tree of the *Fusarium solani* species complex (FSSC). Maximum Likelihood phylogenetic tree based on *RPB2* sequences showing the placement of isolates LMC23007.2 (*Fusarium falciforme*) and LMC23008 (*Fusarium petroliphilum*) within the *Fusarium solani* species complex. Bootstrap support values (1000 replicates) are indicated at the nodes. Reference sequences were retrieved from GenBank, with species names and corresponding strain codes provided

**Fig. 2:**
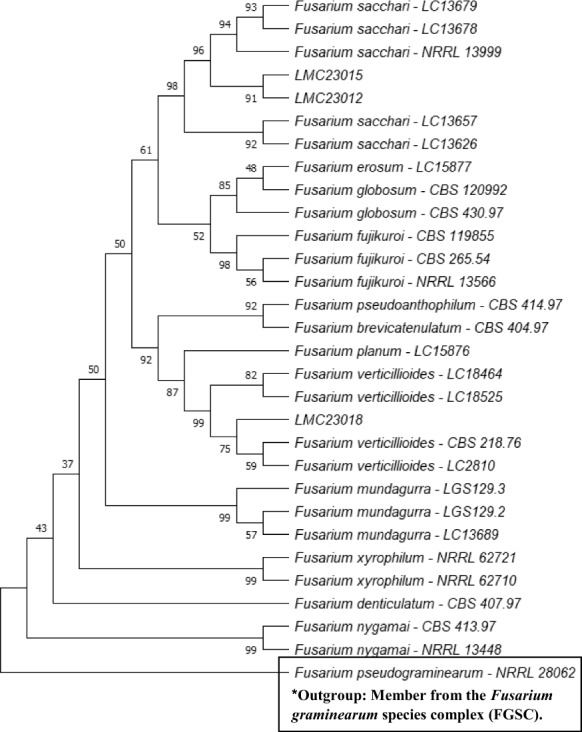
Phylogenetic tree of the *Fusarium fujikuroi* species complex (FFSC). Maximum Likelihood phylogenetic tree based on *RPB2* sequences showing the placement of isolates LMC23012 (*Fusarium sacchari*), LMC23015 (*F. sacchari*), and LMC23018 (*Fusarium verticillioides*) within the *Fusarium fujikuroi* species complex. Bootstrap support values (1000 replicates) are indicated at the nodes. Reference sequences were retrieved from GenBank, with species names and corresponding strain codes provided

For precise taxonomic identification, two phylogenetic trees were constructed: one for the *Fusarium solani* species complex (Fig. 1), which enabled the identification of strains LMC23007.2 and LMC23008; and another for the *Fusarium fujikuroi* species complex (Fig. 2), which facilitated the classification of strains LMC23012, LMC23015, and LMC23018. This phylogenetic approach provided robust species-level resolution for the *Fusarium* isolates, confirming their placement within these well-characterized species complexes.

In Fig. 2, the close phylogenetic relationship between the *Fusarium sacchari* strains LMC23012 and LMC23015 is evident, despite their isolation from different host fruits. Strain LMC23012 exhibited a macroscopically white mycelium with the production of purple pigments, whereas strain LMC23015 produced yellow-pigmented mycelia, as depicted in Fig. S1. In addition, the laboratory collection included *F. guttiforme* (F1-MMBF- 04/07), a member of the *F. fujikuroi* species complex, which was cultivated under the same experimental conditions as the five isolated *Fusarium* species.

**Fig. 3:**
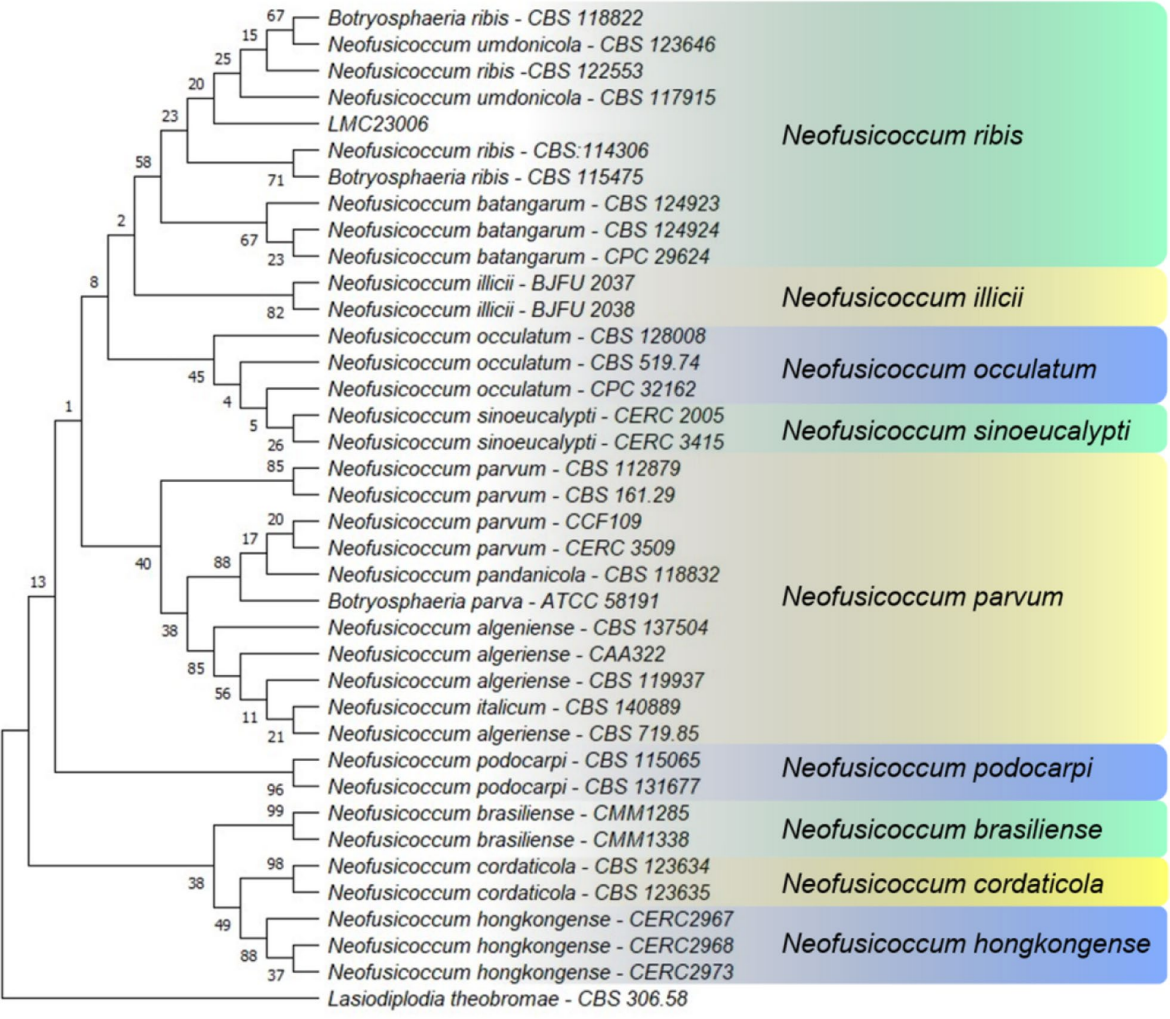
Phylogenetic tree of the LMC23006 fungus. Maximum Likelihood phylogenetic tree based on *ITS*, *TUB2*, and *RPB2* sequences confirming the identity of isolate LMC23006 as *Neofusicoccum ribis*. Bootstrap support values (1000 replicates) are indicated at the nodes. The tree encompasses representatives of several *Neofusicoccum* species complexes and reveals synonymous species within the genus, such as *N. parvum*

The identity of isolate LMC23006 (*N. ribis*) was confirmed through phylogenetic analysis (Fig. 3). The phylogenetic tree includes representatives of different species complexes within the genus *Neofusicoccum*, highlighting synonymous designations (*e.g.*, *N. parvum*) and taxonomic relationships relevant to the classification of *N. ribis*.

For the remaining isolates, phylogenetic trees were also constructed to confirm their identification, including *Aspergillus flavus*, *Aspergillus terreus*, *Mucor circinelloides*, *Talaromyces funiculosus*, and *Trichoderma* sp. These analyses are provided in Figs. S3–S7.

### Molecular networking and isolated compounds

The molecular networking for each *Fusarium* species was constructed using the GNPS2 platform to study the metabolites produced under different culture conditions, and the GNPS workflow link for each analysis is provided in Table S9 with its respective conditions. The *Fusarium* strains were co-cultivated with the endophytic fungus *N. ribis* to promote fungal interaction and competition, since all isolates originated from fruit samples. This strategy aimed to investigate their ecological relationships and potentially activate silent biosynthetic pathways. Annotation was performed based on spectral similarity to the GNPS2 fragmentation database, and all matches and predictions were verified using.

ChemWalker and/or SIRIUS v. 6.2.2. Additionally, compounds annotated by these tools were cross-referenced with literature to confirm their natural origin. For each fungus, a molecular networking Fig., annotated compounds, and a corresponding retention time-ordered compound table were generated. Isolation of selected compounds further enhanced the reliability of the results. The compounds were analyzed and annotated using molecular networking. Some extracts were fractionated, and for the discussion, the molecular networks were divided by fungal species to examine the influence of the culture medium on metabolic production and chemical diversity. Finally, the molecular network of the co-cultures of the six *Fusarium* strains with *N. ribis* was analyzed. All NMR data for the isolated compounds are presented in the experimental section, together with literature references. The data for common, well-documented metabolites are provided there as well. In contrast, spectra were only included in the Supplementary Material for the new compound and for those exhibiting structures that differed from the metabolites observed in the different culture media.

#### Fusarium falciforme

**Fig. 4:**
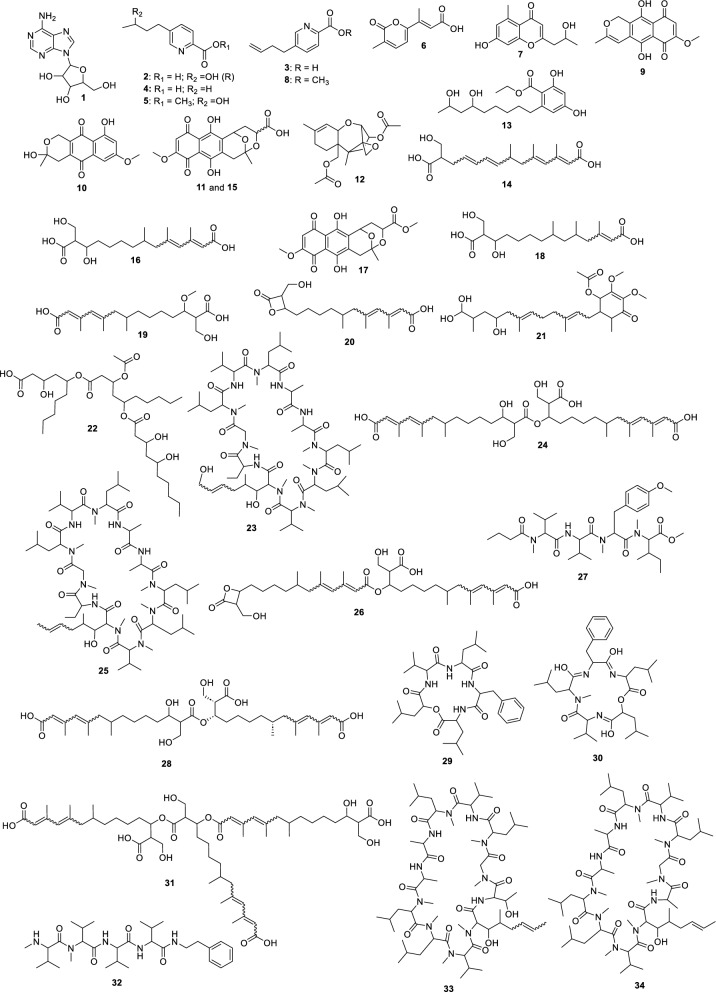
Compounds annotated by molecular networking from the LMC23007.2 fungus. Highlighted compounds (**1**,** 2**,** 3**,** 4**) were isolated and structurally characterized

**Fig. 5:**
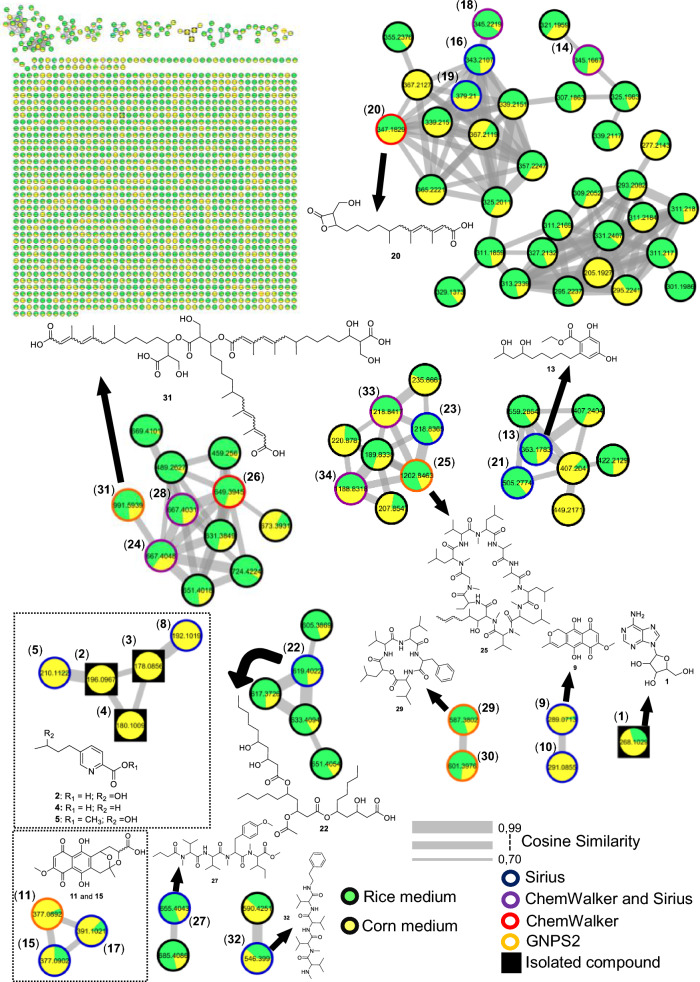
Molecular Network generated by the GNPS2 platform of metabolites produced by the LMC23007.2 fungus in rice and corn culture media

For the fungus LMC23007.2, 34 compounds were annotated (Fig. 4, Table S10), with significant clusters displayed in Fig. 5. A dominant cluster corresponded to the bis-alkenoic class, including fusaridioic acid A (**16**)[37] and its derivatives—compounds well-documented for the *Solani* complex [38] Side-chain esterification via water loss yielded Hymeglusin (**20**) [39] Fragmentation analysis enabled the annotation of additional group members (Fig. 5), revealing diverse derivatives with hydroxylations at varying positions, dehydration, double-bond modifications, and potential dimerization/trimerization, complicating annotation. Nevertheless, computational tools facilitated the identification of metabolites such as halymecin C (**22**) [40] Dimers like fusariumester A1 (**24**) and its isomer *A2* (**28**) were distinguished by retention times [38], while a trimer (**31**) was also detected 2,4-Tetradecadienedioic acid, 12-[[13-carboxy-3-[(13-carboxy-12,14-dihydroxy-3,5,7-trimethyl-1-oxo-2,4-tetradecadien-1-yl)oxy]-2-(hydroxymethyl)-8,10,12-trimethyl-1-oxo-10,12-tridecadien-1-yl]oxy]-13-(hydroxymethyl)-3,5,7-trimethyl.

Fusaric acid derivatives—previously isolated by our group (**2–4**) [41]—were observed alongside esterified forms: Methyl 5-[(3*S*)-3-hydroxybutyl]-2-pyridinecarboxylate (**5**) and methyl dehydrofusarate (**8**) [42]. From the GNPS2 spectral match and ChemWalker, two diastereomers were annotated: isomarticin (**11**, RT 15.23 min) and marticin (**15**, RT 16.09 min), and also of an ester compound formed by acid methylation, annotated as isomarticin methyl ester (**17**) [43] and annotated by SIRIUS. Cyclic depsipeptide, assembled from diverse amino acids like sansalvamide (**29**) [44] and N-methylsansalvamide (**30**) [45], and larger cyclosporin derivatives (A (**23**), C (**33**), and B (**34**)), frequently reported in this fungus [46].

The GNPS2 network revealed metabolite variation across conditions. Rice medium (Fig. 5, green nodes) exhibited higher metabolic diversity and abundance, whereas corn medium induced exclusive compounds, such as the fusaric acid derivative cluster (**4**).

#### Fusarium petroliphilum

**Fig. 6:**
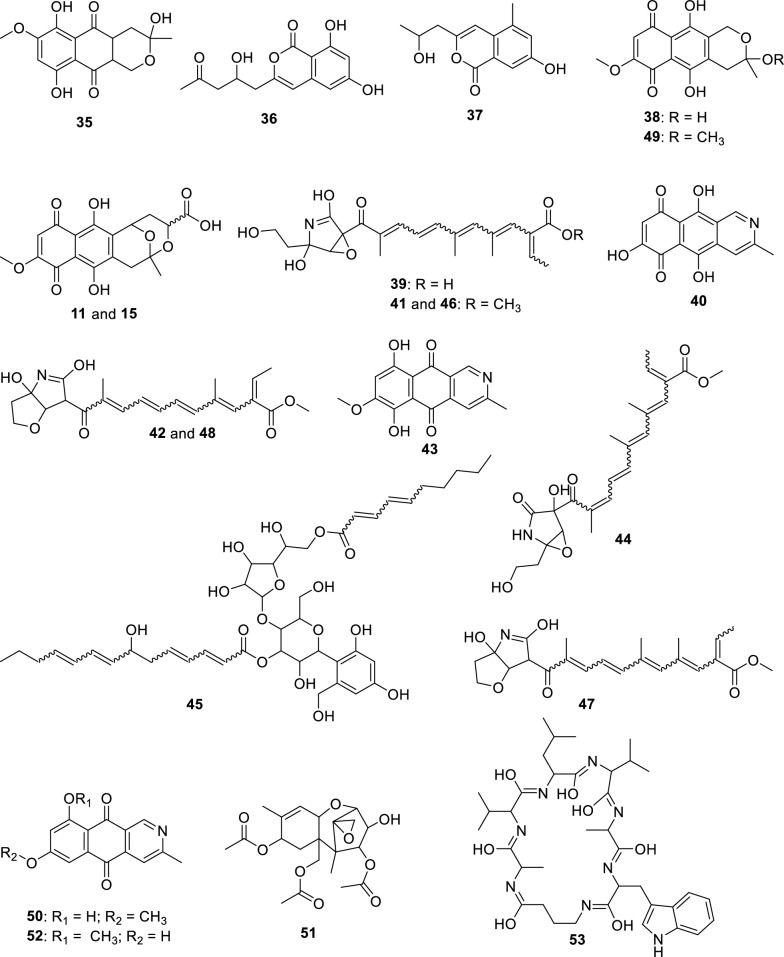
Compounds annotated by molecular networking from the LMC23008 fungus. Highlighted compounds (**35**, **37**, **38**, **40**, **43**, **49**, **50**) were isolated and structurally characterized

**Fig. 7:**
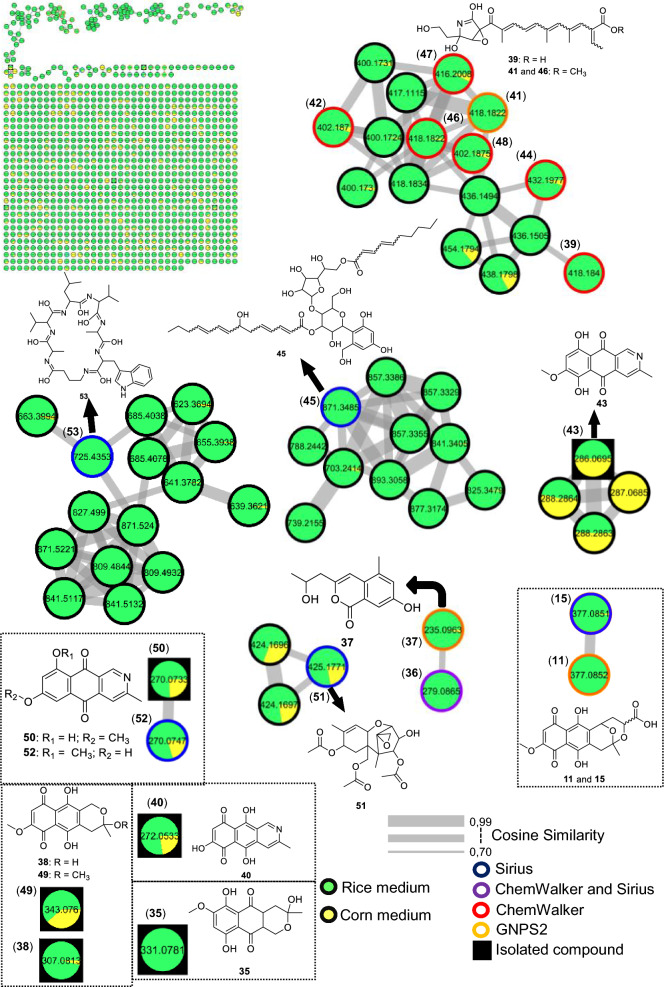
Molecular Network generated by the GNPS2 platform of metabolites produced by the LMC23008 fungus in rice and corn culture media

The fungus LMC23008, another member of the *Solani* complex, yielded the annotation of 21 metabolites, as shown in Fig. 6 and Table S11. Its molecular network revealed a predominant cluster composed mainly of polyketide skeleton, within which GNPS2 enabled the identification of NG-391 (**41**) [47] (Fig. 7). Further ChemWalker analyses annotated six derivatives of this compound, including fusarin A (**47**) [48] and fusarin F (**44**) [48] both were previously reported from *Fusarium* species.

Using SIRIUS, three additional metabolites belonging to distinct clusters were annotated: chaetiacandin (**45**), 8-acetylneosolaniol (**51**) [49] and unguisin B (**53**). No further annotations were obtained for the remaining members of these clusters, suggesting the occurrence of new derivatives or compounds not yet represented in current databases. Despite belonging to the same species complex, LMC23008 and LMC23007.2 shared only two metabolites, isomarticin (**11**) and marticin (**15**) [50].

For LMC23008, a high production of aromatic metabolites was detected. The isolation of some compounds from the fungal extract allowed the identification of anthraquinones and aza-anthraquinones. The first compound (**37**) [51] displayed characteristic ^1^H NMR signals, including resonances typical of an aromatic ring at *δ*_H_ 6.67 (1H, d, *J* = 2.1 Hz) and *δ*_H_ 6.69 (1H, d, *J* = 2.1 Hz), a vinylic hydrogen at *δ*_H_ 5.97 (1H, s), and a hydroxylated proton at *δ*_H_ 4.21 (1H, m). All NMR data for the isolated compounds, which are commonly found in the literature, are presented in the experimental section, together with literature references used for comparison, supporting its identification as 7-hydroxy-3-(2-hydroxypropyl)-5-methyl-epiisocromen-1-one (**37**) [51]. This metabolite was also annotated in the GNPS2 molecular network. Another compound, citreoisocoumarin (**36**) [52] was annotated through SIRIUS and ChemWalker.

Compound (**38**) exhibited a ^1^H NMR spectrum with a vinylic proton conjugated to a carboxyl group at *δ*_H_ 6.46 (1H, s), as well as chelated hydroxyl protons at *δ*_H_ 12.51 (1H, s) and *δ*_H_ 13.00 (1H, s), consistent with an aromatic ring with two hydroxyl groups. Additional non-equivalent methylene protons with geminal coupling constants were observed at *δ*_H_ 2.59 (1H, m) and *δ*_H_ 2.77 (1H, brd, *J* = 17.9 Hz), along with another deshielded CH₂ group adjacent to oxygen at *δ*_H_ 4.66 (1H, dt, *J* = 17.6; 2.3 Hz) and *δ*_H_ 4.72 (1H, d, *J* = 17.6 Hz). Based on these data and comparison with the literature, this metabolite was identified as fusarubin (**38**) [53]. Compound (**49**) displayed similar spectral features to (**38**), with the additional presence of a methoxyl group at *δ*_H_ 3.20 (3H, s). The absence of the *δ*_H_ 6.16 (1H, d, *J* = 1.5 Hz) signal indicated substitution of a hydroxyl group by a methoxyl group. Comparison with the NMR data from literature supported its identification as 3-O-methylfusarubin (**49**) [53]. Compound (**35**) exhibited chelated hydroxyl signals at *δ*_H_ 12.26 (1H, s) and *δ*_H_ 12.00 (1H, s), similar to other members of the series. However, the presence of a distinctive aromatic proton at *δ*_H_ 6.82 (1H, s) suggested a different structural feature. The oxygenated CH₂ group in the cyclic system appeared as a doublet of doublets at *δ*_H_ 4.11 (1H, dd, *J* = 11.4; 4.7 Hz) and *δ*_H_ 3.75 (1H, dd, *J* = 10.9; 11.1 Hz), indicative of vicinal coupling. Additional CH₂ protons were observed at *δ*_H_ 2.30 (1H, dd, *J* = 13.6; 3.6 Hz) and *δ*_H_ 1.70 (1H, dd, *J* = 13.6; 11.4 Hz) with another vicinal coupling. Together with literature comparisons, this compound was assigned as dihydrofusarubin (**35**) [54].

Compound (**40**) presented characteristic signals of a pyridine nucleus conjugated with a carbonyl group at *δ*_H_ 9.29 (1H, s) and *δ*_H_ 7.89 (1H, s), in addition to a vinylic proton at *δ*_H_ 5.57 (1H, s). A singlet at *δ*_H_ 2.66 (3H, s) corresponded to a methyl group bound to an unsaturated system. Based on ^1^H NMR from the literature, the metabolite was identified as 5,7,10-trihydroxy-3-methylbenzo[g]isoquinoline-6,9-dione (**40**). Compound (**43**) also exhibited diagnostic protons of a pyridine nucleus at *δ*_H_ 9.38 (1H, s) and *δ*_H_ 8.00 (1H, s), as well as an aromatic hydrogen at *δ*_H_ 6.97 (1H, s), consistent with an aza-anthraquinone skeleton. Chelated hydroxyl groups were confirmed by the signals at *δ*_H_ 13.51 (1H, s) and *δ*_H_ 13.10 (1H, s). These features, together with comparative NMR data from literature, enabled its identification as bostrycoidin (**43**) [53].

A structurally related metabolite displayed the same aza-anthraquinone skeleton, but with an additional aromatic hydrogen, evidenced by coupling at *δ*_H_ 7.34 (1H, d, *J* = 2.5 Hz) and *δ*_H_ 6.86 (1H, d, *J* = 2.5 Hz), indicating the absence of one hydroxyl group when compared with compound (**43**). Comparison with reported data supported its assignment as 5-deoxybostrycoidin (**50**) [55]. This metabolite formed a cluster with its positional isomer (**52**), which exhibited the same molecular mass but a slightly different retention time, and was annotated through SIRIUS as 7-O-desmethylescorpinone.

The molecular networking analysis demonstrated that the main secondary metabolites were produced in rice medium (highlighted in green, Fig. 7), while only a few were detected in corn medium (highlighted in yellow). Bostrycoidin (**43**) was the dominant metabolite in corn medium, accompanied by several derivatives that formed a molecular cluster, although these compounds could not be annotated yet.

#### Fusarium sacchari isolated from papaya

**Fig. 8:**
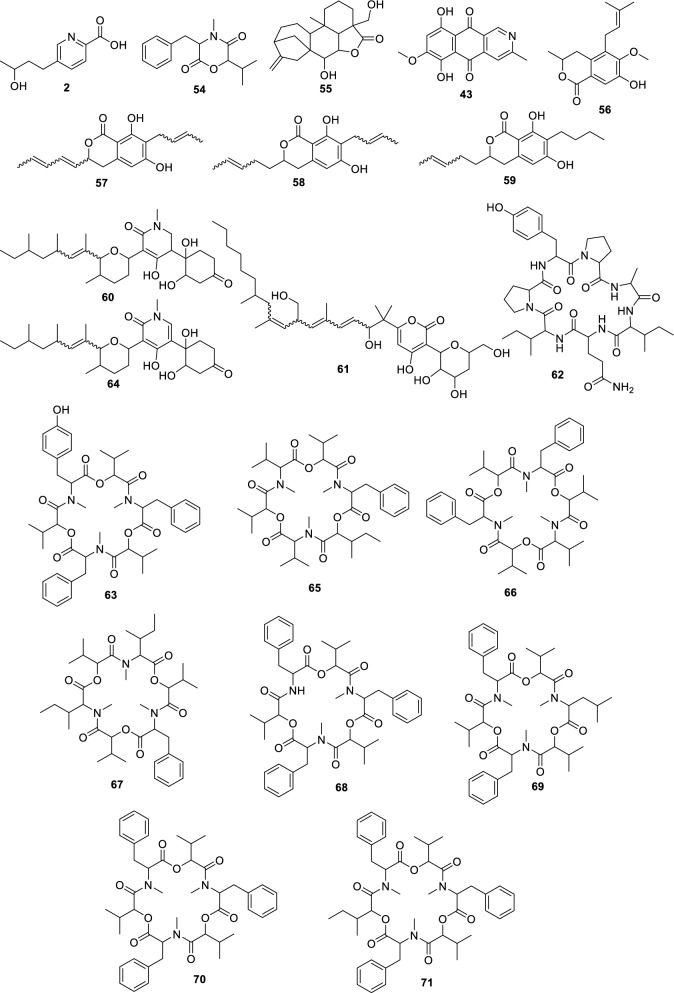
Compounds annotated by molecular networking from the LMC23012 fungus. Highlighted compounds (**2**, **43**, **57**, **58**, **59**, **70**) were isolated and structurally characterized

**Fig. 9:**
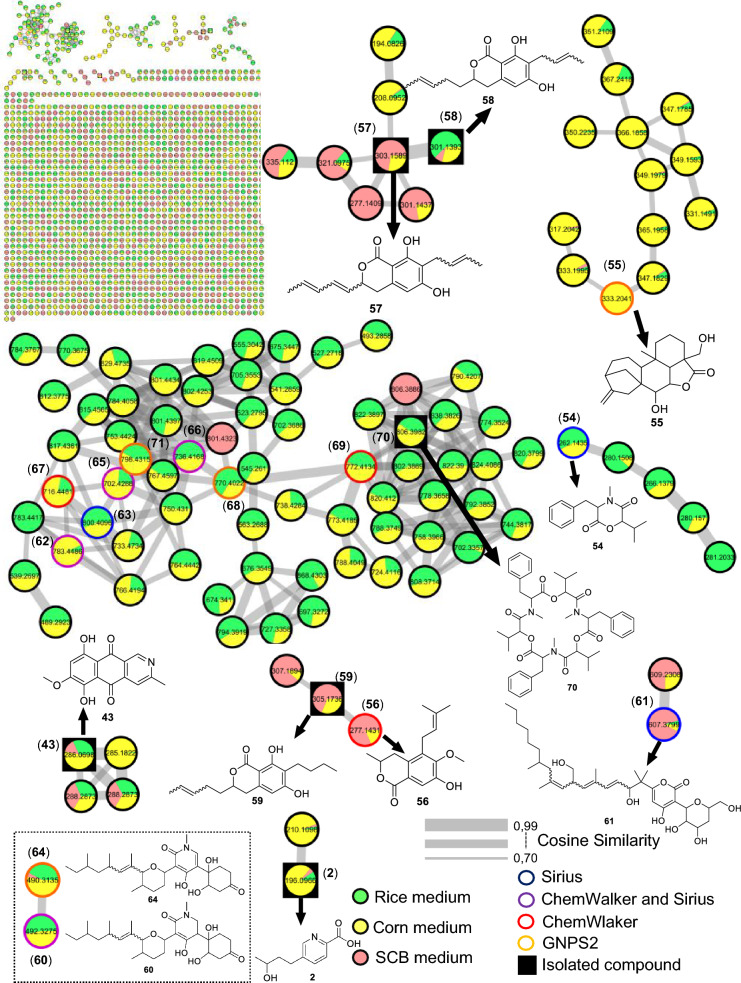
Molecular Network generated by the GNPS2 platform of metabolites produced by the LMC23012 fungus in rice and corn culture media

The species LMC23012, belonging to the *Fusarium fujikuroi* species complex, exhibited a diversified metabolic profile, with 20 compounds annotated (Fig. 8, Table S12). Molecular networking analysis (Fig. 9) revealed that the predominant cluster consists of beauvericin derivatives.

The fungal extract separation primarily yielded beauvericin (**70**), a well-characterized metabolite previously reported by our group and commonly described in *Fusarium* species [20]. GNPS2 analysis facilitated the annotation of beauvericin analogs, including beauvericin D (**68**)[52] and beauvericin A (**71**) [56].

Further structural elucidation via ChemWalker and SIRIUS led to the identification of six additional derivatives. However, several cyclic depsipeptides remained uncharacterized, due to closely similar molecular masses, suggesting subtle variations in amino-acid positioning or substitution within the core structure. Notably, production profiles varied significantly among different culture media. In both rice-based (green, Fig. 9) and corn-based (yellow) media, the production of beauvericin derivatives was balanced and occurred in similar proportions. In contrast, cultivation on SCG (pink) led to a dramatic reduction in these metabolites, with only two compounds detected within the cluster. These two metabolites appeared to be unique to the SCB medium, indicating a distinct metabolic response to the nutritional environment provided by this substrate.

Among the identified metabolites, fusarinolic acid (**2**) and bostrycoidin (**43**)—both previously reported in other fungal species—showed higher production in corn-based medium for this isolate, whereas lower quantities were observed in SCB medium.

Structural analysis of a separate cluster uncovered a metabolite containing the distinctive N-methyl-4-hydroxy-2-pyridone core, annotated as oxo﻿sporidinone (**64**) [57] through GNPS. A structurally related compound, featuring two additional hydrogen atoms, was characterized as an oxo﻿sporidinone derivative (**60**) using SIRIUS and ChemWalker.

Within the class of diketomorpholines, bassiatin (**54**) [58] was annotated via SIRIUS, although several other cluster constituents remain unresolved. A distinct cluster, predominantly composed of compounds produced in corn medium (with minor presence in rice medium), enabled the annotation of the diterpenoid lactone 7,19-dihydroxy-6,18-epoxicaur-16-en-18-one (**55**) [59] via GNPS, while the identities of other cluster members remain to be determined. Fusapyrone (**61**) [60] was predominantly detected in the SCB medium and annotated through the SIRIUS platform.

Compound (**57**) exhibited distinct spectroscopic features in ^1^H NMR (Fig. S8) an aromatic proton at *δ*_H_ 6.37 (s, 1H) and a conjugated *trans*-double bond system evidenced by signals at *δ*_H_ 5.47 (dd, *J* = 6.3, 15.7 Hz), *δ*_H_ 6.12 (dd, *J* = 10.4, 15.7 Hz), *δ*_H_ 6.40 (dd, *J* = 10.4, 15.3 Hz), and *δ*_H_ 5.83 (dq, *J* = 6.8, 15.3 Hz). Additional olefinic protons were observed at *δ*_H_ 5.55 (dq, *J* = 1.4, 6.3, 15.3 Hz) and *δ*_H_ 5.73 (dd, *J* = 6.5, 15.3 Hz). Multiplets at *δ*_H_ 2.97 and 2.88 (1H each) suggested a cyclic CH₂ group, while a signal at *δ*_H_ 5.10 (brdd, *J* = 6.3, 12.2 Hz, 1H) is consistent with an ester moiety within a six-membered lactone ring. Comparison with published data allowed assignment of this compound (**57**) as 7-but-15-enyl-6,8-dihydroxy-3(R)-penta-9,11-dienylisocoumarin [61].

A structurally related compound, designated as (**58**), exhibited a similar ^1^H NMR (**Fig. S9**) profile but lacked the conjugated double bond system. High-resolution mass spectrometry confirmed the presence of two additional hydrogen atoms relative to compound (**57**), permitting assignment of (**58**) as 7-but-2-enyl-6,8-dihydroxy-3-pent-3-enyl-3,4-dihydroisocoumarin [62]. Both metabolites clustered together in the molecular network, sharing analogous fragmentation patterns, although other related metabolites in the group remain uncharacterized.

A third derivative, compound (**59**), showed structural variation via hydrogenation of the side chain, yielding an *n*-butyl group—supported by proton signals at *δ*_H_ 2.62 (t, *J* = 7.5 Hz, 2H), 1.51 (quint, *J* = 7.5 Hz, 2H), 1.36 (sext, *J* = 7.4 Hz, 2H), and 0.91 (t, *J* = 7.4 Hz, 3H). Comparison across all NMR data (Fig. S10) with literature enabled its identification as 7-butyl-6,8-dihydroxy-3(R)-pent-11-enylisochroman-1-one [62]. This structural shift yielded a divergent mass fragmentation pattern that placed compound (**59**) in a separate cluster. Through complementary analyses using ChemWalker, a fourth, non-isolated derivative was annotated as versicoumarin C (**56**), further enriching the structural scope of this metabolite class.

Comparative profiling across cultivation media revealed marked differences in specialized metabolite production. While rice and corn media favored the predominant biosynthesis of beauvericin derivatives (Fig. 9), cultivation in SCB medium led to production of isocoumarin derivatives (compounds **56–59**), coincident with a substantial decline in beauvericin synthesis (highlighted in pink in Fig. 9). This metabolic shift underscores the plasticity of secondary metabolism in *Fusarium* sp, demonstrating its ability to activate distinct biosynthetic pathways in response to nutritional ingredients. These results highlight the strategic importance of medium selection in accessing the chemical diversity of filamentous fungi and advancing their biotechnological potential for the discovery of new compounds.

#### Fusarium sacchari isolated from pineapple

**Fig. 10:**
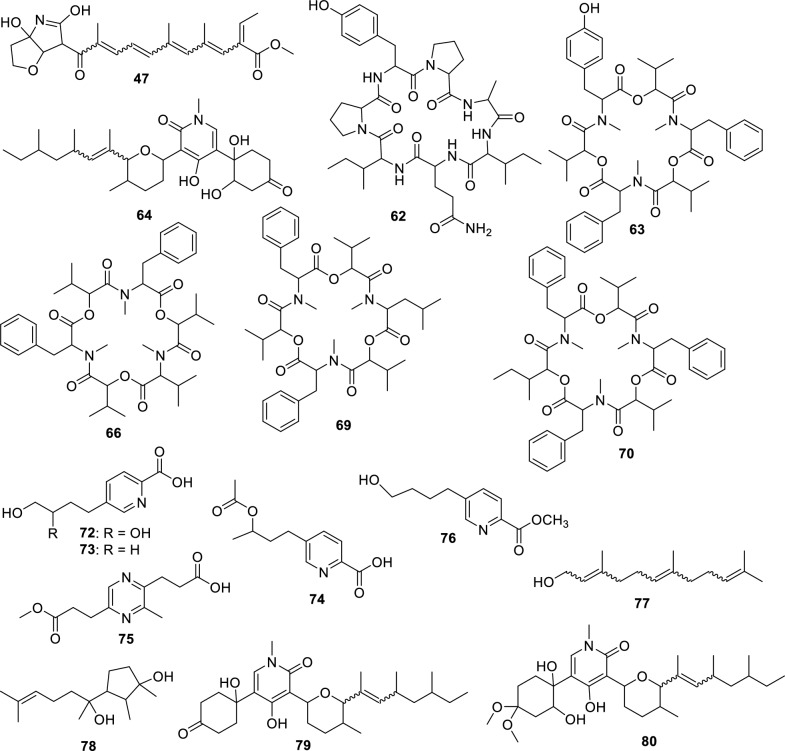
Compounds annotated by molecular networking and isolated from the LMC23015 fungus

**Fig. 11:**
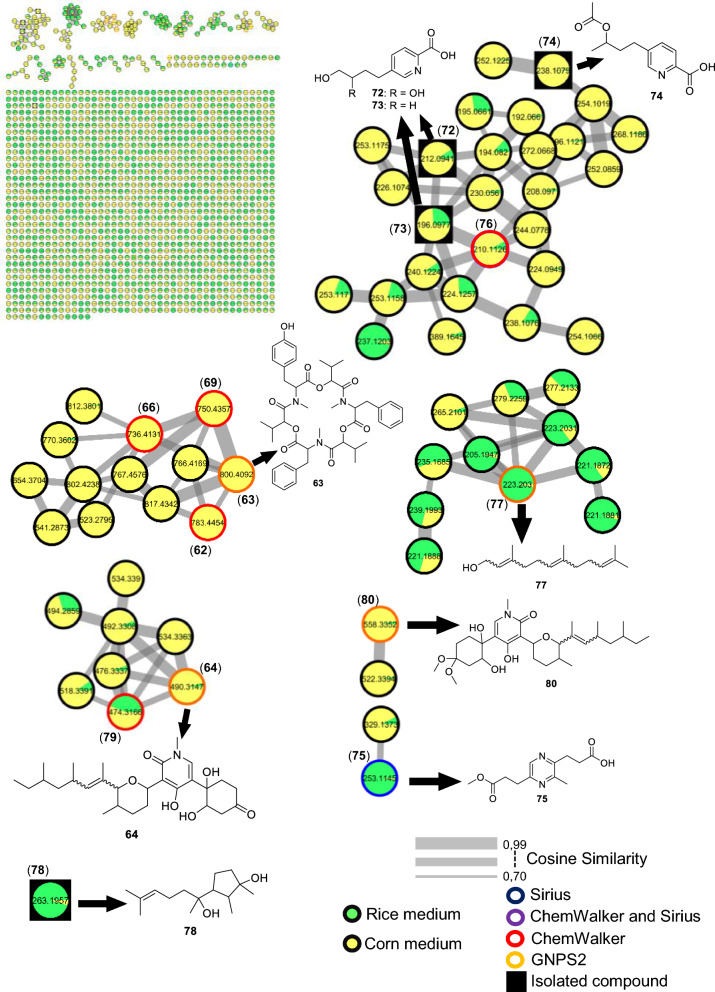
Compounds annotated by molecular networking from the LMC23015 fungus. Highlighted compounds (**72**, **73**, **74**, **70**, **78**) were isolated and structurally characterized

The fungus LMC23015 exhibited a markedly distinct chemical profile, characterized by 16 annotated metabolites (Fig. 10 and Table S13). Its major metabolite featured a pyridine core (Fig. 11), structurally related to the mycotoxin fusaric acid (**4**), which was not detected in this cluster, although its derivatives were isolated.

Among the other investigated fungi, the compound fusarinolic acid (**2**) was obtained, corresponding to fusaric acid bearing a hydroxyl group at C-9, with a retention time (rt) of 2.44 min, previously characterized by our group in earlier studies. However, in *F. sacchari*, a new analogue was identified, featuring hydroxylation at C-10, with rt of 2.45 min. This structure was confirmed by ^1^H NMR spectroscopy, which showed a signal at *δ*_H_ 4.04 (t, *J* = 6.0 Hz, 2H), corresponding to the terminal protons of the *n*-butyl chain. In addition, the signals at *δ*_H_ 8.12 (d, *J* = 7.9 Hz, 1H), *δ*_H_ 7.92 (dd, *J* = 7.9 Hz, 1H), and *δ*_H_ 8.45 (s, 1H) confirmed the presence of a disubstituted pyridinic nucleus, without further significant modifications. Based on these data, compound **73** was identified as 10-hydroxyfusaric acid.

Another metabolite isolated exhibited three resonances for oxymethine and oxymethylene protons: one at *δ*_H_ 3.74 (m, 1H) and two geminally coupled at *δ*_H_ 3.52 (dd, *J* = 11.2; 5.8 Hz, 1H) and *δ*_H_ 3.57 (dd, *J* = 11.2; 4.8 Hz, 1H), consistent with a CH₂–O group. The pyridinic core signals remained unchanged; however, hydroxylation of the *n*-butyl side chain generated a chiral center, rendering the CH₂ protons magnetically nonequivalent and resulting in distinct chemical shifts. These observations supported the assignment of compound **72** as 5-(3,4-dihydroxybutyl)-2-pyridinecarboxylic acid [63].

In addition, a derivative of this class was isolated in this cluster. This compound showed signals analogous to those of fusarinolic acid (**2**), except for a characteristic resonance at *δ*_H_ 4.88 (dq, *J* = 12.5; 6.3 Hz, 1H), suggestive of an ester functionality. The signal at *δ*_H_ 1.98 (s, 3H) indicated an additional methyl group α to a carbonyl, supporting the incorporation of an acetate moiety. Accordingly, compound **74** was identified as 2-pyridinecarboxylic acid, 5-[3-(acetyloxy)butyl].

Finally, compound **76**, annotated via ChemWalker, exhibited spectral similarity to **73**, but with a pattern consistent with an ester instead of a free acid, and was assigned as methyl 5-(4-hydroxybutyl)-2-pyridinecarboxylate.

Two additional compounds were annotated in GNPS2: fusarin C, observed in scan 747, and *epi*-fusarin C, observed in scan 794. Although these are compounds known to be isolated from *Fusarium*, differences in acquisition energy resulted in mass spectra with significant discrepancies in ion intensity.

For the *Fusarium* strain LMC23015, metabolites analogous to those previously observed were annotated, including a cluster of beauvericin derivatives, with beauvericin itself (**70**) being isolated from this organism. The other metabolites produced were identical to those described for the other *F. sacchari* isolate and are therefore not discussed again. It is noteworthy, however, that the production of beauvericin and its derivatives was significantly higher in corn medium (Fig. 11, yellow). Another compound common to both fungi was oxysporidinone (**64**). In this cluster, nevertheless, a new metabolite was annotated, featuring one less oxygen atom and annotated via ChemWalker as (–)-6-deoxyoxysporidinone (**79**). In addition, a distinct cluster was detected, retaining the same core skeleton as **64** but with the ketone moiety replaced by two methoxyl groups, resulting in an altered fragmentation pattern. GNPS2 designated this group the as dimethyl ketal of oxysporidinone (**80**), reported for *Fusarium oxysporum*. The production of these metabolites within their respective clusters was predominantly observed in corn medium, representing a distinct pattern compared with the other fungi studied, for which most compounds were produced exclusively in this medium.

In rice medium, GNPS2 detected the presence of farnesol (**77**), a common sesquiterpene, whose cluster likely corresponds to structural derivatives, although no additional metabolites were confidently annotated. Finally, the last metabolite isolated with major production in rice medium was cyclonerodiol (**78**) [41] previously characterized by our group in studies with *Fusarium* species.

#### Fusarium verticillioides

**Fig. 12:**
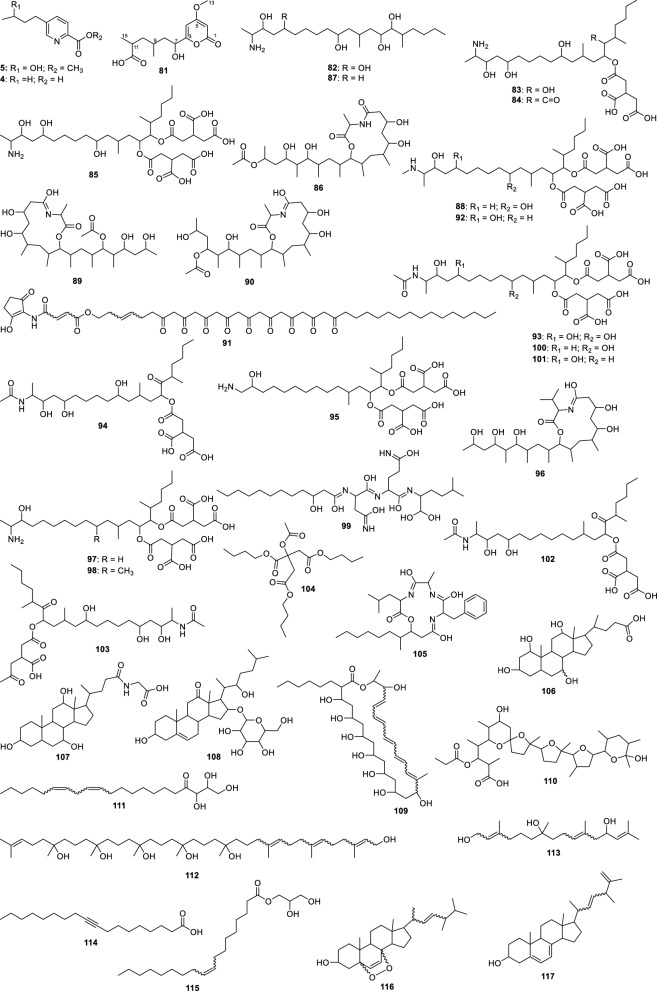
Compounds annotated by molecular networking from the LMC23018 fungus. Highlighted compounds (**4**, **81**) were isolated and structurally characterized

For the *Fusarium* strain LMC23018, a total of 40 metabolites were annotated, representing the greatest chemical diversity among the *Fusarium* species investigated (Fig. 12 and Table S14).

**Fig. 13:**
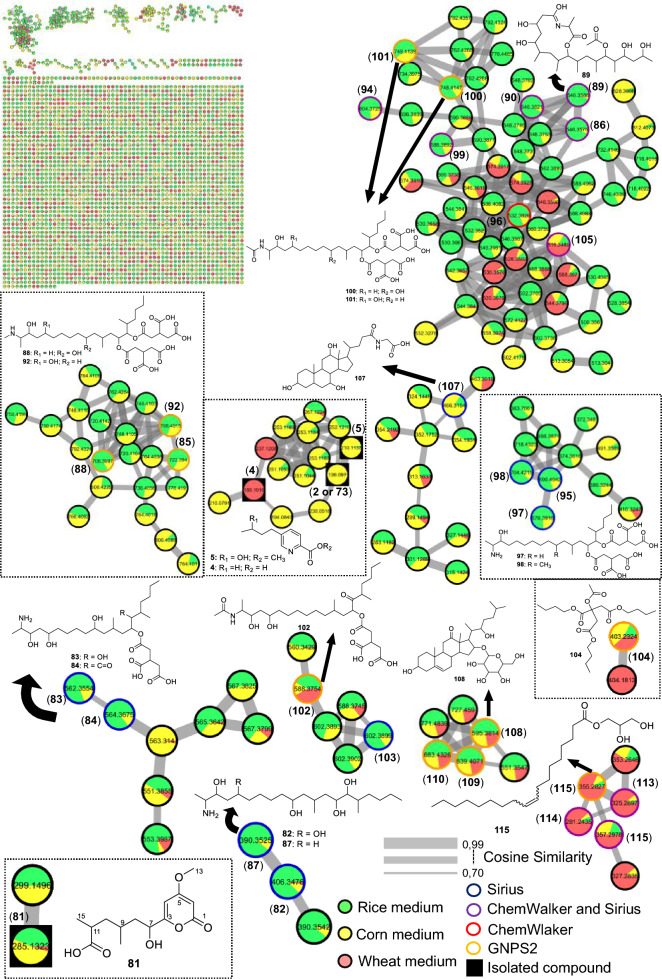
Molecular Network generated by the GNPS2 platform of metabolites produced by the LMC23018 fungus in rice and corn culture media

In the molecular network, a cluster of fusaric acid derivatives was annotated, consistent with reports for other fungi. Notably, these metabolites were predominantly produced in corn medium, while two derivatives, including fusaric acid itself (**4**), were produced exclusively in wheat medium (Fig. 13).

Across most clusters, metabolites derived from polyketides with long hydroxylated chains were observed. Variations in hydroxylation, the position and degree of esterification, the presence of amino or amide groups, as well as differences in chain length, generated distinct clusters. For these metabolites, the SIRIUS and ChemWalker platforms were essential for structure differentiation and annotation.

The major cluster included two metabolites annotated by GNPS, fumonisins A_2_ (**101**) [64] and A_3_ (**100**), which differ in the position of a hydroxyl group. Due to the complexity of this cluster, variations among more distant nodes revealed significant chemical diversity. Thermolide derivatives were also annotated, including thermolides A (**90**), B (**86**), C (**89**), and E (**96**), by ChemWalker and SIRIUS, corresponding to cyclic ester forms of these polypeptides. Additional noteworthy metabolites in this cluster included fellutamide F (**99**) and beauverolide L (**105**). Importantly, most metabolites in this cluster were predominantly produced in rice medium.

Another relevant cluster consisted of fumonisin derivatives containing an amino group replacing the characteristic amide group covering the fumonisin B series. In this group, GNPS2 enabled the annotation of fumonisins B_1_ (**85**) [65], B_2_ (**92**) [66] and B_3_ (**88**) [67] which differ in the number and position of hydroxyl substituents. Fumonisin B_4_ (**97**) [68] however, contains a primary amino group and was distinguished from this series, clustering instead with fumonisin C_4_ (**95**) [69] both annotated by SIRIUS.

Derivatives of fumonisin A were annotated in another cluster, characterized by the absence of one propano-1,2,3-tricarboxylic acid unit, as exemplified by fumonisin AK1 (**103**). A similar situation was observed for fumonisin B derivatives, where a cluster lacking one propano-1,2,3-tricarboxylic acid unit was annotated, including desacylfumonisin B_1_ (**82**) [67] and fumonisin PH1b (**83**) [70]. Finally, a cluster containing metabolites lacking both propano-1,2,3-tricarboxylic acid units was detected, likely formed by hydrolysis of the original compounds, such as desacylfumonisin B_1_ (**82**) and hydrolyzed fumonisin B_3_ (**87**).

Fumonisin A_1_ was also annotated but did not group into a cluster, appearing as a singleton. Other molecules were annotated and can be seen in Table S14 and the clusters of Fig. 13, although only the major ones are discussed here. An additional cluster of interest, not belonging to the fumonisin class, included monoolein (**115**), annotated by GNPS. Furthermore, four other metabolites were annotated by SIRIUS and ChemWalker, being produced in greater abundance in wheat medium (red).

Due to the extensive diversity of fumonisin derivatives and other metabolites, the individual isolation of these compounds was not possible, as the fractions contained mixtures of three to four fumonisins, as evidenced by ^1^H NMR. Nevertheless, a new compound was successfully isolated, and its structure was determined to be unreported in the scientific literature.

Fractionation of the corn medium led to the isolation of a metabolite whose ^1^H NMR spectrum (Fig. S11, Table 2) displayed protons of two olefinic protons at *δ*_H_ 6.06 (1H, d, *J* = 2.0 Hz) and *δ*_H_ 5.39 (1H, d, *J* = 2.0 Hz), exhibiting a ^4^* J* coupling and chemical shifts consistent with a disubstituted six-membered lactone ring. A methoxyl signal was observed at *δ*_H_ 3.77 (3H, s), showing HMBC correlation (Fig. S12) with a carbon at *δ*_C_ 170.12, thereby linking it to the lactone moiety. A proton at *δ*_H_ 4.40 (dd, *J* = 10.1, 2.9 Hz, 1H) was attributed to a hydroxylated proton, coupling with two neighboring hydrogens. Through HMBC correlations, this proton showed connectivity to a carbon at *δ*_C_ 166.16, indicating an additional substitution on the ring. Two methyl doublets were also detected at *δ*_H_ 0.93 (d, *J* = 6.5 Hz, 3H) and *δ*_H_ 1.13 (d, *J* = 6.9 Hz, 3H). The latter showed HMBC correlation with a carbon at *δ*_C_ 180.16, suggesting proximity to a carboxyl group. Both methyl groups correlated only with a single carbon at *δ*_C_ 41.82, which, according to HSQC (Fig. S13), was bound to protons at *δ*_H_ 1.57 (1H, m) and *δ*_H_ 1.76 (1H, m).

**Table 2:** NMR data from compound **81** in CDCl₃, 500 MHz. In which ^1^H NMR data are reported as chemical shift (*δ*) in ppm (integration, multiplicity, and coupling constant (J) in Hz)

N	^1^H NMR	^13^C NMR	HMBC
1	–	164.48	–
3	–	166.16	–
4	6.06 (1H, d, *J* = 2.0)	98.96	170.12; 166.16; 68.65; 88.11
5	–	170.12	–
6	5.39 (1H, d, *J* = 2.0)	88.11	170.12; 164.48; 98.96
7	4.40 (1H, dd, *J* = 10.1, 2.9)	68.65	166.16; 98.96; 41.82; 27.16
8a	1.57 (1H, m)	41.82	166.16; 68.65; 41.08; 27.16; 19.42
8b	1.76 (1H, m)		
9	1.79 (1H, m)	27.16	–
10a	1.32 (1H, m)	41.08	180.16; 41.82; 36.26; 27.16; 19.42; 17.42
10b	1.68 (1H, m)		
11	2.54 (2H, td, *J* = 13.7, 6.9)	36.26	180.16; 41.08; 27.16; 17.13
12	–	180.16	–
13	3.77 (3H, s)	56.16	170.12
14	0.93 (3H, d, *J* = 6.5)	19.42	41.82; 27.16
15	1.13 (3H, d, *J* = 6.9)	17.13	180.16; 41.82; 36.26

**Fig. 14:**
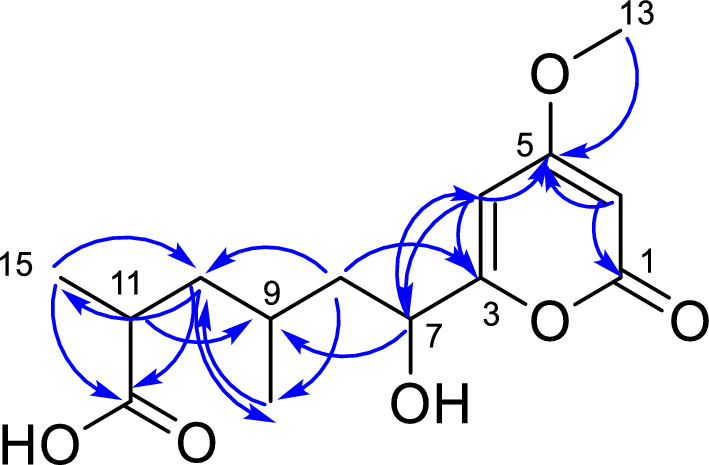
All HMBC correlations for compound **81**

Table 2 summarizes all proton resonances, their correlated carbons (HSQC), and additional long-range HMBC correlations. Using 2D NMR data, the complete carbon chemical shift of the molecule was assigned, as illustrated in Fig. 14, which depicts the experimentally observed HMBC correlations.

Based on these spectroscopic data, the compound was determined to contain an acyl-hexanoic side chain with two additional methyl groups and one and one hydroxyl group. The metabolite was identified as 6-hydroxy-6-(4-methoxy-2-oxo-2H-pyran-6-yl)-2,4-dimethylhexanoic acid (**81**), described here for the first time. Compound **81** differs from previously reported analogs by the presence of a carboxylic acid group at the end of the side chain and methyl groups attached to this chain. In contrast, literature-described compounds feature side chains of varying lengths and a hydroxyl group positioned near the lactone ring.

In the molecular network, a related cluster was detected, which included an analogue with 14 mass units less, consistent with the absence of a CH₂ group. This observation suggests the presence of a second possible new compound, although it was not isolated.

#### Fusarium guttiforme

**Fig. 15:**
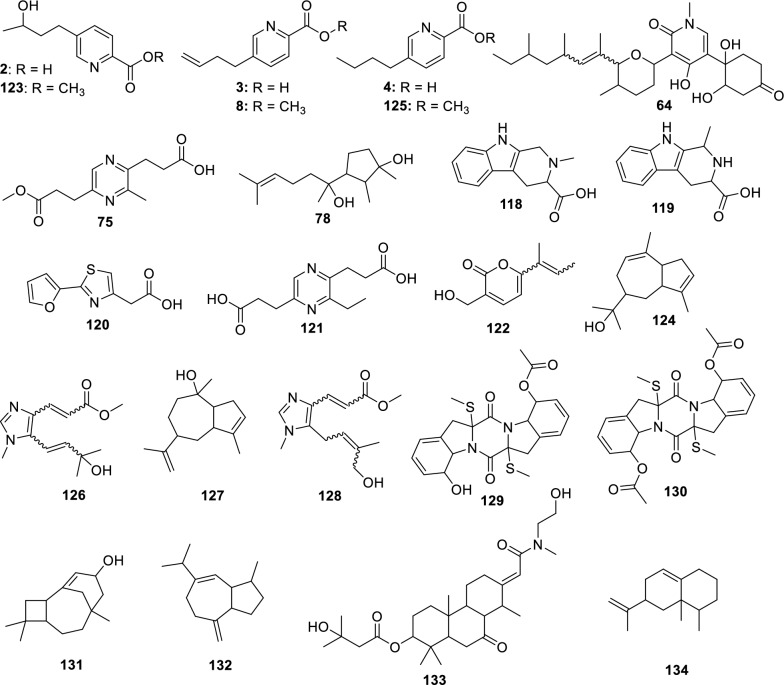
Compounds annotated by molecular networking from the F1 fungus. Highlighted compounds (**2**,** 3**,** 4**, **78**, **129**, **130**) were isolated and structurally characterized

**Fig. 16:**
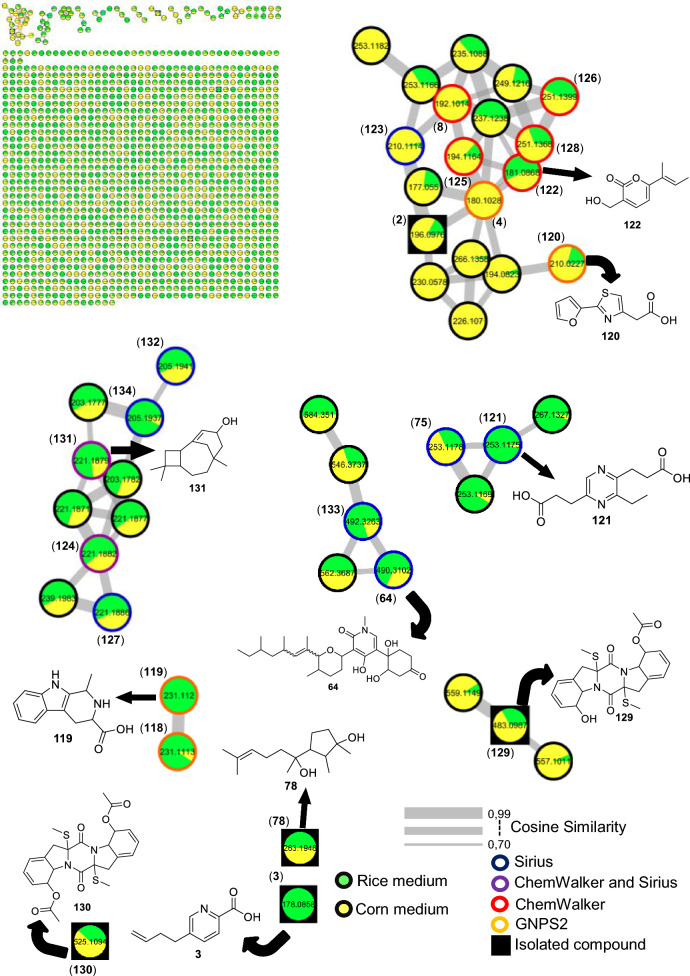
Molecular Network generated by the GNPS2 platform of metabolites produced by the F1 fungus in rice and corn culture media

For F1, a total of 24 metabolites were annotated, as shown in Fig. 15 and Table S15. A cluster containing fusaric acid derivatives, previously reported for other fungi, was observed (Fig. 16). However, in a previous study by our group, the compounds fusarinolic acid (**2**), 9,10-dehydrofusaric acid (**3**), and fusaric acid (**4**) were isolated from this fungus when cultivated in rice and potato dextrose broth (PDB) media [41] Notably, this is the first report of these metabolites being produced in corn medium for this species.

In this cluster, other low-molecular-weight metabolites were annotated, including 2-(2-furanyl)-4-thiazoleacetic acid (**120**) by GNPS, and, by ChemWalker, the compounds fusarpyrone A (**122**) [71] fusagerin C (**126**) [72] and hydroxyfungerin A (**128**), all previously described in *Fusarium* species.

A cluster of sesquiterpene derivatives was also annotated, with five metabolites annotated, including 3-caryolen-5-ol (**131**) and Guaia-6,10(14)-diene (**132**) [73] both were previously isolated from *Fusarium* species. Except for strain LMC23018, all strains of the *Fujikuroi* complex produced oxysporidinone (**64**), including this fungus. However, a new derivative, not found before, norcoumingide (**133**), was annotated by SIRIUS.

Another relevant cluster contained a metabolite distinct from those of the others fungi, annotated as 3-ethyl-2,5-pyrazinedipropanoic acid (**121**), bearing an additional ethyl substituent. Two compounds, annotated by GNPS2 and produced almost exclusively in rice medium, were 2-methyl-3-carboxy-1,2,3,4-tetrahydroharman (**118**) and 1,2,3,4-tetrahydro-3-carboxyharmane (**119**), which differ by the position of a methyl group.

The metabolites haematocin (**130**) and cyclonerodiol (**78**) had already been isolated by our group from this fungus growing in ISP2 and rice media, respectively [41, 74]. Nevertheless, this is the first report of haematocin (**130**) production in rice and corn media. Similarly, cladosporin (**129**) had previously been annotated in ISP2 medium, but was now annotated in both corn and rice media. No additional metabolites were annotated in its cluster.

In total, 134 metabolites were annotated across the six *Fusarium* species studied, of which 117 were assigned to the isolates obtained in this work from papaya and pineapple. A comparative analysis of secondary metabolite production by different *Fusarium* species (Table 3), cultivated on distinct substrates (rice, corn, wheat, and SCB), revealed occurrence patterns that suggest both biosynthetic conservation among species and differential induction based on the culture medium.

**Table 3:** Metabolites from literature isolated from *Fusarium* spp. cultivated on different substrates

Compound	Name	Strains + culture medium
**2**	Fusarinolic acid	LMC23007.2 – Rice, Corn; LMC23012 – Rice, Corn, SCB; F1 – Rice, Corn
**3**	9,10-Dehydrofusaric acid	LMC23007.2 – Rice, Corn; F1 – Rice, Corn
**4**	Fusaric acid	LMC23007.2 – Rice, Corn; LMC23018 – Rice, Corn, Wheat; F1 – Rice, Corn
**5**	Fusarinolic acid methyl ester	LMC23007.2 – Rice, Corn; LMC23018 – Rice, Corn, Wheat
**8**	9,10-Dehydrofusarinolic acid methyl ester	LMC23007.2 – Rice, Corn; F1 – Rice, Corn
**11**	Isomarticin	LMC23007.2 – Rice, Corn; LMC23008 – Rice, Corn
**15**	Marticin	LMC23007.2 – Rice, Corn; LMC23008 – Rice, Corn
**43**	Bostrycoidin	LMC23008 – Rice, Corn; LMC23012 – Rice, Corn, SCB
**47**	Fusarin A	LMC23008 – Rice, Corn; LMC23015 – Rice, Corn
**62**	Stylissamide E	LMC23012 – Rice, Corn, SCB; LMC23015 – Rice, Corn
**63**	Beauvericin J	LMC23012 – Rice, Corn, SCB; LMC23015 – Rice, Corn
**64**	Oxysporidinone	LMC23012 – Rice, Corn, SCB; LMC23015 – Rice, Corn; F1 – Rice, Corn (universal in rice)
**66**	Beauvenniatin A	LMC23012 – Rice, Corn, SCB; LMC23015 – Rice, Corn
**69**	Beauvericin K	LMC23012 – Rice, Corn, SCB; LMC23015 – Rice, Corn
**70**	Beauvericin	LMC23012 – Rice, Corn, SCB; LMC23015 – Rice, Corn
**75**	Dimethyl 3,3'-(pyrazine-2,5-diyl)dipropanoate	LMC23015 – Rice, Corn; F1 – Rice, Corn
**78**	Cyclonerodiol	LMC23015 – Rice, Corn; F1 – Rice, Corn

^*^More information on the compounds is provided in the Supplementary Material

Fusaric acid derivatives (fusarinolic acid (**2**), 9,10-dehydrofusaric acid, fusaric acid (**3**), and methylated derivatives (**5** and **8**)) were detected predominantly in LMC23007.2 and F1 cultivated on rice and corn, as well as in MC23018 on rice, corn, and wheat. This broad distribution demonstrates that these metabolites represent a conserved biosynthetic pathway within the *Fusarium* genus, being strongly induced in cereals.

The compounds isomarticin (**1**1) and marticin (**15**) were common to LMC23007.2 and LMC23008 cultivated on rice and corn, suggesting that the biosynthesis of these phenolic derivatives may be associated with specific grain-cultivation conditions for the *Solani* complex.

Bostrycoidin (**43**) and the polyketide fusarin A (**47**) showed distinct occurrence patterns: while the former was identified in LMC23008 (rice and corn) and LMC23012 (rice, corn, and SCB), the latter was present in LMC23008 and LMC23015 cultivated on rice and corn. These results indicate that the production of this polyketide metabolite is more linked to the species than to the substrate, as it was produced on all tested media for these isolates.

The cyclic peptides (stylissamide E (**62**), beauvericin J (**63**), beauvericin K (**69**), beauvericin (**70**), and beauveniatin A (**66**)) were particularly abundant in LMC23012 and LMC23015 cultivated on diversified substrates (rice, corn, and SCB), indicating that these compounds are linked to these species, being obtained exclusively in *F. sacchari*, regardless of the medium. Oxysporidinone (**64)**, however, was detected in the majority of species cultivated on rice, representing a common metabolite.

Finally, less common compounds, such as dimethyl 3,3'-(pyrazine-2,5-diyl)dipropanoate (**75**) and cyclonerodiol (**78**), also showed a shared pattern among distinct species (LMC23015 and F1), again under cultivation on rice and corn. This reinforces the hypothesis that cereal-based substrates act as fundamental triggers for the expression of secondary metabolic pathways in *Fusarium*.

#### Neofusicoccum ribis

**Fig. 17:**
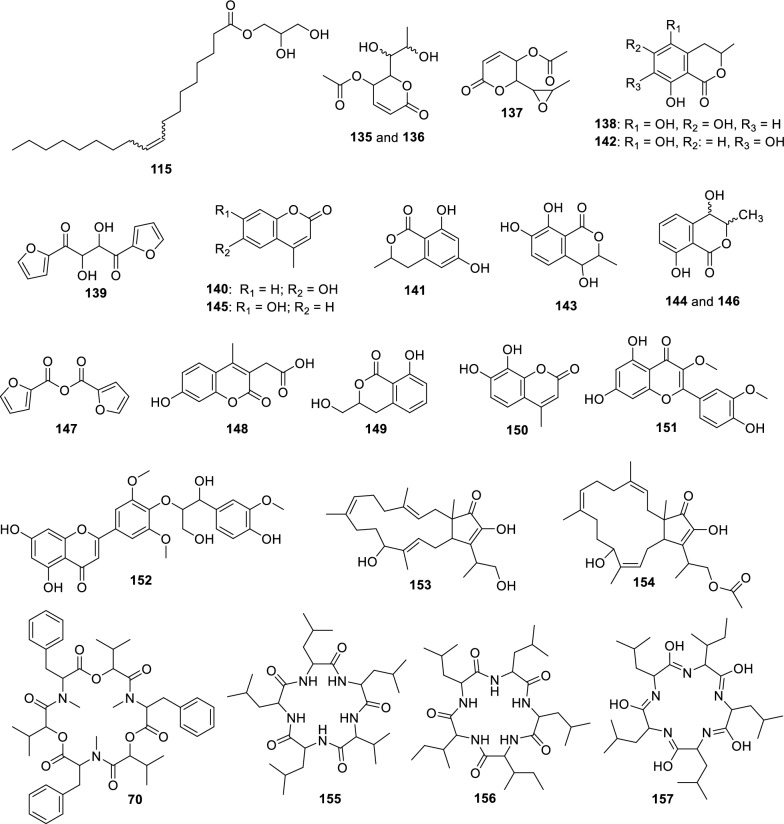
Compounds annotated by molecular networking from the LMC23006 fungus. Highlighted compounds (**70**, **137**, **139**, **147**) were isolated and structurally characterized

For strain LMC23006, an endophyte isolated from papaya, 24 metabolites were annotated, as shown in Fig. 17 and Table S16.

**Fig. 18:**
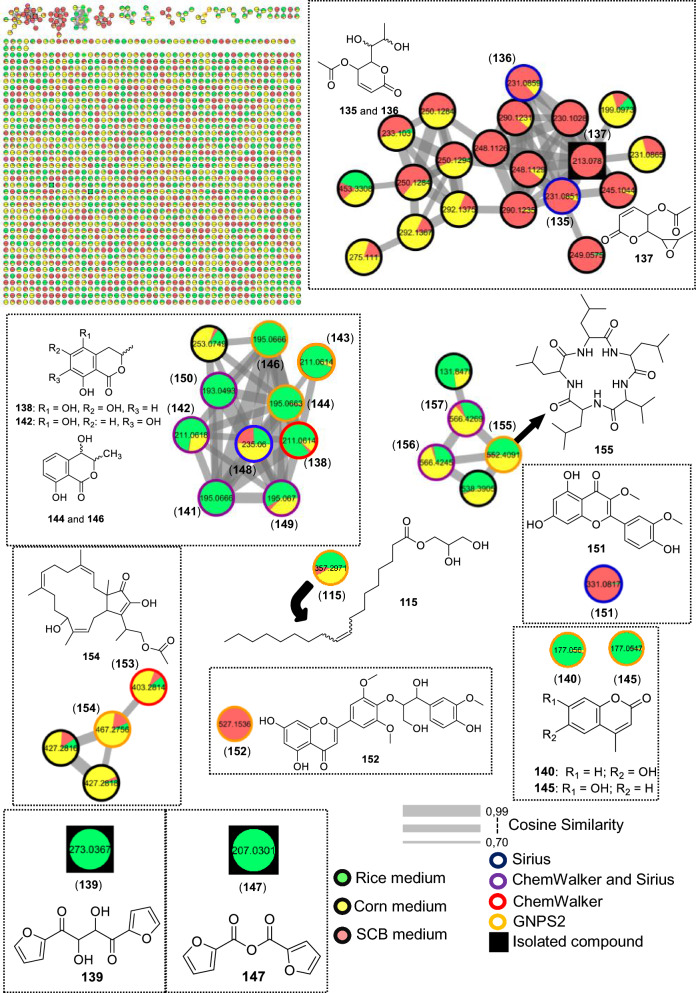
Molecular Network generated by the GNPS2 platform of metabolites produced by the LMC23006 fungus in rice and corn culture media

The main cluster in the molecular network (Fig. 18) included a compound (**137**) isolated from the SCB extract.

The ^1^H NMR spectrum of compound **137** showed methyl proton resonances with chemical shifts characteristic of an α-position to a carbonyl at *δ*_H_ 2.11 (3H, s) and another methyl group coupling with a CH at *δ*_H_ 1.25 (3H, d, *J* = 5.1 Hz). The corresponding methine proton exhibited a deshielded chemical shift consistent with oxygen attachment at *δ*_H_ 3.05 (1H, dq, *J* = 2.1, 5.1 Hz). The following proton, also with small coupling constants, appeared at *δ*_H_ 3.02 (1H, dd, *J* = 2.1, 6.3 Hz). The subsequent proton, coupling with the latter, was more deshielded at *δ*_H_ 4.35 (1H, dd, *J* = 3.2, 6.3 Hz), suggesting ester substitution. Another ester-linked proton appeared at *δ*_H_ 5.19 (1H, ddd, *J* = 0.4, 3.2, 5.4 Hz). The last two protons revealed a double bond, at *δ*_H_ 6.20 (1H, dd, *J* = 0.4, 9.8 Hz) and *δ*_H_ 7.01 (1H, dd, *J* = 5.4, 9.8 Hz). This spectroscopic pattern was consistent with a reduced six-membered lactone bearing an ester group at the lactone methine carbon and another corresponding to an acetate. To satisfy the exact mass annotated in Table S16, the formation of an epoxide between the two oxygenated protons was required, allowing the compound **137** to be assigned as asperlin [75].

In the same cluster, two diastereomeric derivatives of asperlin (**137**) were annotated by SIRIUS: asperlinol (**135**) and its stereoisomer (**136**), which exhibited distinct retention times. These three compounds were produced almost exclusively in SCB medium, with only minor amounts detected in corn, and asperlin was the major metabolite in this substrate. When the medium was switched to rice, the metabolic profile shifted significantly, with predominance of mellein and isocoumarin derivatives.

In this cluster, GNPS2 enabled the annotation of the diastereomers 4-hydroxymellein (**144**) and (**146**), as well as asperochrin F (**138**). Additional derivatives with the same skeletons were annotated by SIRIUS and ChemWalker, leading to the assignment of nine of the ten metabolites within the cluster like 7-hydroxy-4-methylcoumarin-3-acetic acid (**148**), differing in the position of hydroxyl substitutions. As mentioned previously, other metabolites sharing this scaffold but appearing as singletons included 6-hydroxy-4-methylcoumarin (**140**) and hymecromone (**145**). These compounds were preferentially produced in rice and corn media, with lower levels in SCB, highlighting the importance of substrate variation for accessing new metabolites.

A cluster of cyclic peptides was also observed, enabling the annotation of compounds (**155–157**), including luminmide B (**155**), annotated by GNPS. Another cyclic peptide annotated via GNPS2 was beauvericin (**70**). This compound, although known to be isolated from *Fusarium*, was described for the first time in this specific species. In corn medium, preferential production of sesterterpenes was observed, leading to the annotation of fusaproliferin (**154**) by GNPS2 and its derivative terpestacin (**153**) by ChemWalker. Two additional metabolites detected predominantly in SCB medium as singletons were quercetin 3,3′-dimethyl ether (**151**) and *o*-guaiacylglycerol (**152**).

Two exclusive metabolites were isolated from the rice medium extract. The first displayed only three proton signals: *δ*_H_ 7.80 (dd, *J* = 1.7, 0.8 Hz, 1H), *δ*_H_ 7.23 (dd, *J* = 3.5, 0.8 Hz, 1H), and *δ*_H_ 6.64 (dd, *J* = 3.5, 1.7 Hz, 1H). The multiplicity, coupling constants, and chemical shifts were characteristic of a furan ring. Based on the exact mass (Table S16) from HRMS analysis and data mining using the GNPS2 platform, in comparison with literature data, this metabolite was assigned as **147**, 2-furancarboxylic acid, 2,2′-anhydride.

The second compound presented a similar structure, also containing a furan ring, with an additional proton integrating for two hydrogens and a chemical shift characteristic of an oxygenated carbon. By comparison with literature and its exact mass, the compound was identified as rhizosolaniol (**139**).

#### Co-cultivation

**Fig. 19:**
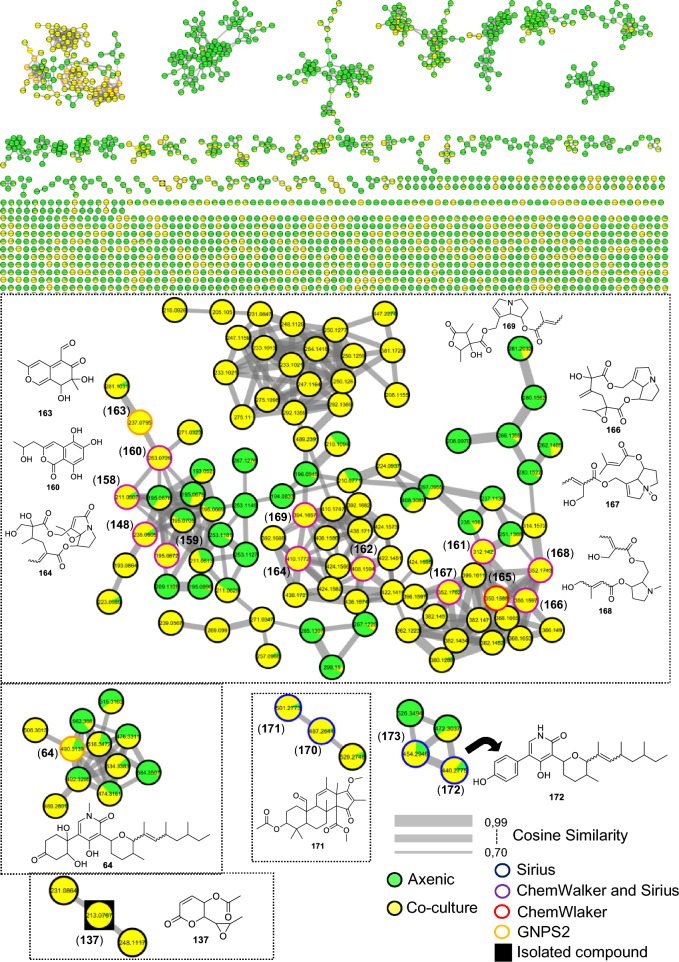
Molecular network generated by the GNPS2 platform from the LC–MS/MS data of the cocultures between *N. ribis* and six different *Fusarium* species

To investigate the metabolites produced during co-cultivation, we analyzed data from rice medium cultures of both the interaction and their respective axenic controls using the GNPS2 platform. The choice of the endophytic fungus *N. ribis* and rice medium for the experiment was based on specific methodological criteria reported in other works. *N. ribis* was selected due to its diverse metabolic profile and promising biological activity documented in previous studies, which allowed its individual cultivation to serve as a robust baseline for identifying alterations induced by the interaction. In parallel, rice medium was chosen for its proven efficacy in co-culture studies, enabling the identification of metabolic variations between axenic cultures and co-cultures by both ^1^H NMR and LC–MS [37, 41, 74, 76]. The Cytoscape network was configured to highlight metabolites from axenic cultures in green and those from co-cultivation in yellow (Fig. 19).

**Fig. 20:**
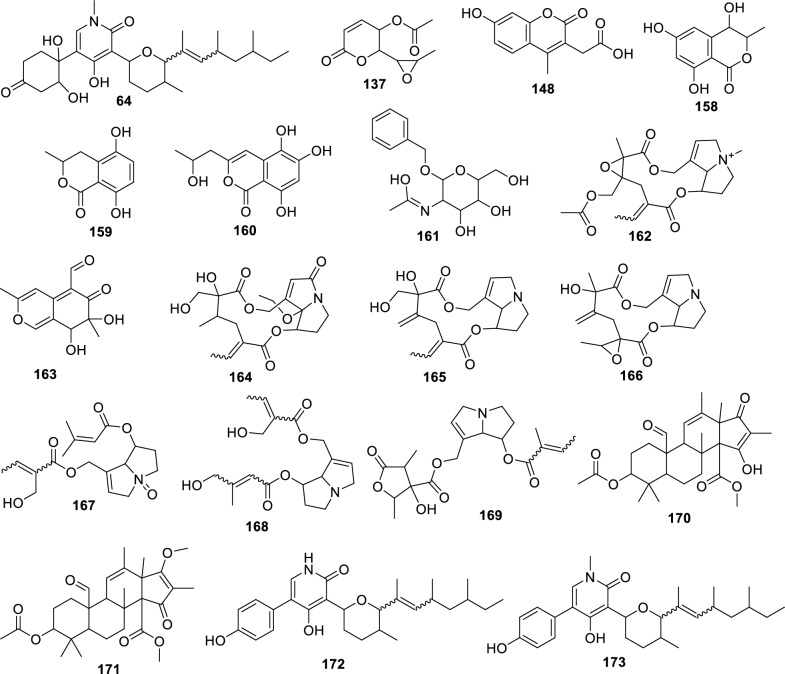
Compounds annotated by molecular networking from the cocultures of *N. ribis* with six different *Fusarium* species. Highlighted compound (**137**) was isolated and structurally characterized

This representation provided a clear visualization of metabolites that were either unique to the co-cultivation or significantly upregulated under this condition. In total, 18 metabolites were annotated (Fig. 20 and Table S17).

A main cluster was observed, containing a wide variety of metabolites, most of which were produced under co-culture conditions and only in small quantities in the axenic culture. The compound austidiol (**163**) was annotated by GNPS and was exclusive to the co-culture. Alongside it, the hydroxymelleins previously reported for LMC23006 were annotated; however, a new hydroxymellein with a different retention time of 6.51 min was annotated by SIRIUS and ChemWalker. This compound, featuring the hydroxyl group at a different position, was designated as hydroxymellein (**159**). Other derivatives such as 1H-2-benzopyran-1-one, 3,4-dihydro-4,6,8-trihydroxy-3-methyl (**158**) and peniisocoumarin G (**160**) were also annotated by SIRIUS and ChemWalker.

Another class of compounds was annotated as alkaloids with a pyrrolizidine nucleus, including latifoline (**169**), O-acetylerucifoline N-oxide (**162**), and 8-ethoxy-3-oxoretrorsine (**164**). The tree of them annotated by ChemWalker and SIRIUS and were exclusive to the co-culture conditions. Derivatives of this class, such as jacozine (**166**) and doriasenine (**168**), were also annotated. This analysis of the co-culture extracts describes only the compounds that were produced or showed increased expression under this condition. From this, 13 compounds were annotated in this cluster—a very small fraction given the number of co-culture metabolites visualized in Fig. 19—highlighting the need for compound isolation to understand the metabolic changes further.

Another cluster showed increased production of oxysporidinone (**64**) and the production of unannotated derivatives. However, the ion *m/z* 506.3013 suggests a possible oxygen addition to the structure. A different cluster allowed for the annotation of two derivatives of compound **64** that possess an aromatic ring not previously observed, as they did not cluster with compounds from the other fungi. These were annotated as andrastin A (**170**) and citreohybridone G (**171**). Analysis of metabolite intensities indicated higher production under co-culture conditions. In another cluster, two compounds were annotated by SIRIUS: andrastin A (**170**) and citreohybridone G (**171**).

A cluster for the isolated compound asperlin (**137**) was annotated in the co-culture combination, while two other compounds in the cluster could not be. This compound was produced exclusively in the LMC23006 axenic culture in SCB medium. Nevertheless, the co-culture condition enabled access to the same biosynthetic pathway for the production of asperlin on rice medium. It was observed in all co-culture combinations, indicating that the LMC23006 fungus produces this compound in significant quantities when in the presence of another fungus, potentially suggesting it is an antifungal compound. Further experiments are needed to confirm this. These results demonstrate that microbial interactions lead to competition for space and nutrients, facilitating the activation of biosynthetic pathways and access to new compounds.

### Statistical analysis of extracts

**Fig. 21:**
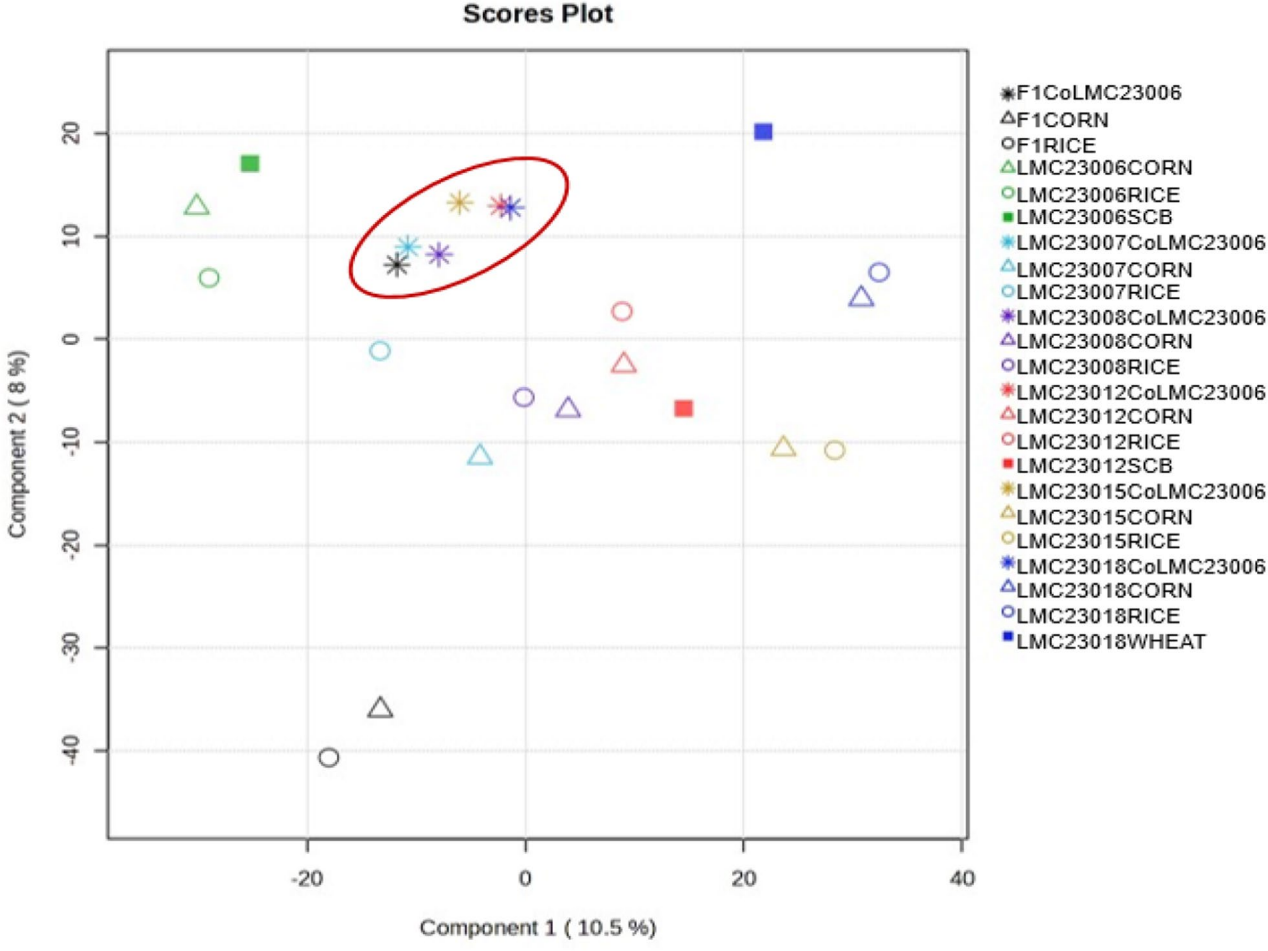
PLS-DA scores plot of *Fusarium* and *Neofusicoccum* cultures under different substrates and co-cultivation conditions. Legend: The samples highlighted in red correspond to the co-cultures of *Fusarium* with *N. ribis* (CoLMC23006) on different substrates. The PLS-DA model was validated by tenfold CV (*R*^2^ = 0.85, *Q*^2^ = 0.81) and a permutation test (*p* < 0.05)

PLS-DA analysis revealed a tendency for clustering of *Fusarium–N. ribis* co-cultures, largely independent of the substrate employed (Fig. 21, red circle). This pattern suggests that interspecific interaction exerts a stronger influence on metabolic regulation than the substrate alone, leading to greater similarity among co-culture chemical profiles compared to monocultures.

In contrast, *Fusarium* monocultures displayed wider dispersion, highlighting the contribution of substrate in shaping secondary metabolite production. It should be noted, however, that the first two PLS-DA components account for only ~ 18.5% of the variance, and thus part of the observed metabolic variability is captured in higher dimensions not represented in the score plot. The distinct separation observed for the wheat extract of strain LMC23018 (blue square) suggests a divergent metabolic response, which annotation analysis associated with the absence of fumonisin derivatives at significant levels in this condition. Overall, the results align with the GNPS2 molecular networking data, which indicated comparable metabolic outputs across co-cultures and highlighted the endophytic strain LMC23006 as a major contributor to the annotated metabolites. Taken together, these findings support the view that co-cultivation can enhance metabolic convergence and potentially activate otherwise silent biosynthetic pathways, although definitive evidence requires integration of multivariate statistics with targeted metabolite annotation.

**Fig. 22:**
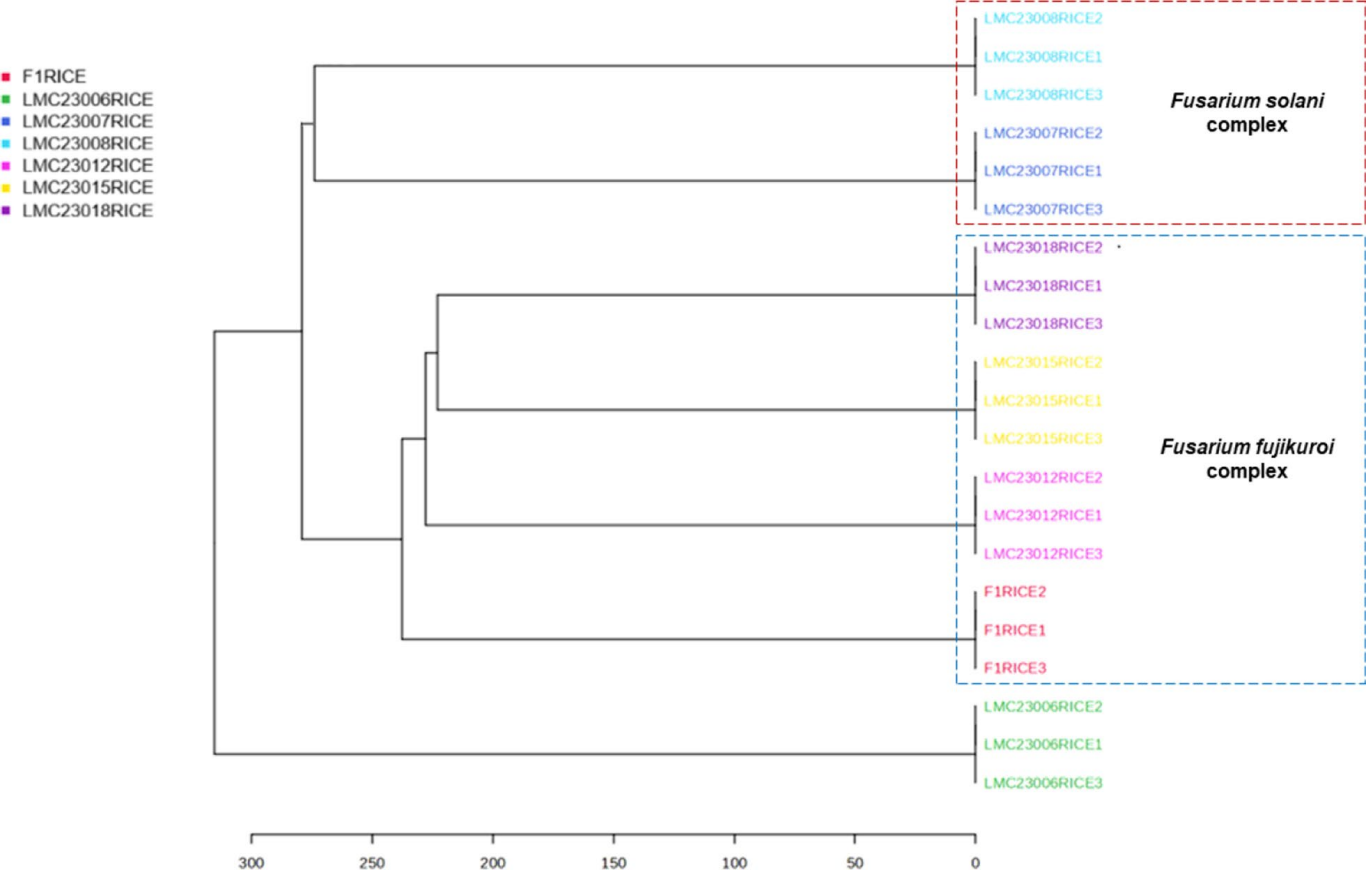
Hierarchical clustering of extracts in rice medium

Furthermore, statistical analysis of *Fusarium* species grown in rice medium revealed a noteworthy pattern in the Hierarchical Clustering Dendrogram (Fig. 22). In this dendrogram, fungal metabolites clustered in a hierarchy that closely reflected the phylogenetic tree of *Fusarium* species. Strains belonging to the *Solani* complex grouped together, as did those of the *Fujikuroi* complex, as highlighted in the Fig.. In contrast, LMC23006 (*N. ribis*) appeared as an outgroup, similar to its placement in phylogenetic analyses, being more distant and not clustering with the other strains.

This result highlights the correlation between genomics and metabolomics, suggesting that genetically related fungi share enzymes and proteins that catalyze similar biochemical reactions, leading to the production of structurally related metabolites.

All extracts obtained from axenic cultures were tested for biological activity, primarily those from the rice and corn media, and the results were published. The co-culture extracts were reported to have higher activity than those obtained from the axenic cultures [76]. The biological activity of the crude extracts from the co-culture of *Fusarium* sp. with strain LMC23006 on rice medium was evaluated against the enzymatic inhibition of papain. Table 4 shows the percentage of inhibition at a concentration of 25 µg/mL. SBio Table: Papain inhibitory activity of co-cultivation extracts.

**Table 4:** Papain inhibitory activity of the co-culture crude extracts

Strain	% inhibition at 25 µM
	Co-culture^*^
LMC23007.2	75.6
LMC23008	49.2
LMC23012	89.4
LMC23015	86.2
LMC23018	89.1
F1	76

The co-culture were with LMC23006RICE

**Fig. 23:**
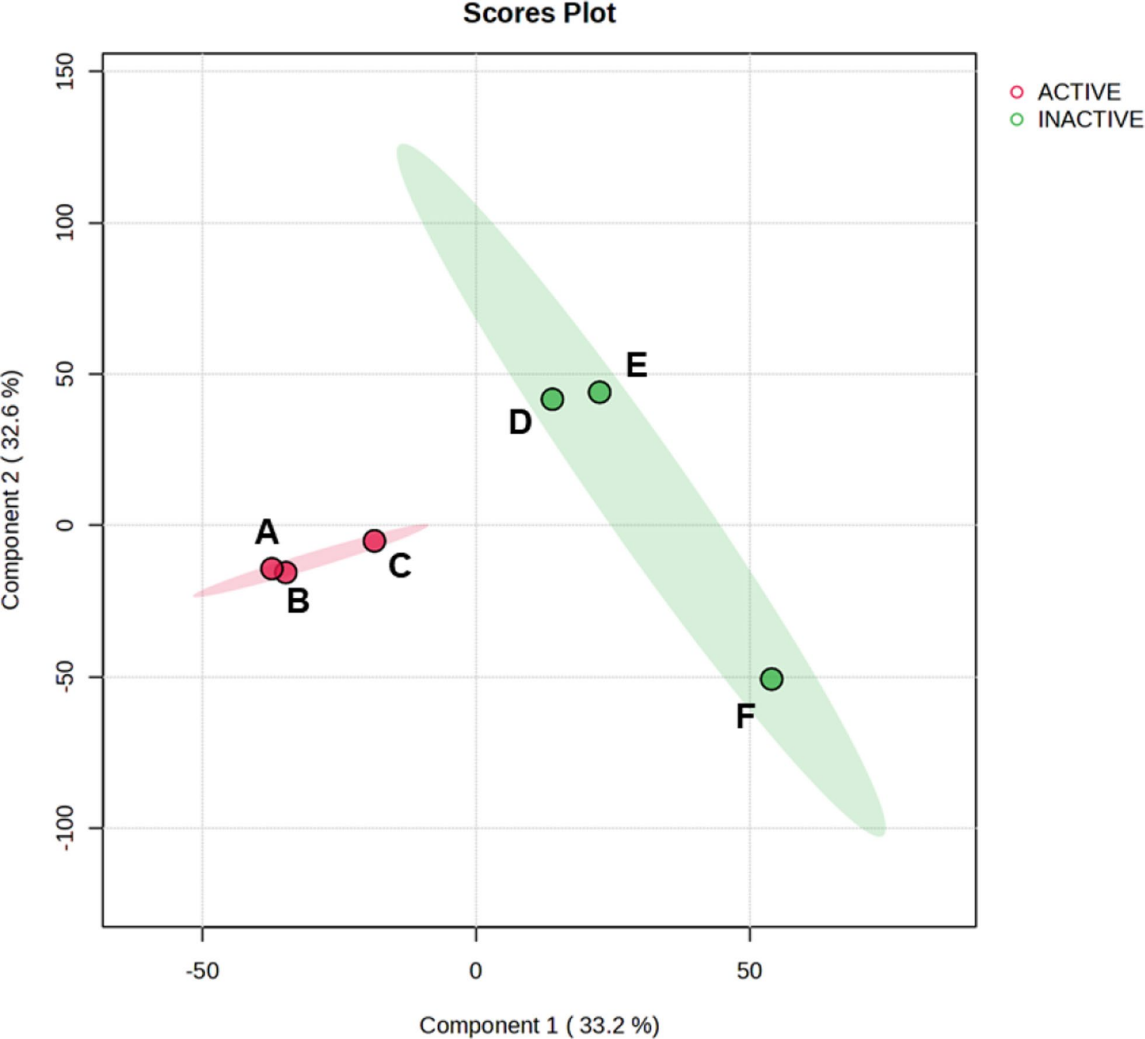
PLS-DA analysis of active coculture extracts. The Fig. shows the co-culture combinations of LMC23006 with: A) LMC23012, B) LMC23018, C) LMC23015, D) LMC23007.2, E) LMC23008, and F) F1

Significant activity can be observed, with extracts LMC23012, LMC23015, and LMC23018 being the most bioactive. PLS-DA analysis of the extracts, which considered those with inhibition greater than 80% as active, showed a clustering of these extracts, indicating that they are chemically related, as shown in Fig. 23 (A, B, and C). The remaining extracts, in turn, exhibited more distinct chemical profiles from each other.

## Methodology

### Isolation and identification of endophytic and phytopathogenic fungi from papaya and pineapple

#### Isolation

Commercial fruits of papaya (*Carica papaya*) and pineapple (*Ananas comosus*) were used in the experiments. The method for isolating endophytic fungi from fruits was adapted from previously established protocols [77–79]. Initially, healthy fruits were washed under running water to remove surface debris. Fragments measuring 3 × 3 × 6 cm were aseptically cut using a sterile scalpel. Surface disinfection was performed by sequential immersion in 2.5% sodium hypochlorite (NaOCl) for 30 s and 70% ethanol for 30 s. This procedure was repeated three times, followed by rinsing with autoclaved distilled water to remove disinfectant residues. To confirm the effectiveness of the sterilization, two controls were performed: (i) touch control, in which the fruit surface was pressed onto Potato Dextrose Agar (PDA) plates to verify the absence of epiphytic fungi; and (ii) wash control, in which 200 μL aliquots of the final rise water were plated onto PDA (three replicates) and incubated to ensure complete removal of surface contaminants.

Following surface sterilization, the fruit fragments were transferred to autoclaved glass flasks. Small incisions were made on the fruit surface with a sterile scalpel to expose inner tissues and promote fungal growth. The fragments were then incubated for 13 days to allow fungal development. These procedures were applied for the isolation of endophytic fungi from both papaya and pineapple.

For the isolation of phytopathogenic fungi, papaya and pineapple fruits showing disease symptoms were collected from the same location as the endophytic samples. The fruits were first cleaned under running water to remove surface contaminants and then placed in autoclaved 2-L beakers covered with aluminum foil. The samples were monitored until microbial growth or lesion formation was observed—fungal growth appeared on papaya after 14 days, while lesions developed on pineapple after 21 days. Affected tissue sections were excised with a sterile scalpel and subjected to the same surface sterilization protocol applied to endophytic fungi, ensuring that only internal pathogens were isolated. The same control procedures were also performed [20, 80].

After fungal growth on the fruits, isolates were transferred to PDA plates supplemented with chloramphenicol (200 μg.L⁻^1^) using the streak plate method. Following 1–3 days of incubation at 25 °C, individual colonies were subcultured until pure fungal cultures were obtained. The isolates were then characterized based on macro- and micromorphological traits, as well as by sequencing the internal transcribed spacer (ITS) region of ribosomal DNA**.**

#### Identification

Macromorphological characterization was carried out by examining colony traits, including appearance, coloration, and diameter, to group similar isolates. One representative from each group was selected for further analysis. Fungi were inoculated onto 9 × 1.5 cm Petri dishes containing autoclaved PDA medium by transferring small mycelia fragments with a sterile incubation loop. The plates were incubated at 37 °C for 7 days.

Micromorphological characterization was performed using microcultures of fungal conidia on a thin (5 mm) PDA layer placed on sterilized glass slides. A sterile coverslip was placed over the conidial inoculum, and the preparations were incubated in a humid chamber at 37 °C for 24 h or until the formation of asexual reproductive structures. The humid chamber consisted of a Petri dish containing moistened filter paper, a V-shaped glass rod, and the prepared slide. After incubation, coverslips were removed and mounted on glass slides with a drop of lactophenol cotton blue. Microconidia, macroconidia, conidiophores, and hyphae were examined under an Observer Z1 optical microscope (Carl Zeiss Jena, Germany) at 400 × magnification. Images were captured using an HDCE-X5 camera and analyzed using ScopImage 9.0 software. Morphological identification was used to assign isolates to the genus-level, guiding subsequent molecular identification to determine species-level identity [81].

Genomic DNA extraction from fungal isolates was performed using a modified method [82]. Fungal cultures were grown in PDB medium at 37 °C and 120 rpm (MAX Q 4000, Thermo Fisher Scientific) for 48 h. Mycelia were vacuum-filtered through quantitative filter paper and flash-frozen in liquid nitrogen. Genomic DNA was exposed by mechanical disruption of the frozen mycelium using a sterile mortar and pestle in the presence of liquid nitrogen. Subsequently, 500 μL of DNA extraction buffer (2% Triton X-100 (w/v), 1% SDS (w/v), 1 mM sodium chloride, 75 mM EDTA, Tris–HCl pH 8.0, and ultrapure water) was added to the ground mycelium, followed by phenol:chloroform (1:1 v/v) extraction. The mixture was homogenized for 10 min using a tube shaker and centrifuged at 12,000 × *g* for 15 min. The supernatant was transferred to a new tube, mixed with chloroform, homogenized, and centrifuged again at 12,000 × *g* for 5 min. DNA was precipitated from the resulting supernatant with isopropanol and recovered by centrifugation at 12,000 × *g* for 1 min. The pellet was washed with 70% ethanol, air-dried, and the DNA was eluted in ultrapure water. The DNA was treated with Rnase (300 ng/mL) at 37 °C for 1 h. DNA concentration and purity were determined using a spectrophotometer [83] and integrity was assessed by 1% agarose gel electrophoresis stained with SYBR Safe (Thermo Fisher, Massachusetts, USA). The extracted DNA was used for subsequent molecular identification of the isolates at the species level.

Polymerase chain reaction (PCR) was performed using Phusion^®^ High-Fidelity DNA Polymerase (New England BioLabs, Inc.) with specific primers for the internal transcribed spacer (*ITS*) region of ribosomal DNA (*ITS*1, 5′-TCCGTAGGTGAACCTGCGG-3′, and *ITS*4, 5′-TCCTCCGCTTATTGATATGC-3′)[84], as well as for the tubulin (*Bt2*a 5′GGTAACCAAATCGGTGCTGCTTTC3′ foward, and *Bt2*b 5′ACCCTCAGTGTAGTGACCCTTGGC3′ reverse,)[85], the second largest subunit of the DNA-dependent RNA polymerase II (*RPB2*; *RPB2* forward, 5′-GGGGWGAYCAGAAGAAGGC-3′, and *RPB2* reverse, 5′-CCCATRGCTTGYTTRCCCAT-3′) [86], and calmodulin (*cmd*; cmd5 5′CCGAGTACAAGGAGGCCTTC3′ forward and cmd6 5′ CCGATAGAGGTCATAACGTGG reverse) [87]. PCR products were purified using the Wizard^®^ SV Gel and PCR Clean-Up System (Promega) and subsequently sequenced on an ABI3730 DNA Analyzer (Applied Biosystems), targeting the *ITS* region as well as the *RPB2, TUB2,* and *cmd* loci (species-specific variations are shown in Table 1). Sequence chromatograms were analyzed with ChromasPro^®^ software v1.7.6 (Technelysium Pty Ltd., Tewantin, QLD, Australia). Sequence validation was performed by comparison with publicly available DNA sequences in the National Center for Biotechnology Information (NCBI) database using the Basic Local Alignment Search Tool nucleotide (BLASTn) [88] Fungal sequences were specifically analyzed with BLASTn under an E-value threshold of 0.0, taking into account query coverage and percentage identity. All fungal sequences obtained in this study were deposited in GenBank under their respective accession numbers (Table 1).

### Phylogenetic tree construction

Phylogenetic reconstruction was performed with a combined dataset of *ITS*, *RPB2*, *TUB2,* and *cmd* marker sequences, selected according to the taxonomic classification of the studied fungi. Reference sequences were retrieved from GenBank (NCBI) database, focusing on taxonomically relevant homologs (Tables S1-S8).

Multiple sequence alignments were generated in MEGA v11 and subsequently refined manually. Evolutionary analyses were conducted employing two complementary approaches: Maximum Likelihood (ML) and Bayesian Inference (BI). For ML analysis, the Tamura-Nei molecular model of nucleotide substitution was applied with uniform rates across sites. Tree topology robustness was evaluated with 1000 bootstrap replicates. Initial trees were generated automatically by the NJ/BioNJ method and optimized through the Nearest-Neighbor-Interchange (NNI) algorithm, with parallel computation distributed across eight threads for efficiency.

Bayesian analysis was performed in MrBayes version 3.2.6 with four Markov chain Monte Carlo (MCMC) chains run for 10 million generations, sampling every 100 generations. Convergence was considered achieved when the average standard deviation of split frequencies dropped below 0.01. A 25% burn-in was applied before consensus tree calculation, and nodal support was expressed as posterior probabilities.

### Fungal cultivation, extraction, and extract selection

Different fungal strains were cultivated, including the endophytic fungus *N. ribis* (LMC23006) and the phytopathogenic fungi *F. falciforme* (LMC23007.2), *F. petroliphilum* (LMC23008), *F. sacchari* isolated from papaya (LMC23012), *F. sacchari* isolated from pineapple (LMC23015), *F. verticillioides* (LMC23018), and *F. guttiforme* (F1-MMBF 04/07). Axenic cultures were established on solid substrates, namely rice, wheat, corn, and SCB, by inoculating six 0.5 cm mycelial discs per culture. Incubations were carried out in the dark at 25 °C for 21–28 days. For medium preparation, 90 g of rice with 90 mL of distilled water, 90 g of wheat with 110 mL of distilled water, and 45 g of SCB medium, 45 g with 100 mL of distilled water were used. A co-culture assay was also conducted on rice medium, combining *Fusarium* strains and *N. ribis*. For this, six mycelial discs of each fungus were transferred to 500 mL Erlenmeyer flasks containing 90 g of rice and 90 mL of distilled water, in triplicate, and incubated for 21 days under the same conditions. After cultivation, metabolites were extracted with ethyl acetate in three sequential steps (150 mL each), consisting of maceration and ultrasonication for 5 min per cycle. Crude extracts were obtained after solvent evaporation in a rotary evaporator. All extracts obtained from axenic cultures were tested for biological activity, primarily those from the rice and corn media, and the results were published. The co-culture extracts were reported to have higher activity than those obtained from the axenic cultures [76].

From the total extracts obtained, those derived from co-cultures, as well as all axenic extracts from corn and rice substrates, were selected for GNPS2 analysis. In addition, extracts previously identified as promising in earlier studies were included, specifically, *N. ribis* (LMC23006) grown on SCB, *F. sacchari* (LMC23012) also grown on SCB, and *F. verticillioides* (LMC23018) grown on wheat.

### ^1^H NMR analysis

For the characterization of isolated compounds, one-dimensional (^1^H) and two-dimensional (HMBC and HSQC) nuclear magnetic resonance (NMR) analyses were conducted using a Bruker DRX500 spectrometer operating at 500 MHz in the Departamento de Química da Faculdade de Filosofia Ciências e Letras de Ribeirão Preto. Samples were prepared using deuterated solvents, including acetone-*d*₆, methanol-*d*₄, DMSO-*d*₆, and D₂O.

### Liquid chromatograph and isolation of compounds

Mass spectrometric data were acquired using an LC-HRMS micrOTOF Q II system (Bruker) operating in both positive and negative electrospray ionization (ESI) modes. The time-of-flight (TOF) mass analyzer was calibrated to detect ions in the *m/z* 100–1000 range. Ionization conditions were optimized with: capillary voltage at 3.5 kV, end plate offset at 500 V, drying gas (N₂) flow at 10.0 L/min (300 °C), and nebulizer pressure at 50.0 psi. Fragmentation was performed in Auto MS(n) mode, automatically selecting the three most intense ions from each MS^1^ scan for MS^2^ spectra acquisition. Chromatographic separation was performed at the Laboratório de Química Orgânica (FCFRP-USP) using a Kinetex^®^ XB-C18 column (100 × 2.1 mm, 2.6 µm—Phenomenex). The mobile phase consisted of water (A) and methanol (B), both containing 0.1% formic acid, with the following gradient: 10% B (0–1 min), linear increase to 100% B over 23 min, held for 2 min, returned to 10% B in 1 min, and re-equilibrated for 4 min (total run time: 30 min) at 0.3 mL/min flow rate. The extracts were separated and compounds obtained by high-performance liquid chromatography (HPLC–DAD) using a Shimadzu LC-6AD system with a semi-preparative Luna C-18 column (25 cm × 10 mm, 5 µm—Phenomenex). The same mobile phase conditions as the analytical method were maintained, adjusting the flow rate to 4.0 mL/min. For purification, 400 µL samples at 60 mg/mL concentration were injected. The data quality was ensured by including solvent blanks (methanol/water) at the beginning, after every four samples, and at the end of the analytical sequence to monitor system stability. Additionally, culture medium samples (matrix blanks) were analyzed alongside the fungal extracts. These controls allowed for the identification of interferents and the subtraction of compounds originating from the medium or from column and solvent contaminations during processing in MZmine. This ensured that the described metabolic profiles are genuinely produced by the fungi under the different experimental conditions.

*7-Hydroxy-3-(2-hydroxy-propyl)-5-methyl-epiisochromen-1-one* (**37**).

^1^H NMR (Acetone-*d*₆, 500 MHz): *δ*_H_ 5.97 (1H, s), 6.67 (1H, d, *J* = 2.1 Hz), 6.69 (1H, d, *J* = 2.1 Hz), 2.62 (1H, dd, *J* = 7.8, 14.2 Hz), 2.67 (1H, dd, *J* = 4.9, 14.2 Hz), 4.21 (1H, m), 1.25 (3H, d, *J* = 6.7 Hz), 2.70 (3H, s) [53].

*Fusarubin* (**38**).

^1^H NMR (DMSO-*d*₆, 500 MHz): *δ*_H_ 1.48 (3H, s), 2.59 (1H, m), 2.77 (1H, dd, *J* = 17.9, 1.6 Hz), 4.66 (1H, brd, *J* = 17.6 Hz), 4.72 (1H, d, *J* = 17.6 Hz), 6.16 (1H, d, *J* = 1.5 Hz), 6.46 (1H, s), 12.51 (1H, s), 13.00 (1H, s) [53].

*3-O-methylfusarubin* (**49**).

^1^H NMR (Acetone-*d*₆, 500 MHz): *δ*_H_ 1.47 (3H, s), 2.67 (1H, brd, *J* = 18.0 Hz), 2.84 (1H, brd, *J* = 18.0 Hz), 3.91 (3H, s), 4.44 (1H, dt, *J* = 17.8, 2.4 Hz), 4.78 (1H, d, *J* = 17.8 Hz), 6.45 (1H, s), 3.20 (3H, s) [53].

*Dihydrofusarubin* (**35**).

^1^H NMR (DMSO-*d*₆, 500 MHz): *δ*_H_ 12.26 (1H, s), 12.00 (1H, s), 6.82 (1H, s), 4.11 (1H, dd, *J* = 11.4, 4.7 Hz), 3.75 (1H, dd, *J* = 11.1, 10.9 Hz), 3.98 (3H, s), 3.22 (1H, m), 3.50 (1H, ddd, *J* = 16.5, 12.8, 3.7 Hz), 2.30 (1H, dd, *J* = 13.6, 3.6 Hz), 1.70 (1H, dd, *J* = 13.6, 11.4 Hz), 1.35 (3H, s) [54].

*5,7,10-Trihydroxy-3-methylbenz[g]isoquinoline-6,9-dione* (**40**).

^1^H NMR (DMSO-*d*₆, 600 MHz): *δ*_H_ 9.29 (1H, s), 2.66 (3H, s), 7.89 (1H, s), 5.57 (1H, s).

*Bostrycoidin* (**43**).

^1^H NMR (Acetone-*d*₆, 500 MHz): *δ*_H_ 9.38 (1H, s), 2.76 (3H, s), 8.00 (1H, s), 13.51 (1H, s), 4.07 (3H, s), 6.97 (1H, s), 13.10 (1H, s) [53].

*5-Deoxybostrycoidin* (**50**).

^1^H NMR (MeOD, 500 MHz): *δ*_H_ 9.31 (1H, s), 2.74 (3H, s), 7.96 (1H, s), 7.34 (1H, d, *J* = 2.5 Hz), 3.95 (3H, s), 6.86 (1H, d, *J* = 2.5 Hz) [55].

*7-but-15-enyl-6,8-dihydroxy-3(R)-penta-9,11-dienylisocoumarin* (**57**).

^1^H NMR (Acetone-*d*₆, 500 MHz): *δ*_H_ 5.10 (1H, brdd, *J* = 6.3, 12.2 Hz), 2.88 (1H, m), 2.97 (1H, m), 6.37 (1H, s), 11.55 (1H, s), 5.47 (1H, dd, *J* = 6.3, 15.7 Hz), 6.12 (1H, dd, *J* = 10.4, 15.7 Hz), 6.40 (1H, dd, *J* = 10.4, 15.3 Hz), 5.83 (1H, dq, *J* = 6.8, 15.3 Hz), 1.75 (3H, d, *J* = 6.8 Hz), 3.29 (2H, d, *J* = 6.3 Hz), 5.55 (1H, dq, *J* = 1.4, 6.3, 15.3 Hz), 5.73 (1H, dd, *J* = 6.5, 15.3 Hz), 1.57 (3H, d, *J* = 6.4, 1.4 Hz) [61].

*7-but-2-enyl-6,8-dihydroxy-3-pent-3-enyl-3,4-dihydroisochromen-1-one* (**58**).

^1^H NMR (Acetone-*d*₆, 500 MHz): *δ*_H_ 4.49 (1H, dddd, *J* = 4.1, 5.2, 7.7, 11.0 Hz), 2.83–2.91 (2H, m), 6.30 (1H, s), 11.51 (1H, s), 1.81 (1H, dddd, *J* = 5.9, 7.9, 8.7, 14.1 Hz), 1.70 (1H, dddd, *J* = 5.1, 6.6, 9.3, 14.1 Hz), 2.07–2.20 (2H, m), 5.31–5.52 (4H, m, overlapped), 5.31–5.52 (4H, m, overlapped), 1.52 (3H, dq, *J* = 1.4, 6.3 Hz), 3.22 (2H, brd, *J* = 6.3 Hz), 5.31–5.52 (4H, m, overlapped), 5.31–5.52 (4H, m, overlapped), 1.56 (3H, dd, *J* = 1.2, 5.1 Hz) [62].

*7-Butyl-6,8-dihydroxy-3(R)-pent-11-enylisochroman-1-one* (**59**).

^1^H NMR (Acetone-*d*₆, 500 MHz): *δ*_H_ 4.54 (1H, dddd, *J* = 4.6, 8.5, 8.6, 11.5 Hz), 2.90 (2H, m), 6.34 (1H, s), 11.58 (1H, s), 1.86 (1H, dddd, *J* = 6.2, 8.5, 11.5, 14.3 Hz), 1.76 (1H, dddd, *J* = 4.6, 6.6, 6.7, 14.3 Hz), 2.20 (2H, m), 5.49 (1H, m, overlapped), 5.51 (1H, m, overlapped), 1.62 (3H, brd, *J* = 4.9 Hz), 2.62 (2H, brt, *J* = 7.5 Hz), 1.51 (2H, quint, *J* = 7.5 Hz), 1.36 (2H, sext, *J* = 7.4 Hz), 0.91 (3H, t, *J* = 7.4 Hz) [62].

*10-Hydroxyfusaric acid* (**73**).

^1^H NMR (CDCl₃, 500 MHz): *δ*_H_ 8.12 (1H, d, *J* = 7.9 Hz), 7.92 (1H, dd, *J* = 7.9, 1.9 Hz), 8.45 (1H, s), 2.73 (2H, t, *J* = 6.6 Hz), 1.67 (4H, m), 4.04 (2H, t, *J* = 6.0 Hz).

*5-(3,4-Dihydroxybutyl)-2-pyridinecarboxylic acid* (**72**).

^1^H NMR (MeOD, 500 MHz): *δ*_H_ 8.10 (1H, d, *J* = 8.0 Hz), 7.92 (1H, dd, *J* = 8.0, 2.0 Hz), 8.54 (1H, d, *J* = 1.7 Hz), 2.94 (1H, ddd, *J* = 14.2, 10.1, 4.9 Hz), 2.82 (1H, ddd, *J* = 14.2, 9.5, 7.0 Hz), 1.95 (1H, dddd, *J* = 13.7, 10.1, 7.0, 3.6 Hz), 1.80 (1H, dddd, *J* = 13.7, 9.5, 5.9, 4.9 Hz), 3.74 (1H, m), 3.52 (1H, dd, *J* = 11.2, 5.8 Hz), 3.57 (1H, dd, *J* = 11.2, 4.8 Hz) [63].

*2-Pyridinecarboxylic acid, 5-[3-(acetyloxy)butyl]* (**74**).

^1^H NMR (Acetone-*d*₆, 500 MHz): *δ*_H_ 8.08 (1H, d, *J* = 8.0 Hz), 7.92 (1H, dd, *J* = 8.0, 2.0 Hz), 8.57 (1H, d, *J* = 2.0 Hz), 2.77–2.99 (2H, m), 1.90–1.96 (2H, m), 4.88 (1H, dq, *J* = 12.5, 6.3 Hz), 1.23 (3H, t, *J* = 6.3 Hz), 1.98 (3H, s).

*6-hydroxy-6-(4-methoxy-2-oxo-2H-pyran-6-yl)-2,4-dimethylhexanoic acid* (**81**).

^1^H NMR: *δ*_H_ 6.06 (1H, d, *J* = 2.0 Hz, H4), 5.39 (1H, d, *J* = 2.0 Hz, H6), 4.40 (1H, dd, *J* = 10.1, 2.9 Hz, H7), 1.57 (1H, m, H8a), 1.76 (1H, m, H8b), 1.79 (1H, m, H9), 1.32 (1H, m, H10a), 1.68 (1H, m, H10b), 2.54 (2H, td, *J* = 13.7, 6.9 Hz, H11), 3.77 (3H, s, H13), 0.93 (3H, d, *J* = 6.5 Hz, H14), 1.13 (3H, d, *J* = 6.9 Hz, H15).

^13^C NMR: *δ*_C_ 164.48 (C1), 166.16 (C3), 98.96 (C4, HMBC: 170.12; 166.16; 68.65; 88.11), 170.12 (C5), 88.11 (C6, HMBC: 170.12; 164.48; 98.96), 68.65 (C7, HMBC: 166.16; 98.96; 41.82; 27.16), 41.82 (C8, HMBC: 166.16; 68.65; 41.08; 27.16; 19.42), 27.16 (C9), 41.08 (C10, HMBC: 180.16; 41.82; 36.26; 27.16; 19.42; 17.42), 36.26 (C11, HMBC: 180.16; 41.08; 27.16; 17.13), 180.16 (C12), 56.16 (C13), 19.42 (C14), 17.13 (C15).

*Asperlin* (**137**).

^1^H NMR (Acetone-*d*₆, 500 MHz): *δ*_H_ 1.25 (3H, d, *J* = 5.1 Hz), 2.11 (3H, s), 3.02 (1H, dd, *J* = 2.1, 6.3 Hz), 3.05 (1H, dq, *J* = 2.1, 5.1 Hz), 4.35 (1H, dd, *J* = 3.2, 6.3 Hz), 5.19 (1H, ddd, *J* = 0.4, 3.2, 5.4 Hz), 6.20 (1H, dd, *J* = 0.4, 9.8 Hz), 7.01 (1H, dd, *J* = 5.4, 9.8 Hz) [75].

*Furan-2-carboxylic anhydride* (**147**).

^1^H NMR (Acetone-*d*₆, 500 MHz): *δ*_H_ 7.80 (1H, dd, *J* = 1.7, 0.8 Hz), 7.23 (1H, dd, *J* = 3.5, 0.8 Hz), 6.64 (1H, dd, *J* = 3.5, 1.7 Hz) [89].

*Rhizosolaniol* (**139**).

^1^H NMR (Acetone-*d*₆, 500 MHz): *δ*_H_ 8.00 (1H, d, *J* = 1.6 Hz), 7.60 (1H, d, *J* = 3.7 Hz), 6.78 (1H, dd, *J* = 3.7, 1.6 Hz), 5.39 (1H, s) [90].

### Data processing and molecular networking

The raw data were converted to the.mzXML open format using Data Analyses v4.3 software (Bruker Daltonics, Germany). The.mzXML files were pre-processed in MZmine version 4.7.8 (BMC Bioinformatics, UK). Mass detection was performed in centroid mode, with a noise level of 2000.0 for MS1 spectra and 200.0 for MS2 spectra, covering a retention time range of 0.01 to 30.14 min. Chromatogram construction was carried out using the ADAP algorithm, with a minimum scan group size of 5, an intensity threshold of 3500.0, and an *m/z* tolerance of 0.01 or 12.0 ppm. Chromatographic deconvolution was performed with the Minimum Search algorithm, applying a minimum relative height of 0.25, an absolute height of 5000.0, and a peak duration range of 0.0 to 1.0 min. Isotopic peaks were grouped with an *m/z* tolerance of 0.01 or 8.0 ppm, a retention time tolerance of 0.11 min, a maximum charge of 2, and the most intense isotope set as the representative. Peak alignment was conducted using the Join Aligner method, with an *m/z* tolerance of 0.01 or 12.0 ppm, a weighting factor of 3.0 for *m/z* and 1.0 for retention time, and an RT tolerance of 0.11 min. After blank subtraction (minimum detection of 1 and MAXIMUM ratio criteria), the data were filtered to remove irrelevant features. A feature quantitative table (.csv) and summarized MS/MS spectra (.mgf) were exported for GNPS2 and analysis [91, 92].

Mass spectrometry data were processed on the GNPS2 platform (version 2025.06.17, Wang Bioinformatics Group, USA, 2025) using Feature-Based Molecular Networking (FBMN) workflow. The parameters used were fragment mass tolerance of 0.05 Da, precursor mass tolerance of 0.02 Da, a similarity threshold (cosine score) of 0.70, and a minimum of 5 matched peaks for networking construction. Spectral annotations were generated by matching against GNPS2 libraries, applying a minimum cosine score of 0.70 and at least 4 matched peaks for validation. For spectra not annotated by direct spectral matching, a tiered annotation strategy was implemented. First, ChemWalker was used via the GNPS2 interface to propagate annotations, considering the [M + H]⁺ adduct as primary, with a mass tolerance of 15 ppm and a maximum of 4 propagated compounds. Remaining unannotated features were subjected to in silico prediction using SIRIUS (version 6.2.2, Böcker, Germany, 2025) for molecular formula and structural elucidation, including [M + H]⁺, [M + Na]⁺ adducts with the same 15 ppm tolerance. Final annotations were selected based on software rankings, consistency with ChemWalker predictions, and cross-referencing with fungal metabolite literature [91–94]. Molecular networks were visualized and analyzed in Cytoscape (version 3.10.3, Cytoscape Team, USA, 2025), enabling systematic exploration of metabolite relationships.

The processing and statistical analysis of metabolomic data were conducted on the MetaboAnalyst 6.0 platform (https://www.metaboanalyst.ca, accessed on August 26, 2025). The raw data, containing masses and intensities, were submitted to preprocessing. Initially, a low repeatability filter (RSD > 20% in QC samples) was applied, followed by a low variance filter (40% cutoff) to ensure data quality. The samples were then normalized by the median and subjected to auto scaling. For multivariate analysis, Partial Least Squares Discriminant Analysis (PLS-DA) was employed to discriminate between the experimental groups. The clustering structure of the data was further visualized using a dendrogram.

Papain, a cysteine protease frequently applied as a model enzyme in inhibitor screening studies, was employed to evaluate the inhibitory potential of fungal extracts obtained from rice-based cocultures. Enzymatic activity was assessed by monitoring the fluorescence released upon hydrolysis of the synthetic substrate Z-Phe-Arg-MCA (ZFR-MCA), which liberates 7-amino-4-methylcoumarin (MCA).

### Papain inhibition assay

Each reaction (final volume of 200 µL) contained 5 µL papain solution (80 nM), 2 µL dithiothreitol (500 mM), and 158 µL sodium acetate buffer (100 mM, pH 5.5, supplemented with 5 mM EDTA). After a pre-incubation period of 5 min at 27 °C, 5 µL of fungal extract (dissolved in DMSO) or DMSO alone (control) was added. Extracts were tested at a final concentration of 25 µg/mL. After an additional 5-min equilibration, 30 µL ZFR-MCA (0.6 mM) was introduced, and fluorescence was recorded at 380/460 nm in 5-min intervals using a microplate reader (SpectraMax M3, Molecular Devices) [74].

To validate the assay, papain activity was titrated with E-64 (range 1.0 µM–31.25 nM), an established cysteine protease inhibitor, which served as the positive control. All measurements were performed in triplicate within two independent experiments (n = 6), ensuring reproducibility of the results.

## Conclusion

This study highlights the vast chemical diversity of fruit-associated fungi and demonstrates that their metabolic expression is driven by a complex interplay between phylogenetic identity and environmental stimuli. By integrating metabolomics with ecological strategies like co-cultivation, we showed that silent biosynthetic pathways can be effectively triggered to produce bioactive molecules, such as papain protease inhibitors. Beyond identifying 173 compounds, our findings establish a robust framework for bioprospecting, suggesting that fungal specialized metabolism is best explored through multidimensional approaches. These results provide a foundation for future research into targeted natural product discovery and the development of new biotechnological applications derived from both phytopathogenic and endophytic fungal strains.

## Supplementary Information

Supplementary Material 1.

## Data Availability

All the relevant data are within the manuscript.
