# Supplementary material for: GNPS2 molecular networking reveals metabolic diversity in fungi isolated from protease-rich fruits
Source: Nat Prod Bioprospect. 2026 Apr 7;16(1):50. doi: 10.1007/s13659-026-00615-1 (PMC13057095; doi:10.1007/s13659-026-00615-1)
Supplement: Supplementary file 1 — Supplementary Material 1. [file 13659_2026_615_MOESM1_ESM.docx]

**Supplementary Information**

**GNPS2 Molecular Networking Reveals Metabolic Diversity in Fungi Isolated from Protease-Rich Fruits**

**Vitor de Souza Mazucato,^1^ Winner Duque Rodrigues,^1^ Ludmilla Tonani,^2^ Marcia Regina von Zeska Kress,^2^ Paulo Cezar Vieira ^1^***

**Affiliation**

^1^ Department of BioMolecular Sciences, Ribeirão Preto School of Pharmaceutical Sciences, University of São Paulo, Ribeirão Preto, SP, Brazil.

^2^ Department of Clinical Analysis, Toxicology, and Food Science, Ribeirão Preto School of Pharmaceutical Sciences, University of São Paulo, Ribeirão Preto, SP, Brazil

[**Fig. S1**. Photos of the fungal plates growing on PDA. 3](#_Toc208482637)

[**Fig. S2.** Morphology of fungi isolated from papaya and pineapple observed under the microscope. 4](#_Toc208482638)

[**Table S1.** Fungi and strain codes used for the phylogenetic tree construction of *Neofusicoccum* sp. 4](#_Toc208482639)

[**Table S2.** Fungi and strain codes used for the phylogenetic tree construction of Solani Complex**.** 5](#_Toc208482640)

[**Table S3.** Fungi and strain codes used for the phylogenetic tree construction of Fujikuroi Complex. 6](#_Toc208482641)

[**Table S4.** Fungi and strain codes used for the phylogenetic tree construction of *Gilbertella* sp. 7](#_Toc208482642)

[**Table S5.** Fungi and strain codes used for the phylogenetic tree construction of *Mucor* sp. 7](#_Toc208482643)

[**Table S6.** Fungi and strain codes used for the phylogenetic tree construction of Flavus Complex. 8](#_Toc208482644)

[**Table S7.** Fungi and strain codes used for the phylogenetic tree construction of *Talaromyces* sp. 9](#_Toc208482645)

[**Table S8.** Fungi and strain codes used for the phylogenetic tree construction of Terreus Complex. 9](#_Toc208482646)

[**Fig. S3.** Phylogenetic tree of the LMC23007.1 fungus**.** 10](#_Toc208482647)

[**Fig. S4.** Phylogenetic tree of the LMC23009 fungus. 11](#_Toc208482648)

[**Fig. S5.** Phylogenetic tree of the LMC23011 fungus. 12](#_Toc208482649)

[**Fig. S6.** Phylogenetic tree of the LMC23014 fungus. 12](#_Toc208482650)

[**Fig. S7**. Phylogenetic tree of the LMC23020 fungus. 13](#_Toc208482651)

[**Table S9**. The GNPS2 workflow establishes connections between fungi, their growth conditions, and the resulting annotated compounds. 13](#_Toc208482652)

[**Table S10.** Annotated compounds from the molecular networks and those isolated from the LMC23007.2 fungus. 17](#_Toc208482653)

[**Table S11.** Annotated compounds from the molecular networks and those isolated from the LMC23008 fungus 20](#_Toc208482654)

[**Table S12.** Annotated compounds from the molecular networks and those isolated from the LMC23012 fungus. 21](#_Toc208482655)

[**Fig. S8.** ¹H NMR data for compound **57** in acetone-*d*₆ (500 MHz). 24](#_Toc208482656)

[**Fig. S9.** ¹H NMR data for compound **58** in acetone-*d*₆ (500 MHz). 24](#_Toc208482657)

[**Fig. S10.** ¹H NMR data for compound **59** in acetone-*d*₆ (500 MHz). 25](#_Toc208482658)

[**Table S13.** Annotated compounds from the molecular networks and those isolated from the LMC23015 fungus. 26](#_Toc208482659)

[**Table S14.** Annotated compounds from the molecular networks and those isolated from the LMC23018 fungus. 28](#_Toc208482660)

[**Fig. S11.** ¹H NMR data for compound **81** in CDCl_3_ (500 MHz). 31](#_Toc208482661)

[**Fig. S12.** HMBC correlations for compound **81** in CDCl₃ (500 MHz). 31](#_Toc208482662)

[**Fig. S13.** HSQC spectrum of compound **81** in CDCl₃ (500 MHz). 32](#_Toc208482663)

[**Table S15.** Annotated compounds from the molecular networks and those isolated from the F1 fungus 32](#_Toc208482664)

[**Table S16.** Annotated compounds from the molecular networks and those isolated from the LMC23006 fungus 35](#_Toc208482665)

[**Table S17.** Annotated compounds from the molecular networks and those isolated from the coculture. 37](#_Toc208482666)

[**REFERENCES** 39](#_Toc208482667)


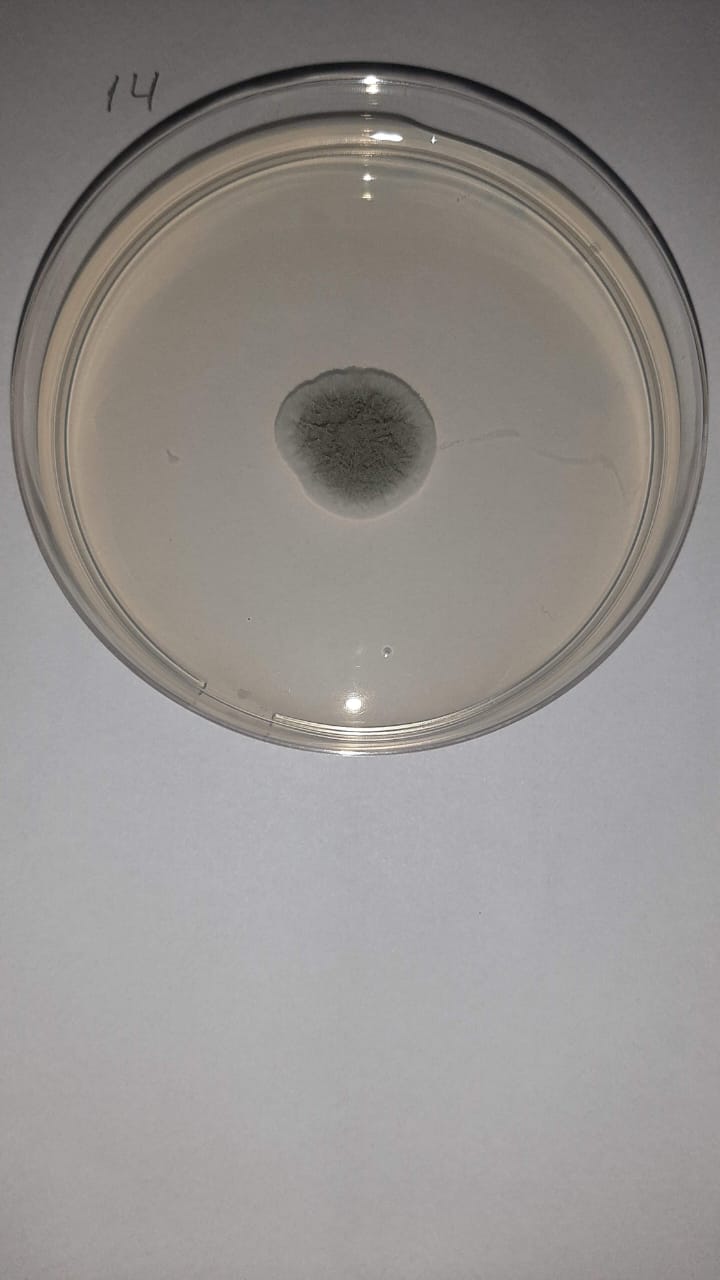

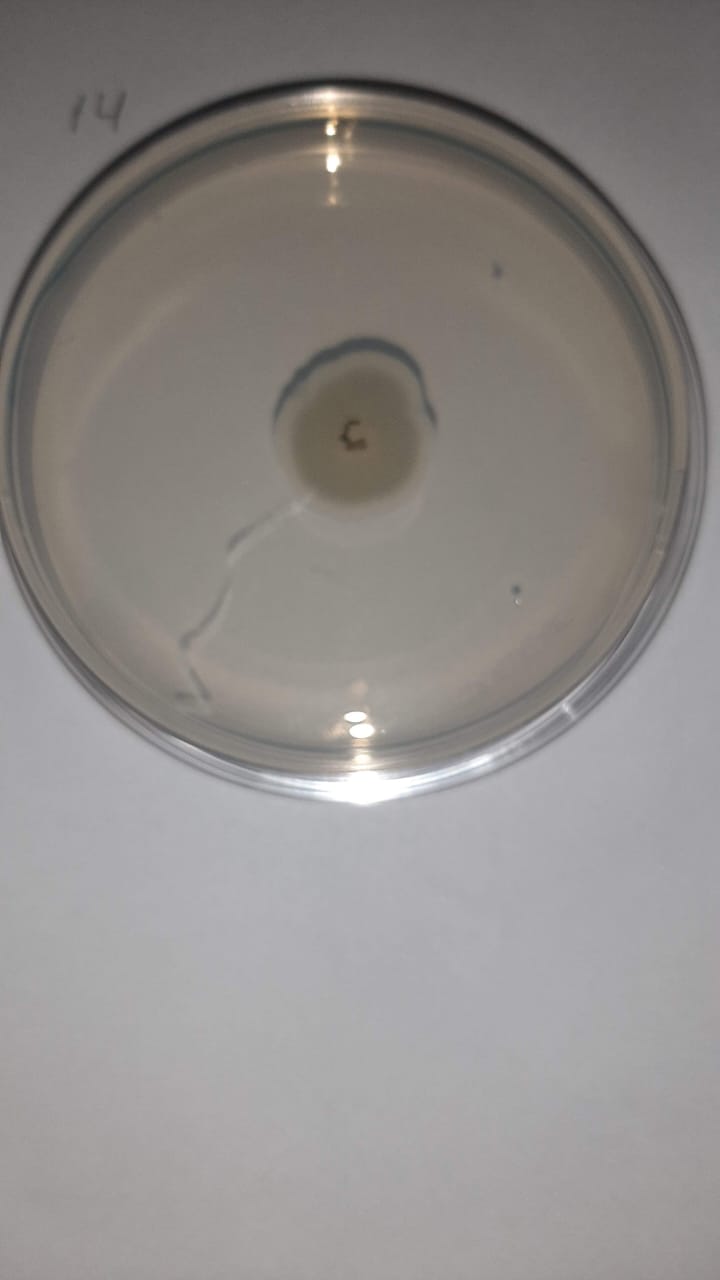


**LMC23014 -Up**

**LMC23014 -Down**


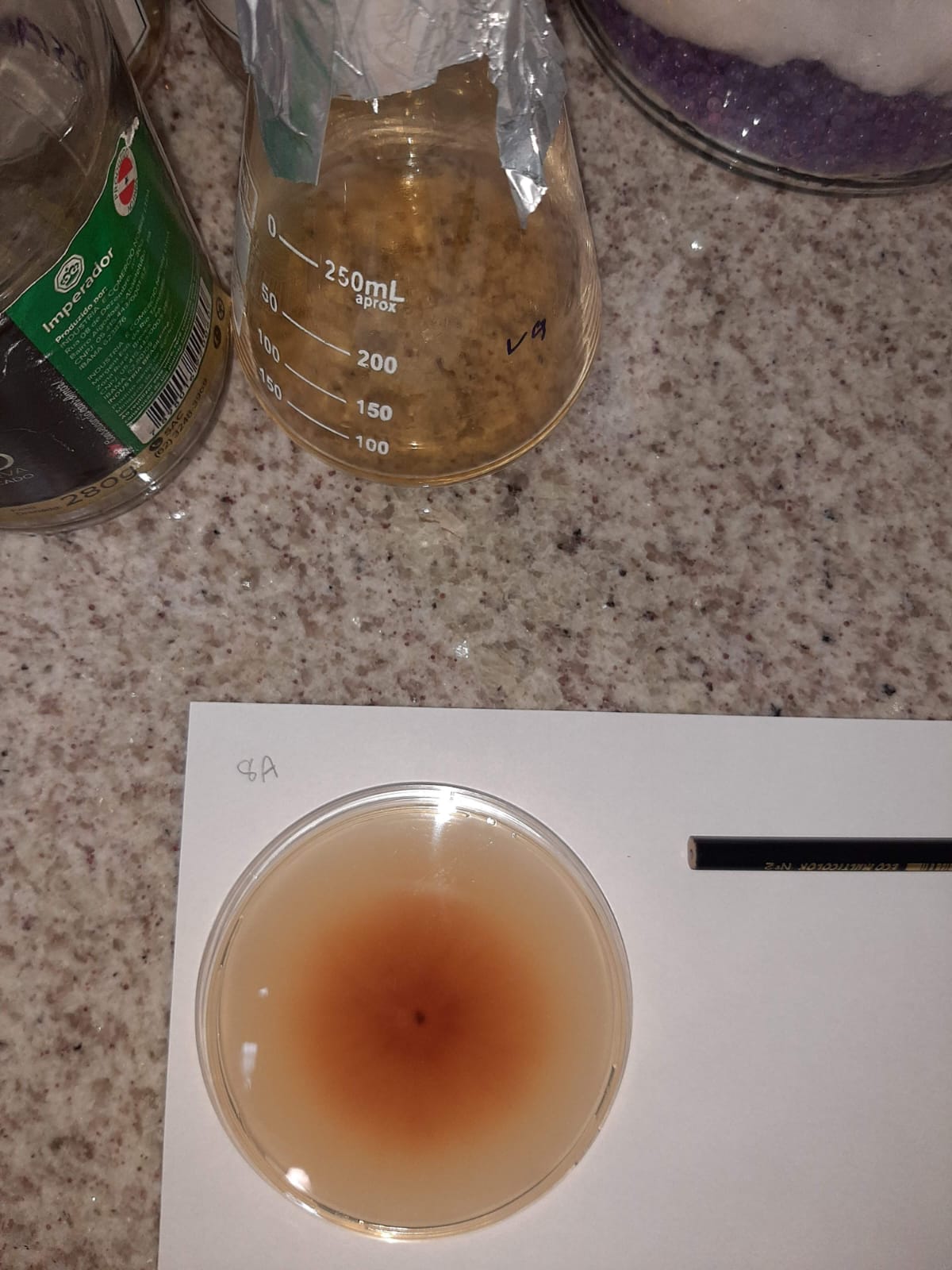

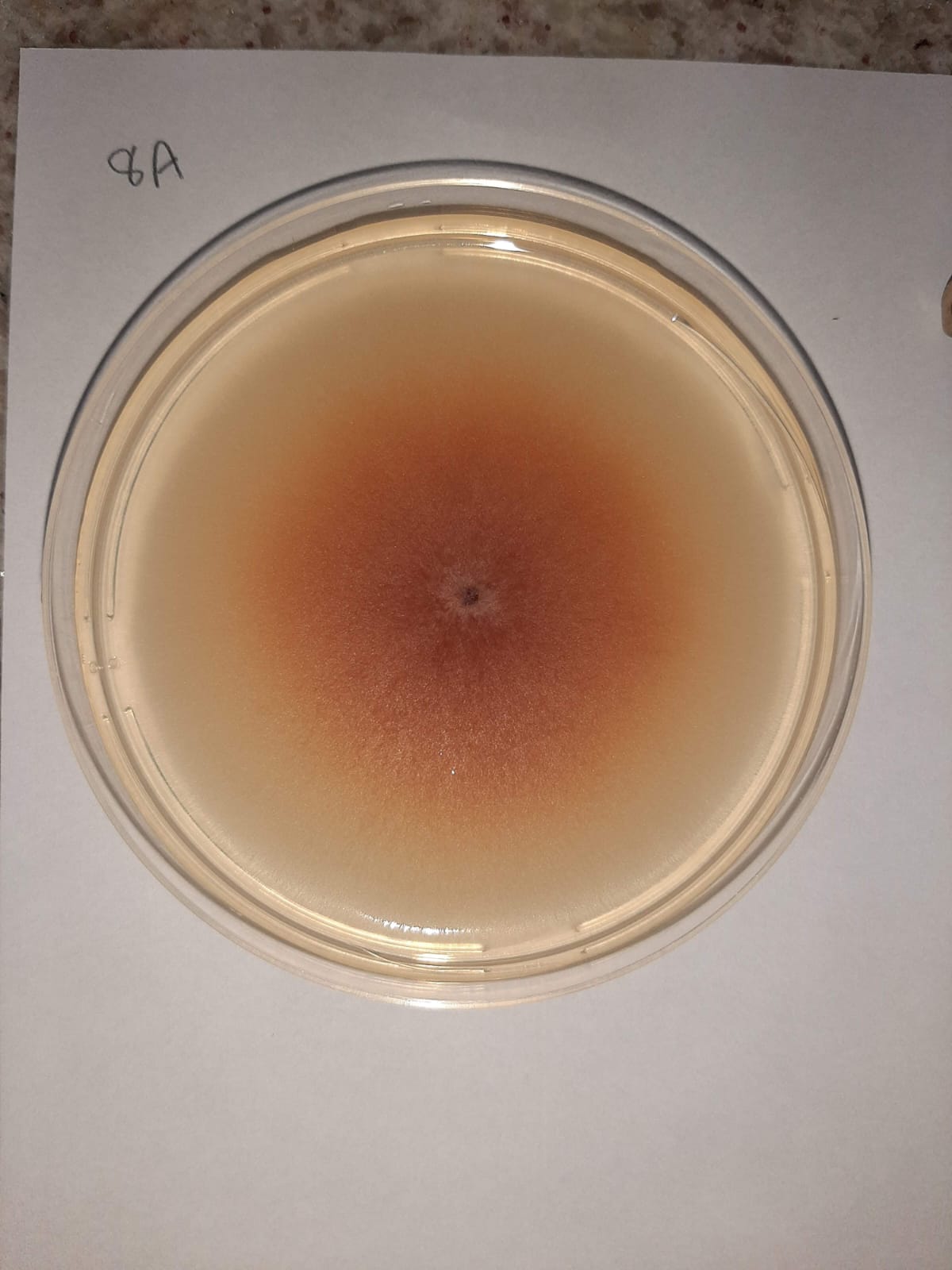

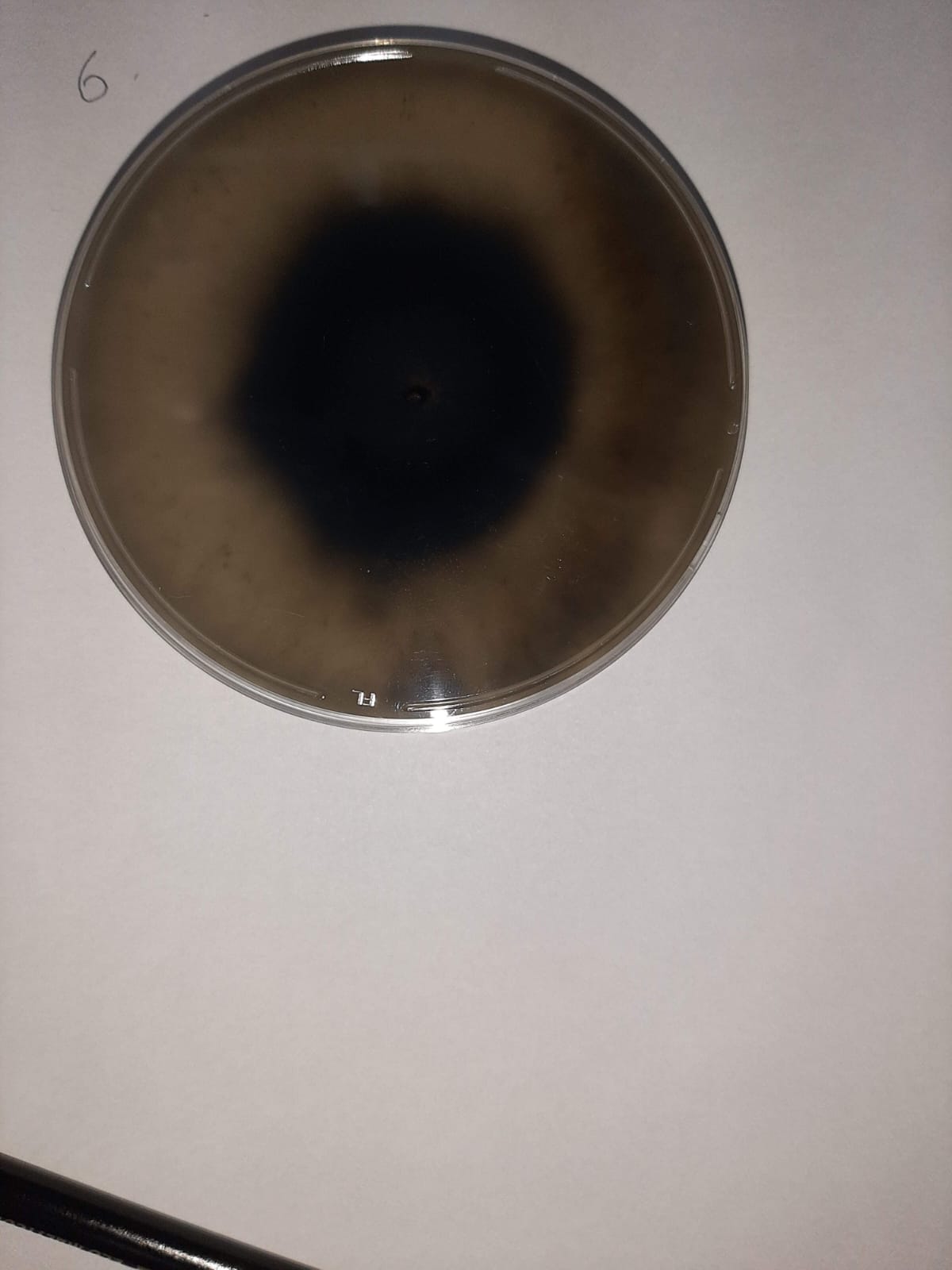

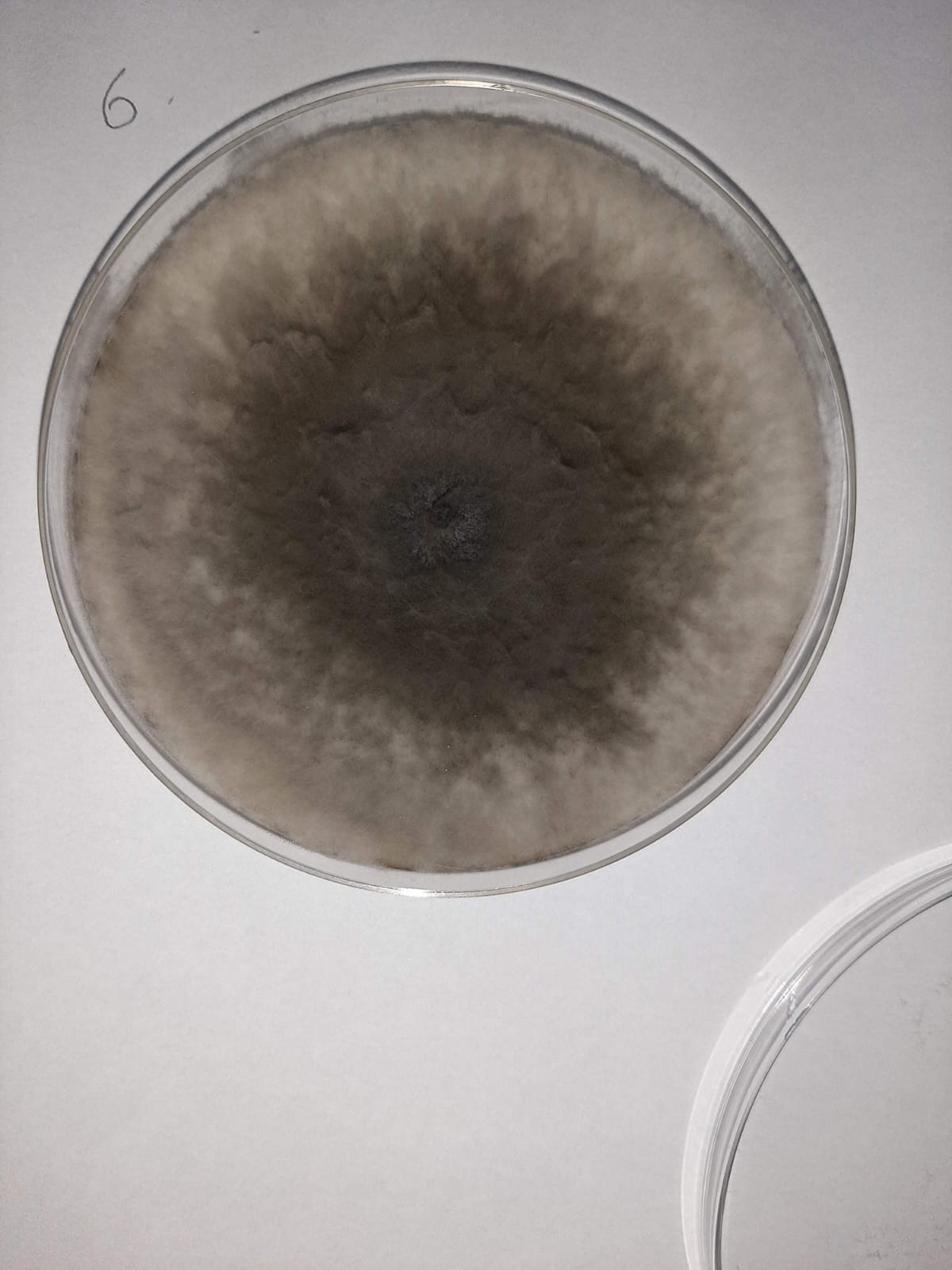

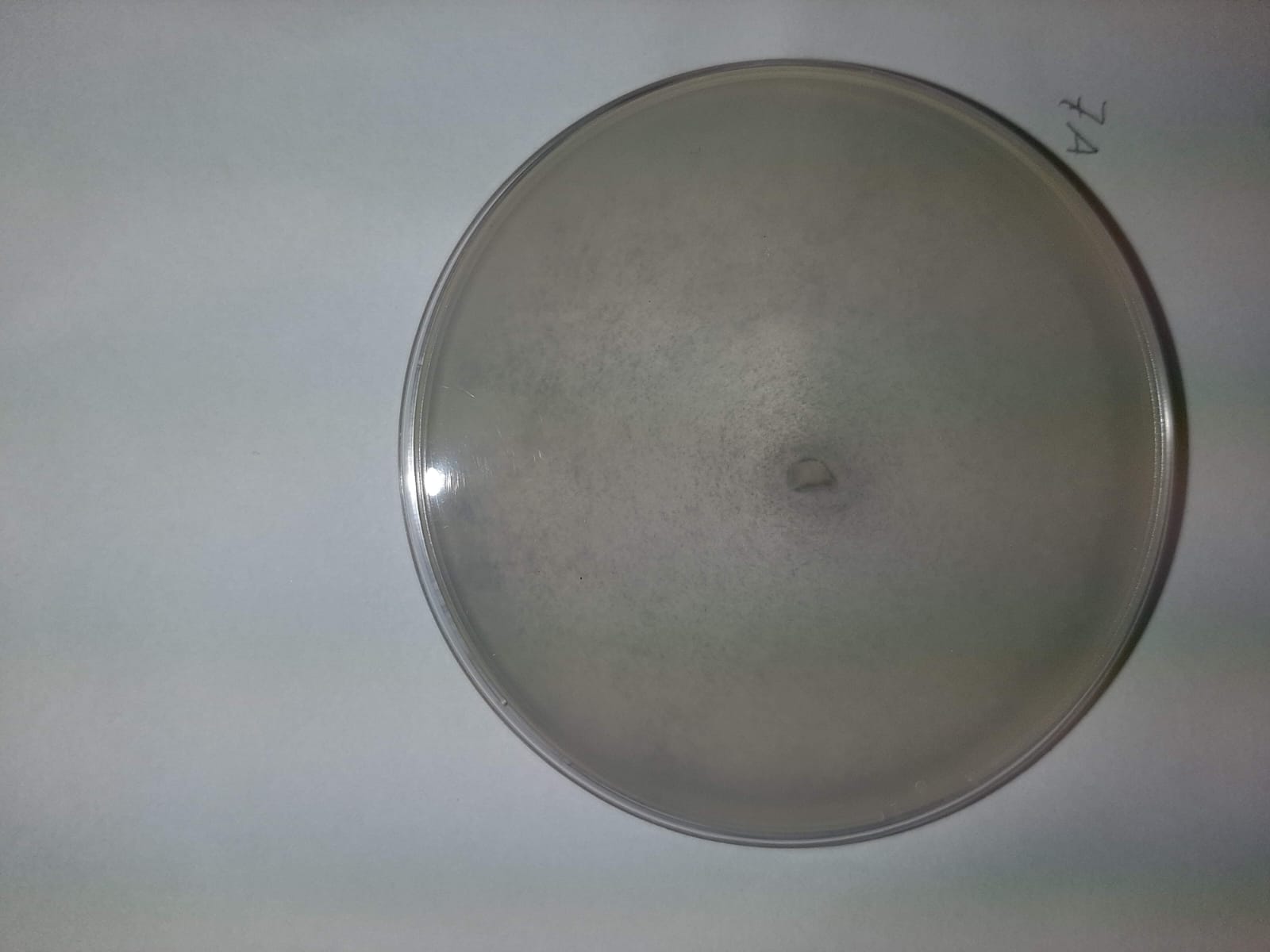

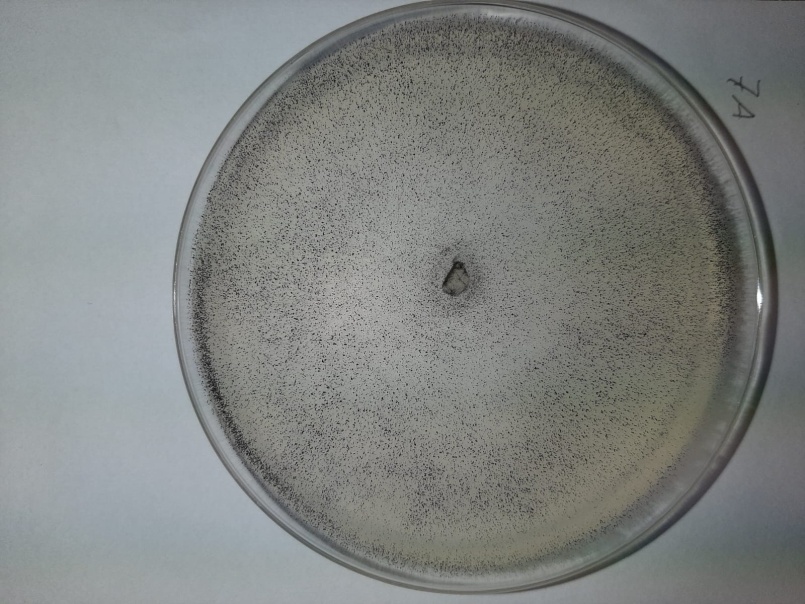

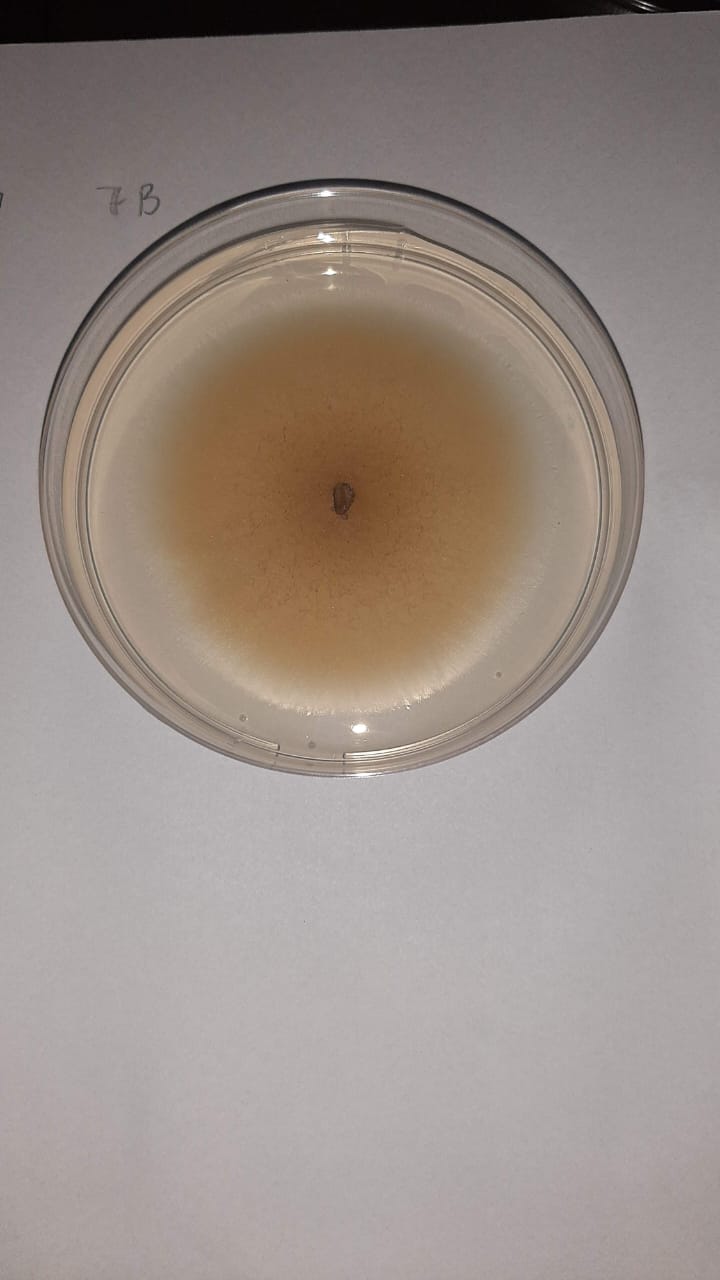

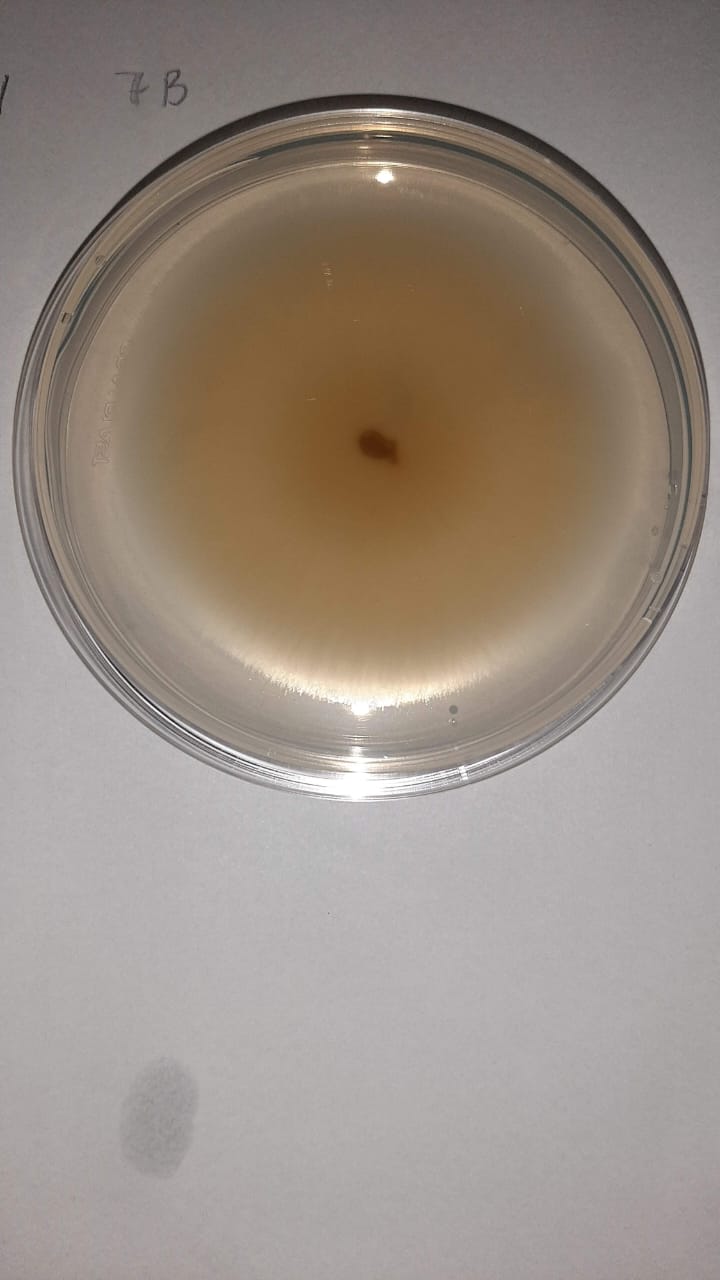


**LMC23006 - Up**

**LMC23006 - Down**

**LMC23007.1 - Up**

**LMC23007.1 - Down**

**LMC23007.2 - Up**

**LMC23007.2 - Down**

**LMC23008 - Up**

**LMC23008 - Down**


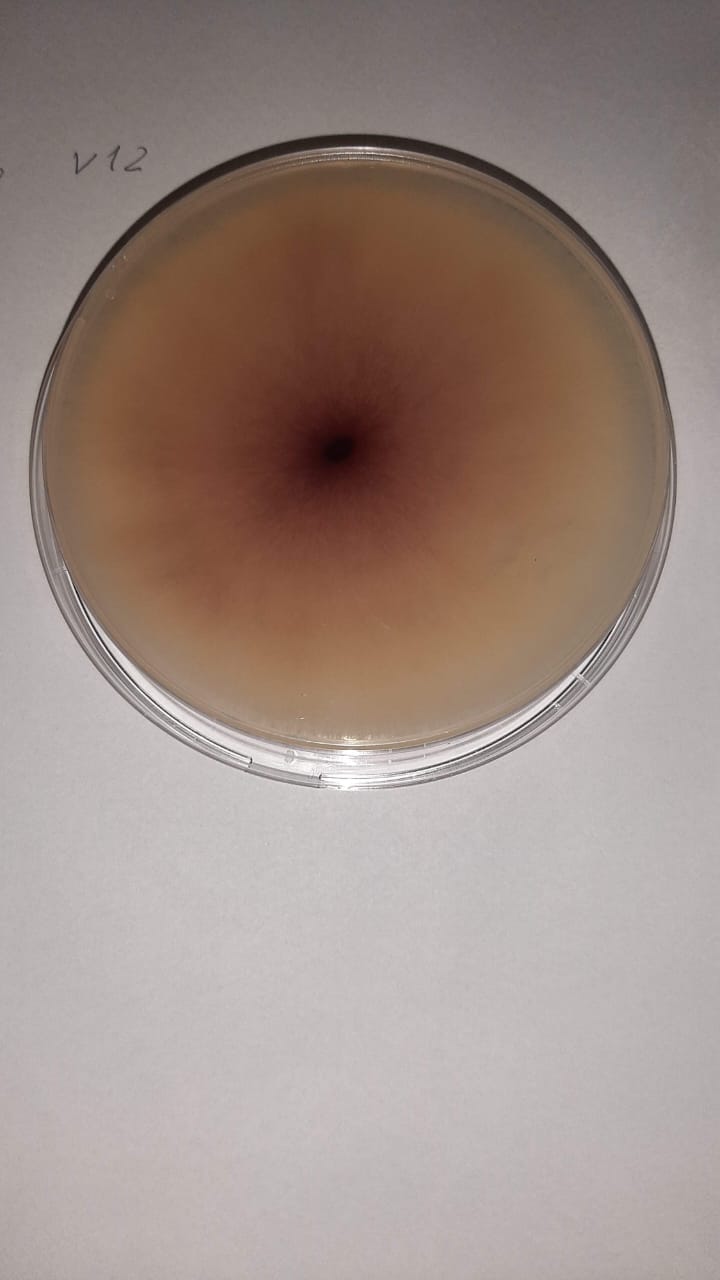

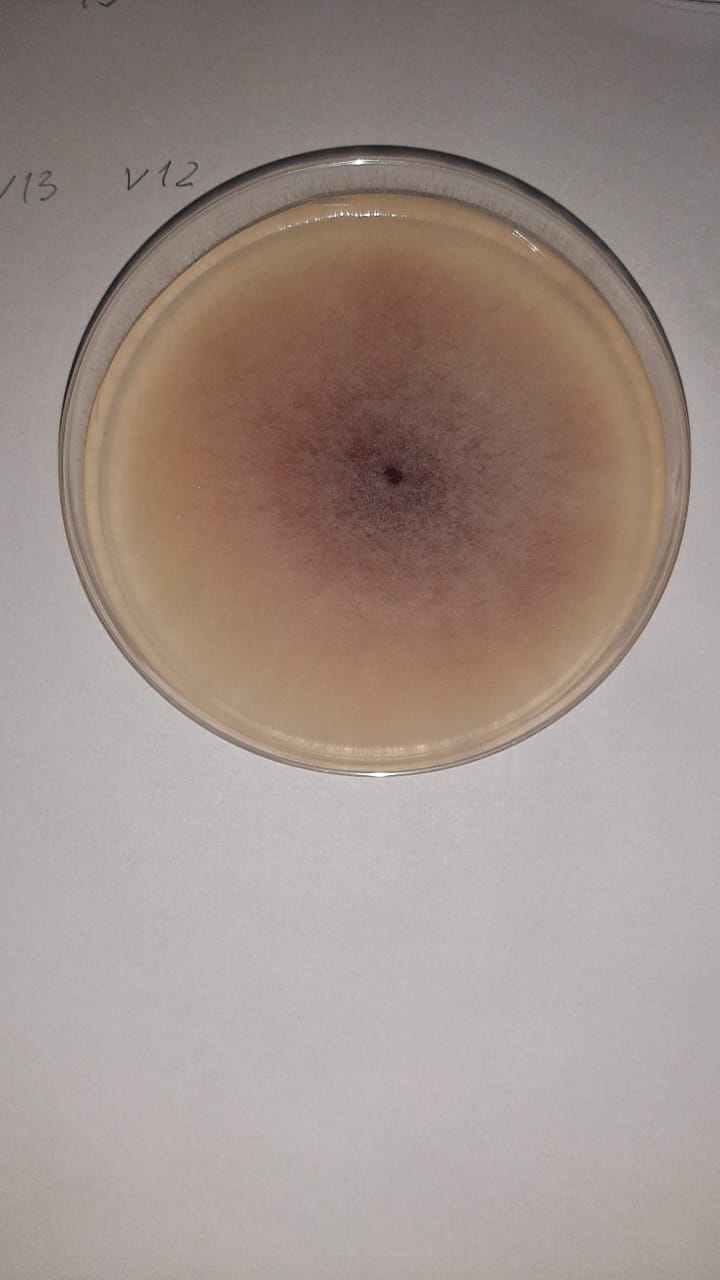

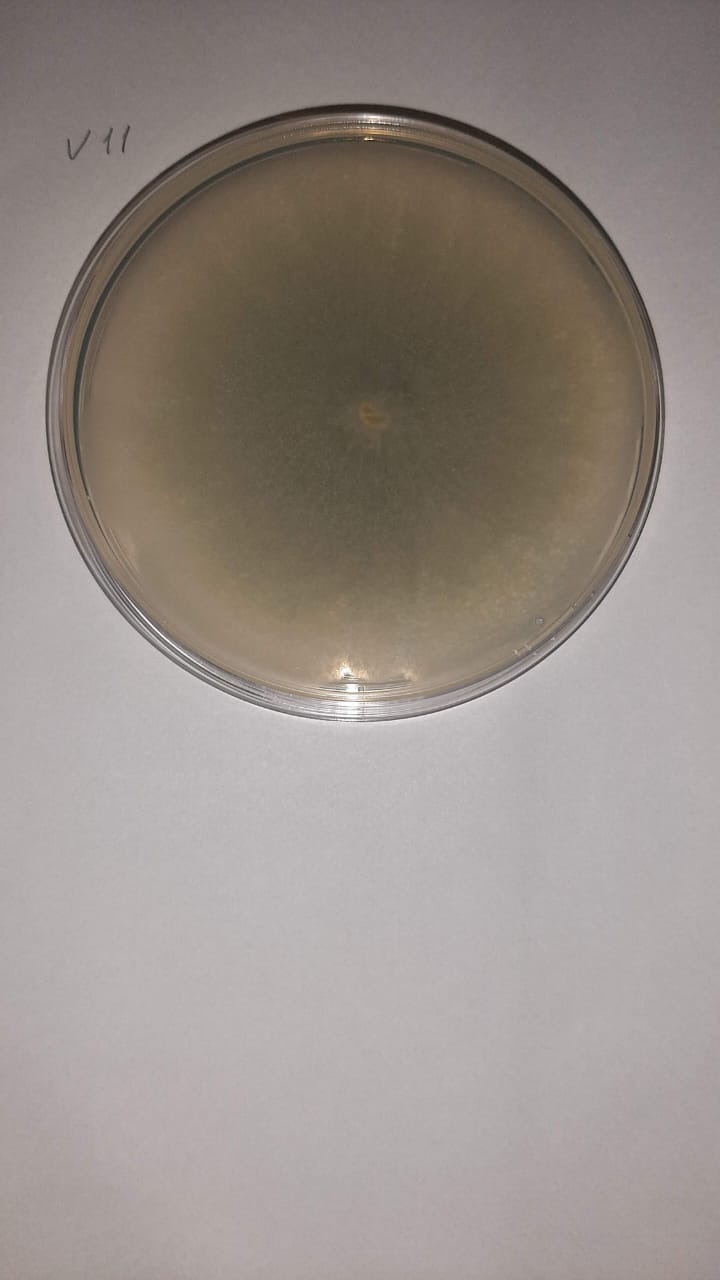

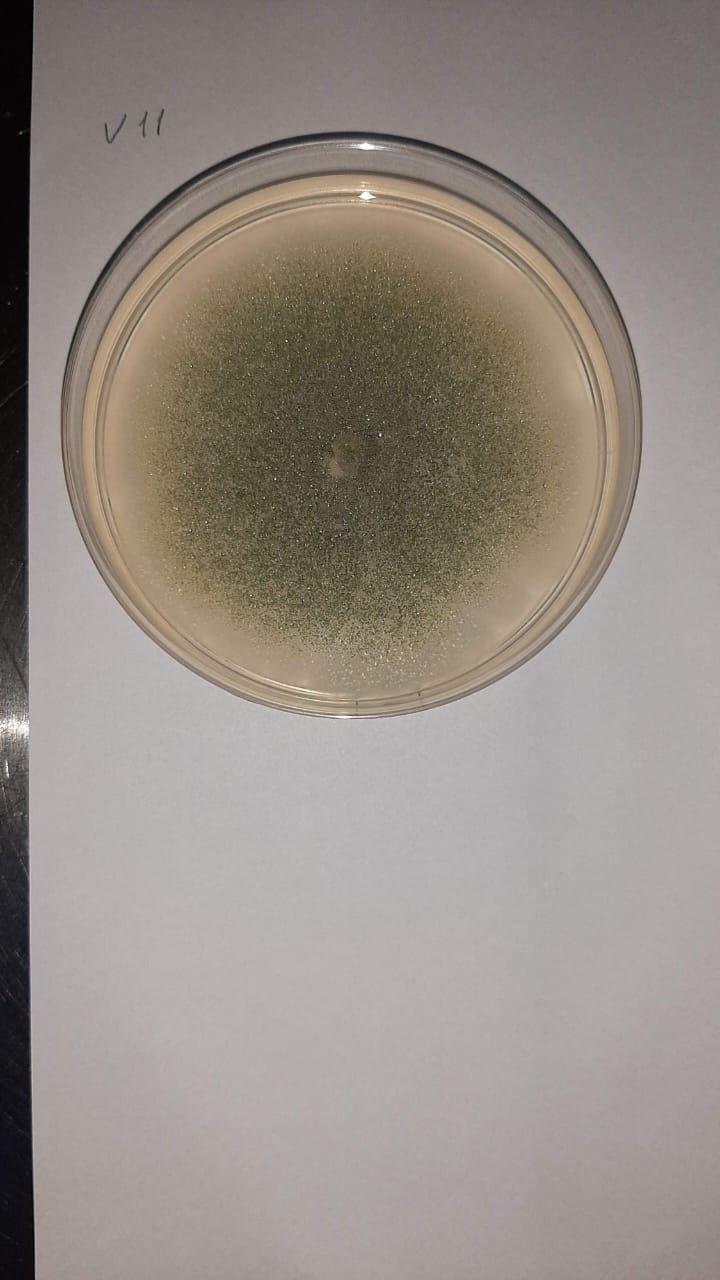

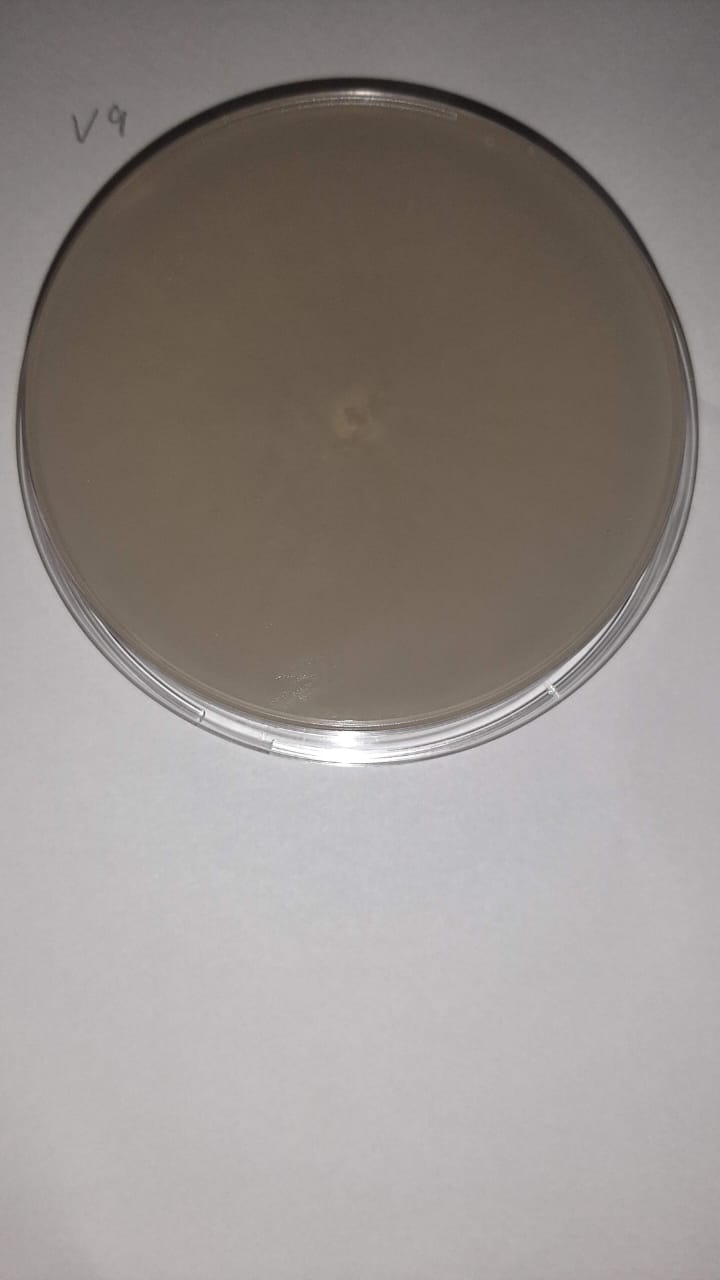

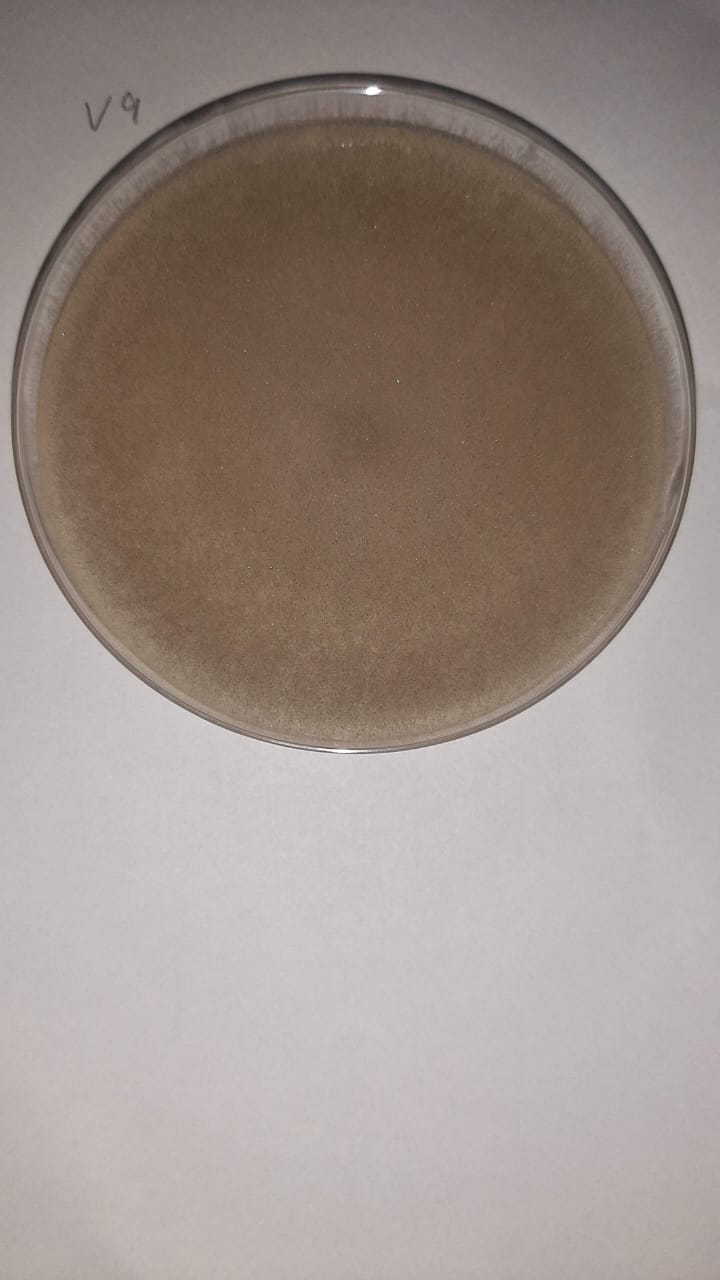


**LMC23009 - Up**

**LMC23009 - Down**

**LMC23011 - Up**

**LMC23011 - Down**

**LMC23012 - Up**

**LMC23012 - Down**


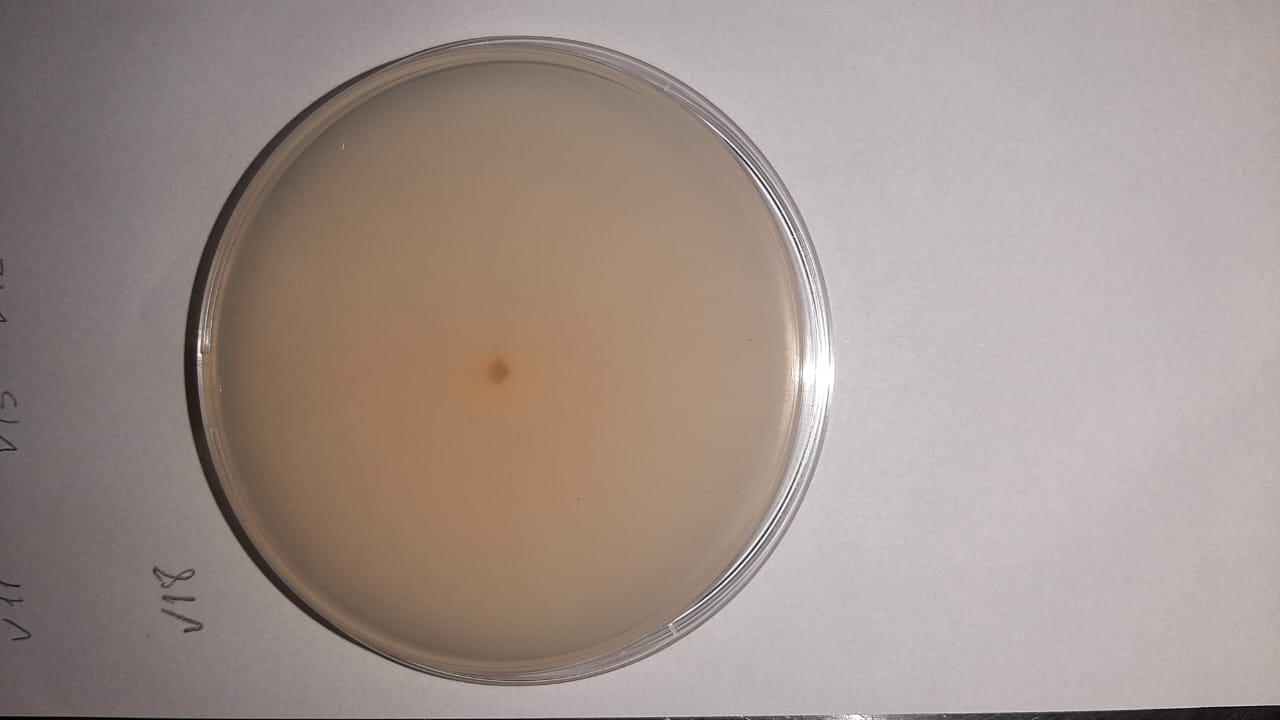

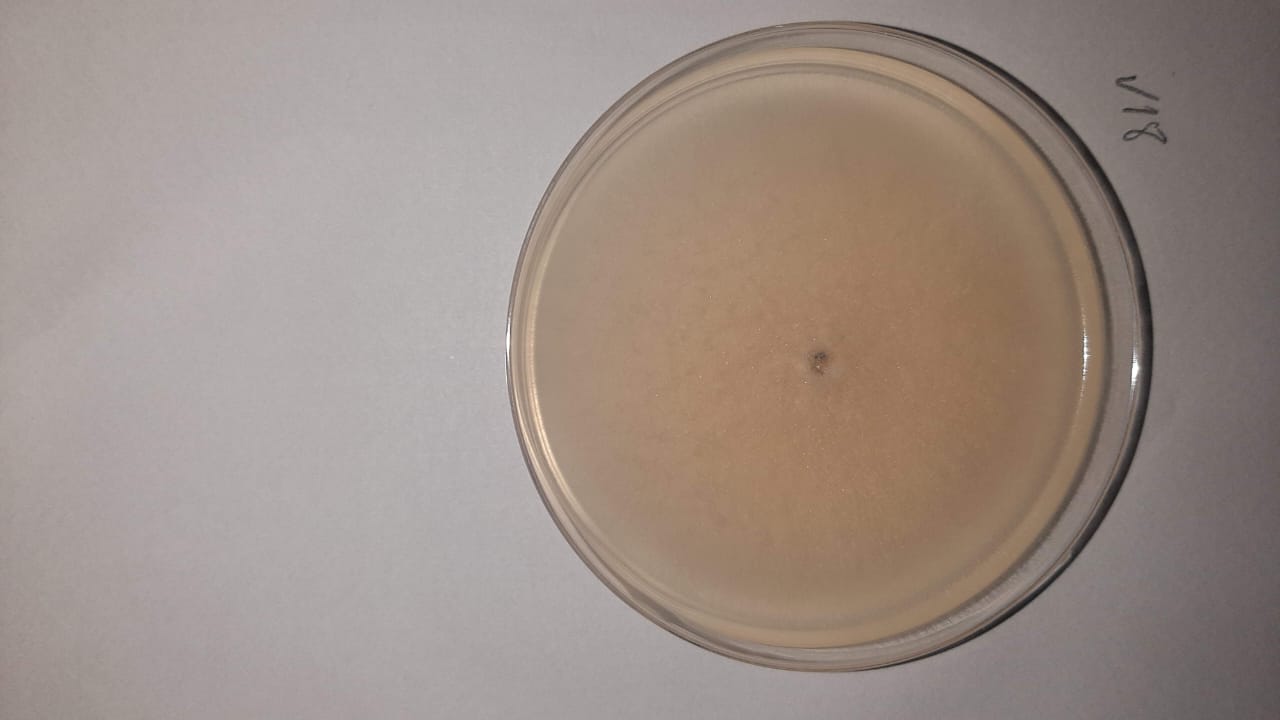

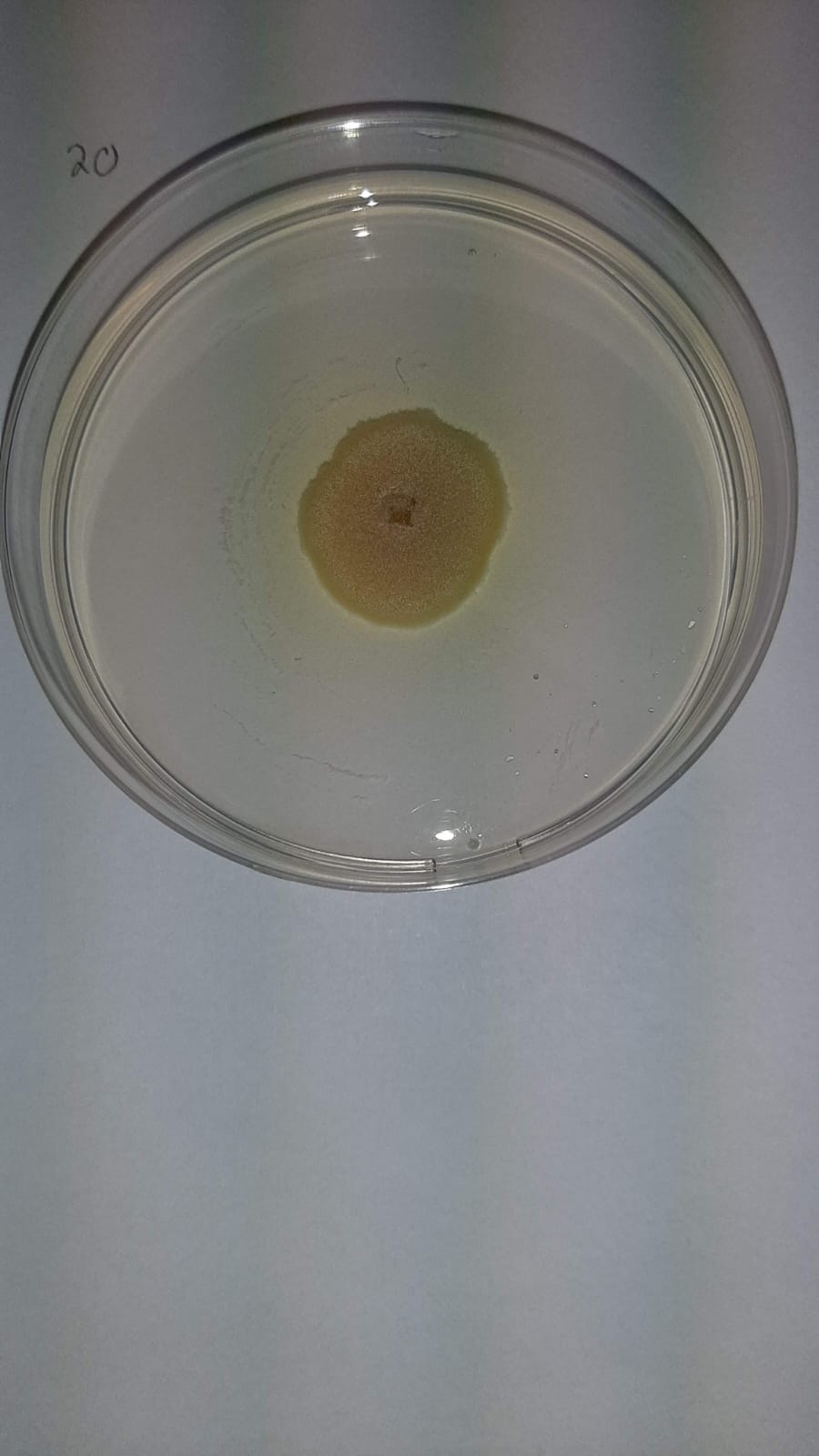

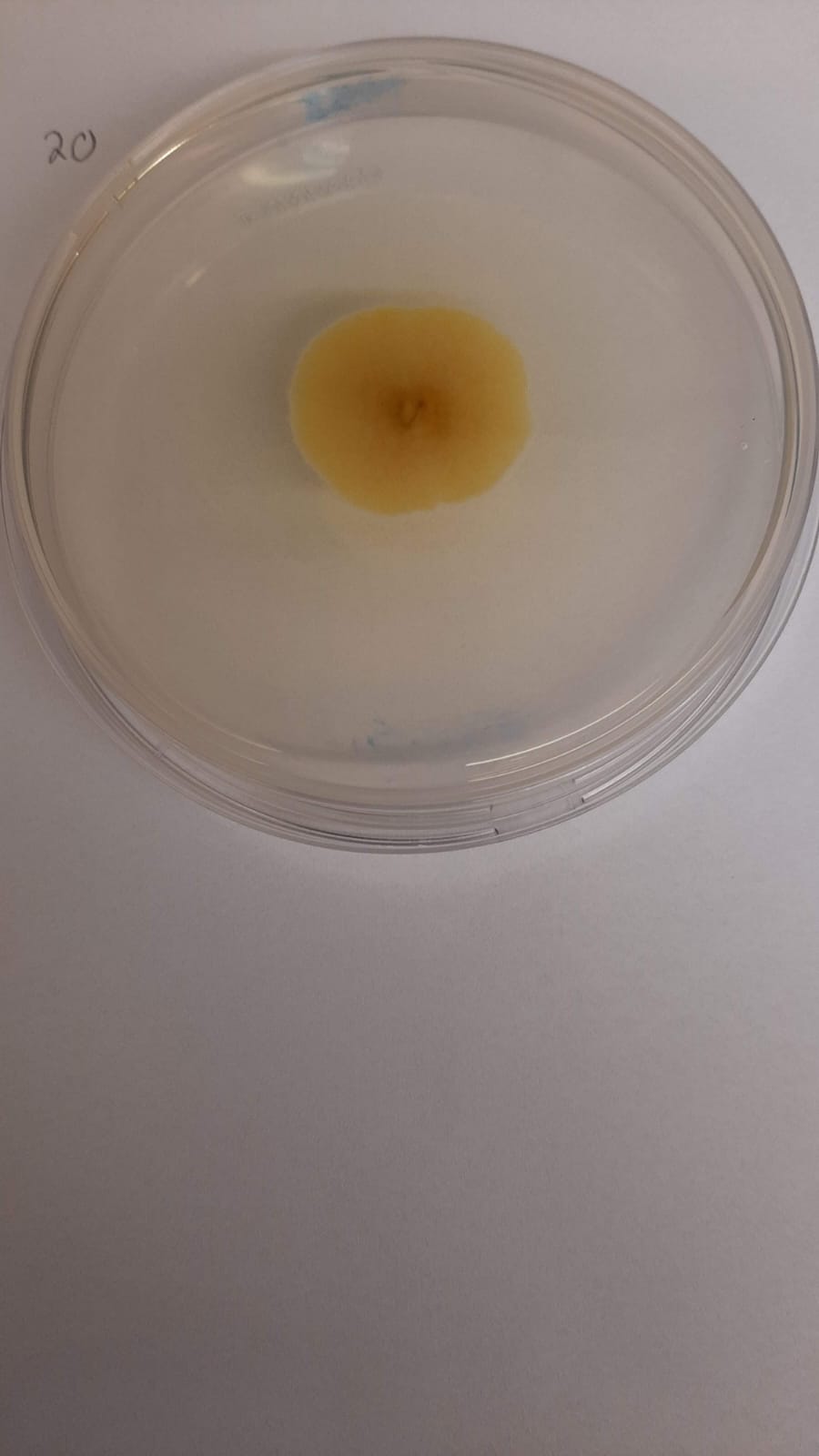


**LMC23018 - Up**

**LMC23018 - Down**

**LMC23020 - Up**

**LMC23020 - Down**


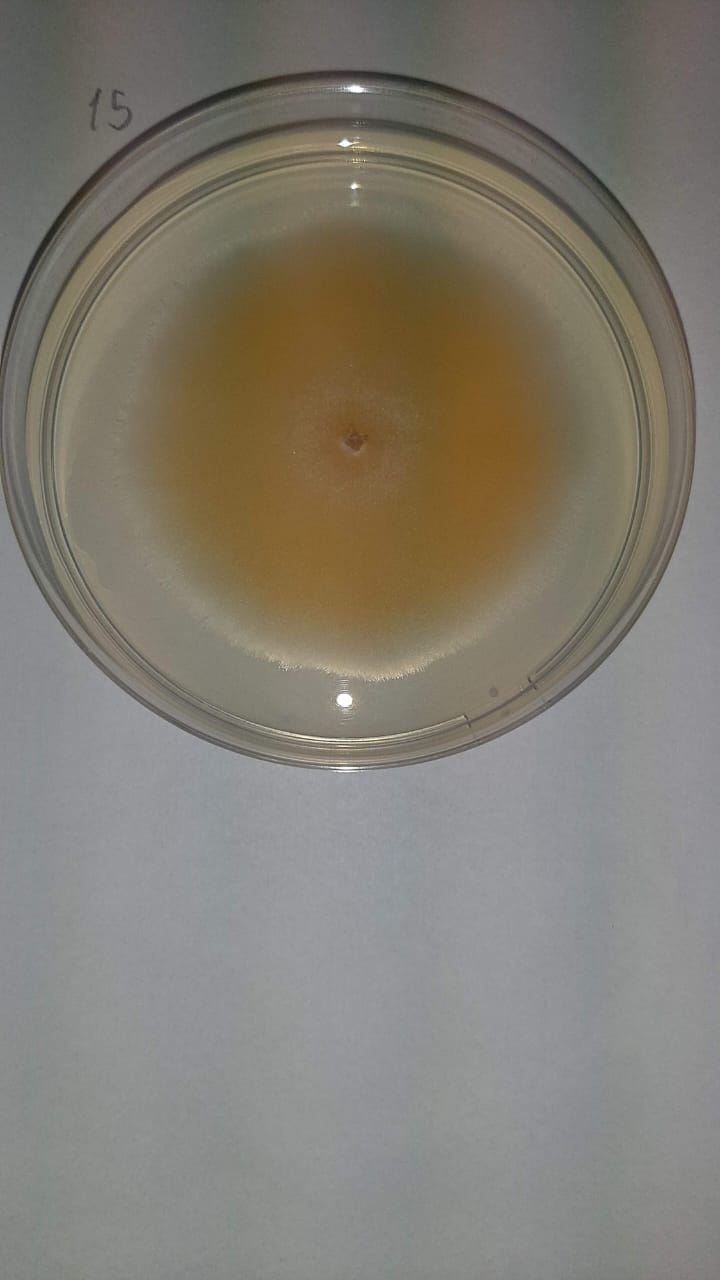

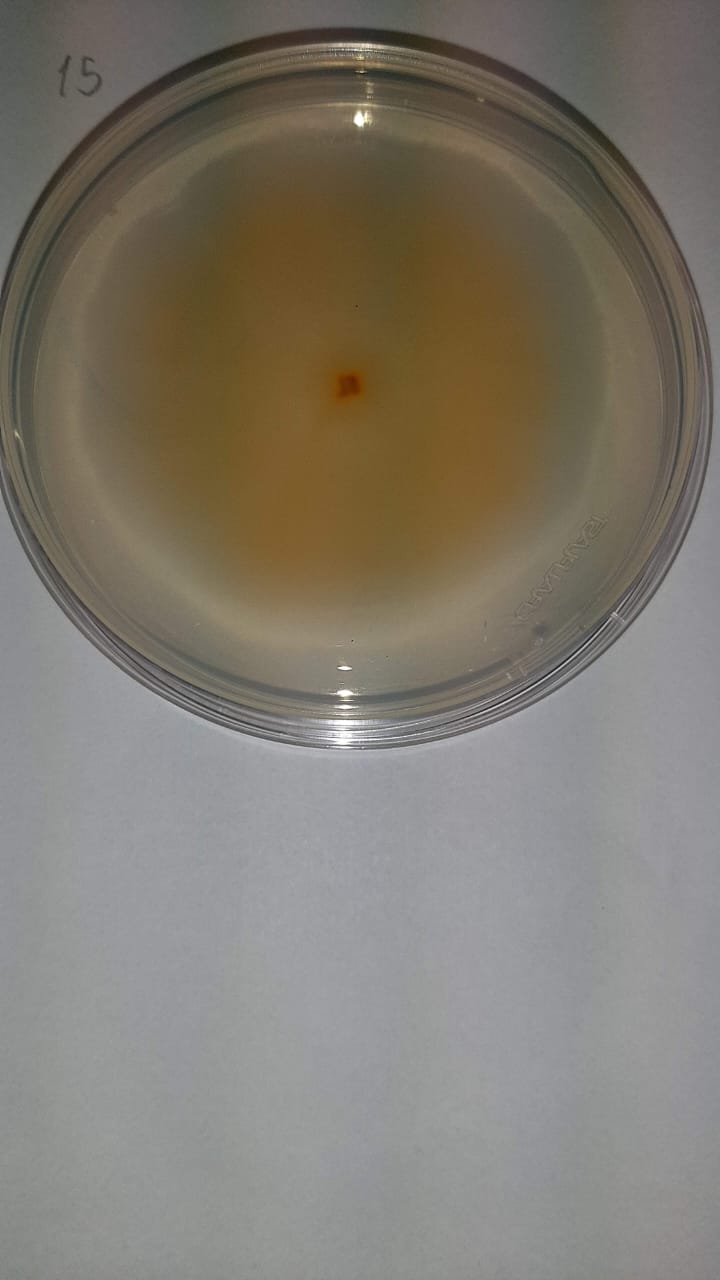

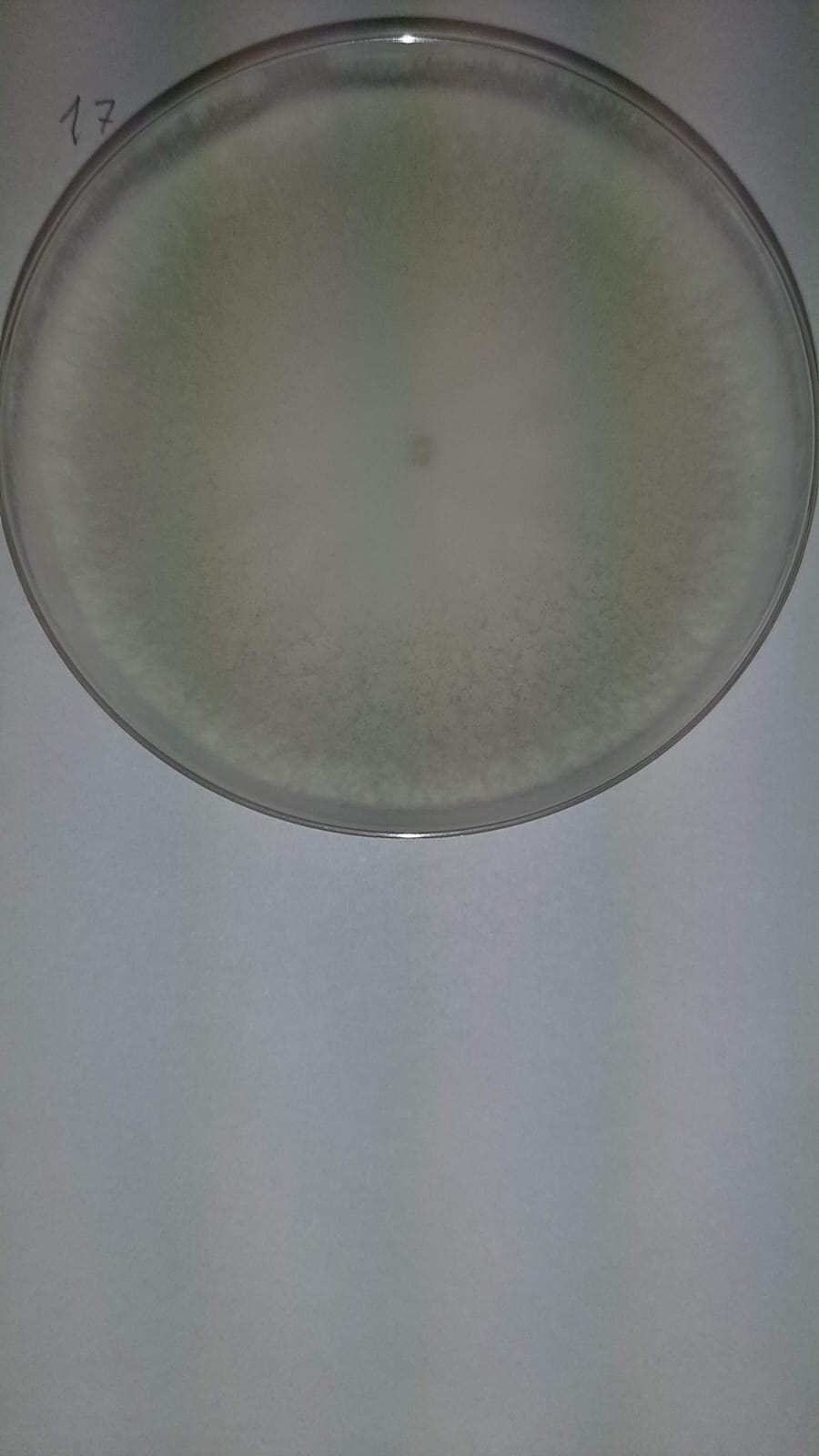

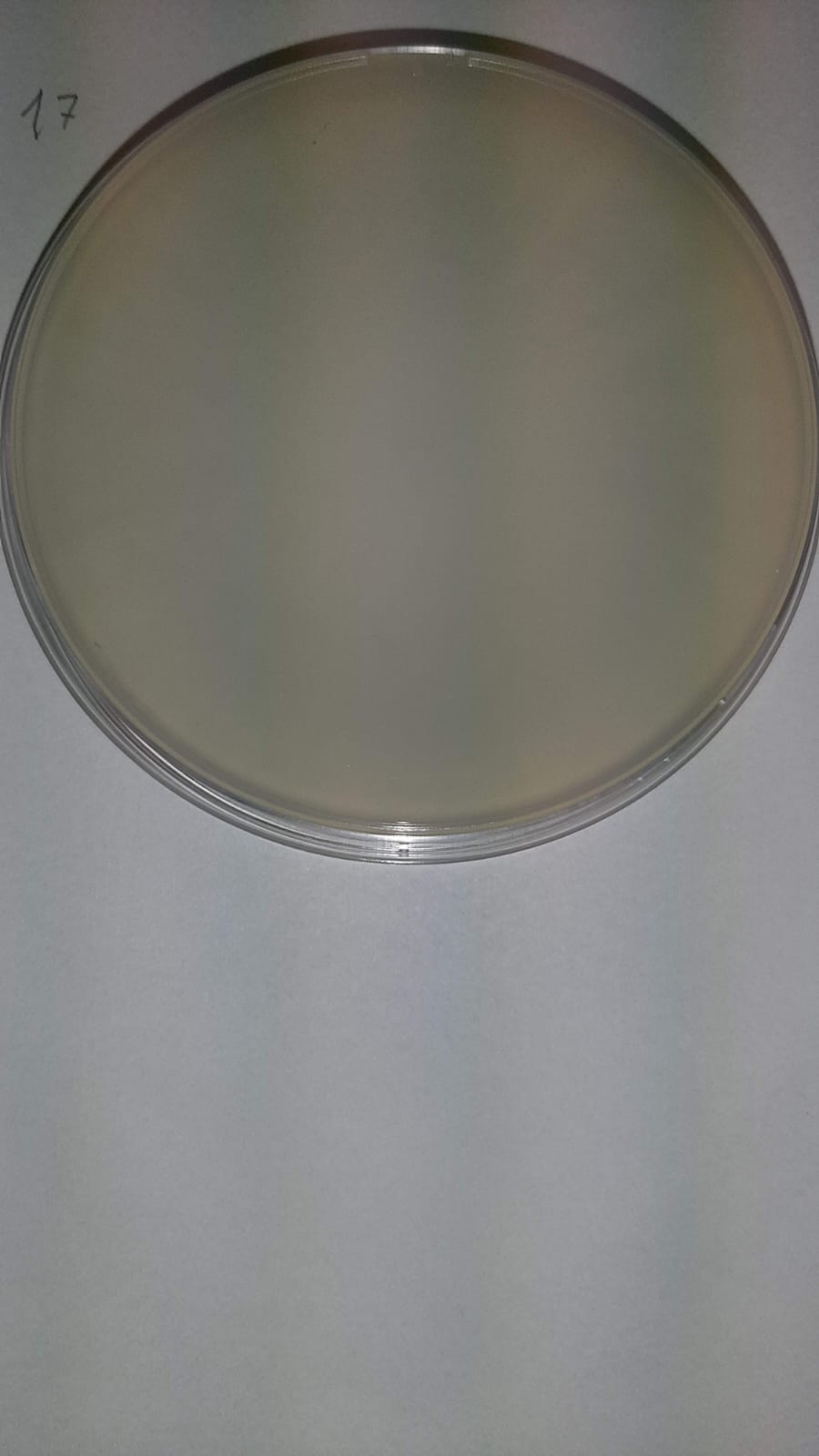


**LMC23015 -Up**

**LMC23015 -Down**

**LMC23017 -Up**

**LMC23017 -Down**

# **Fig. S1** Photos of the fungal plates growing on PDA.


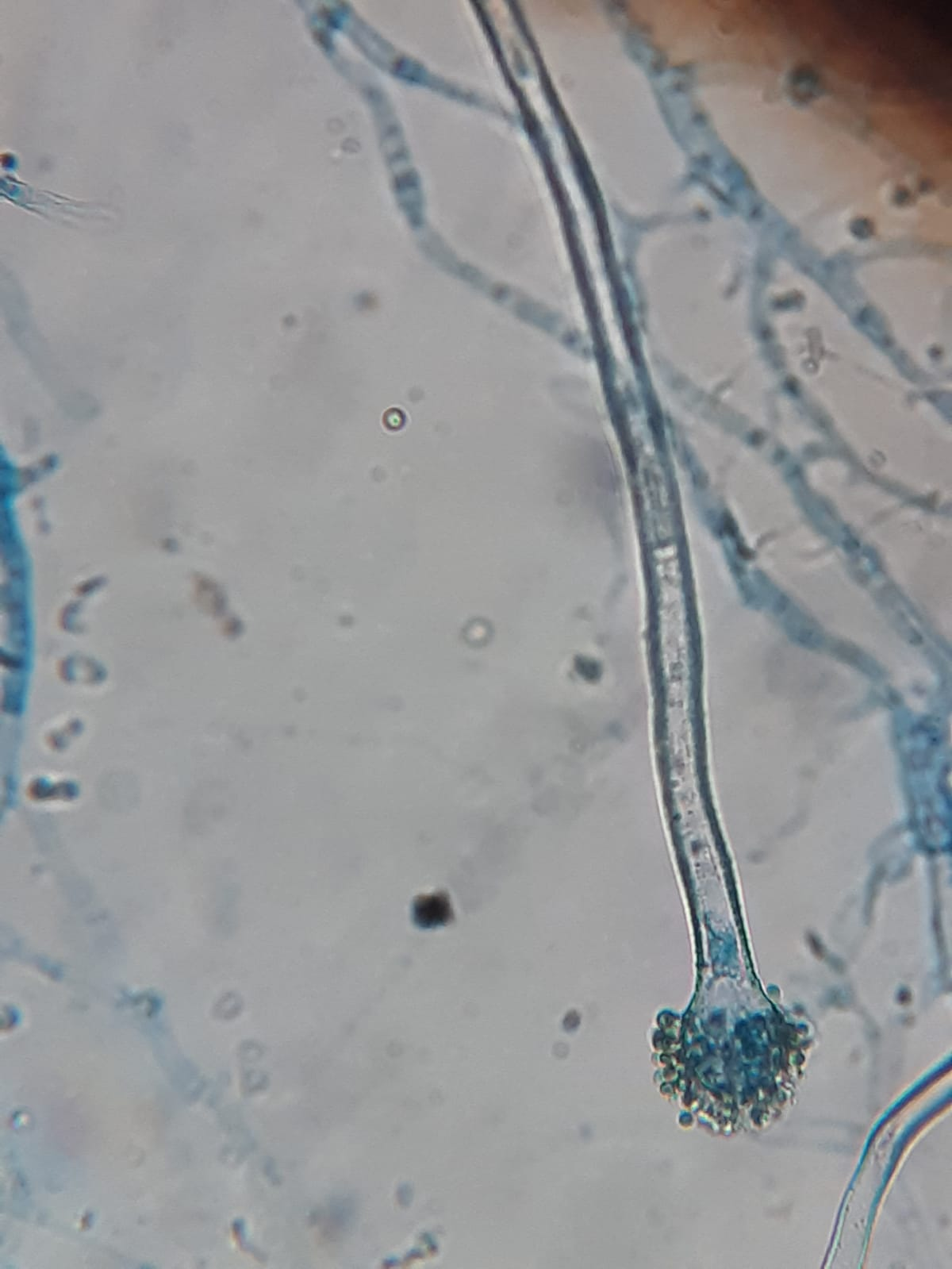

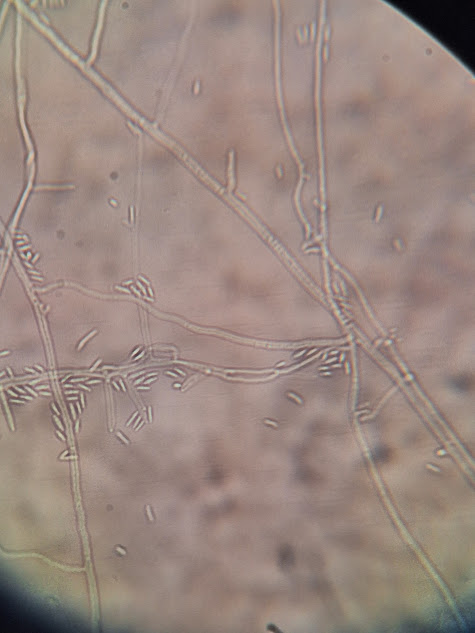

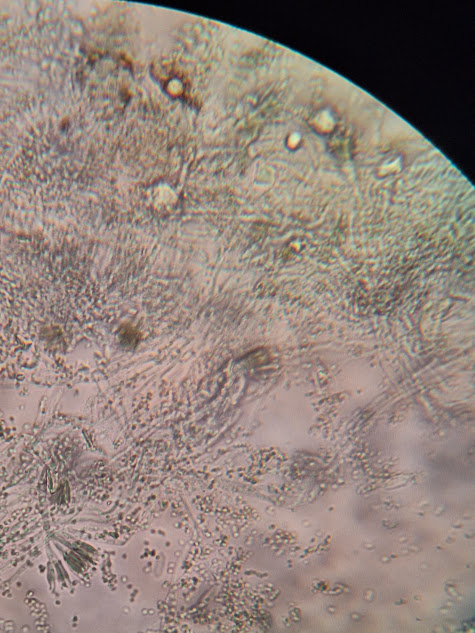

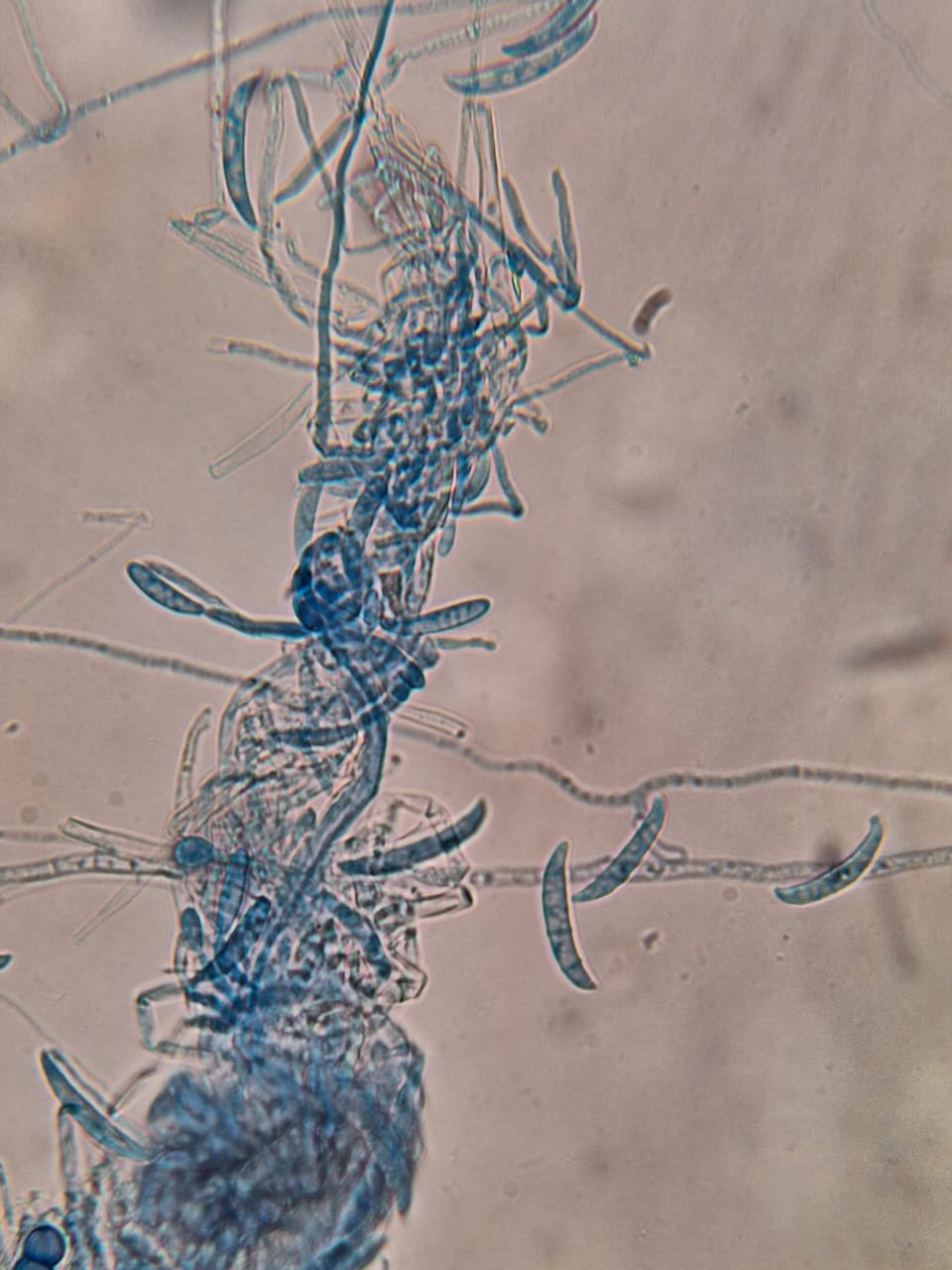

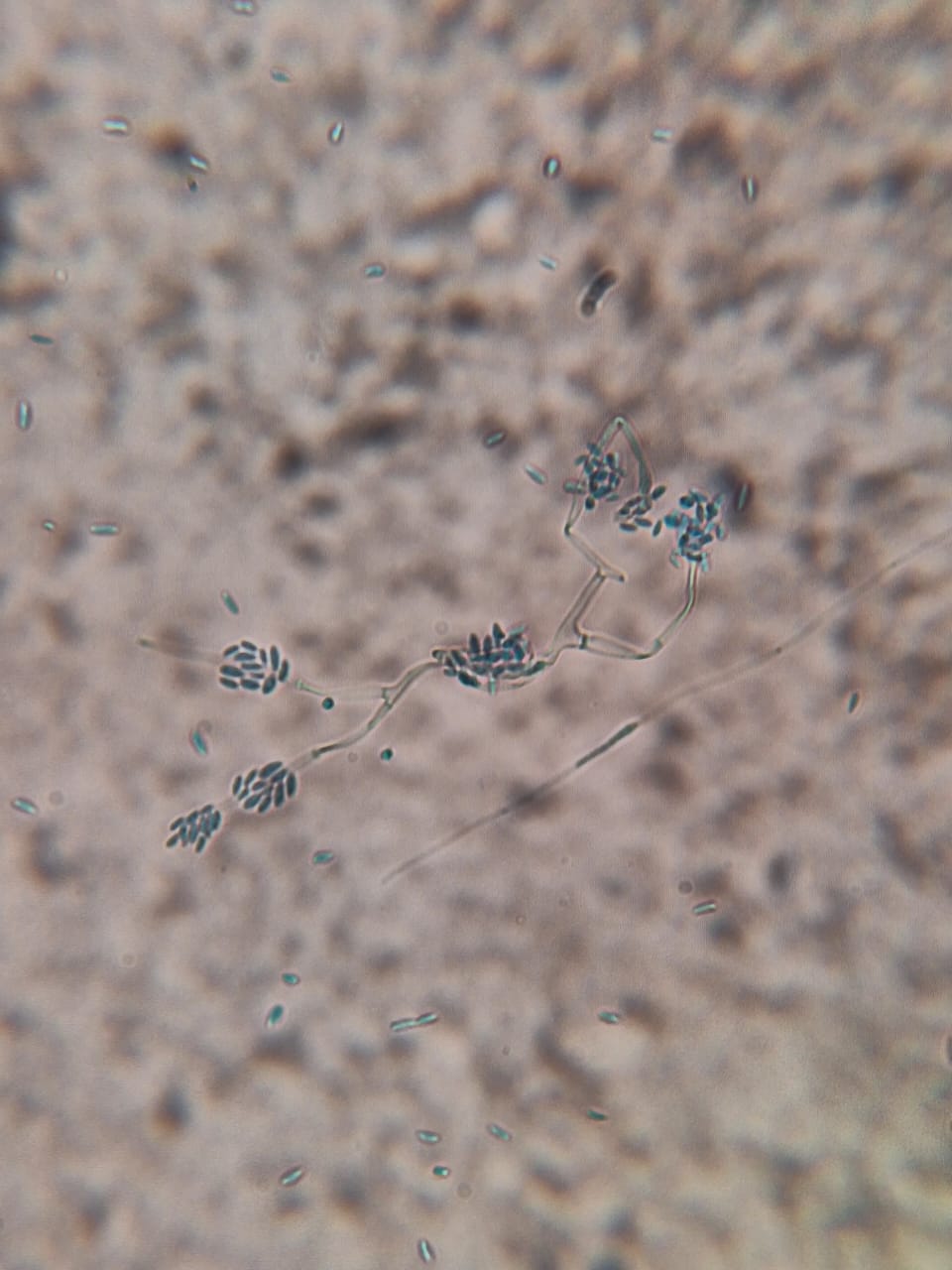

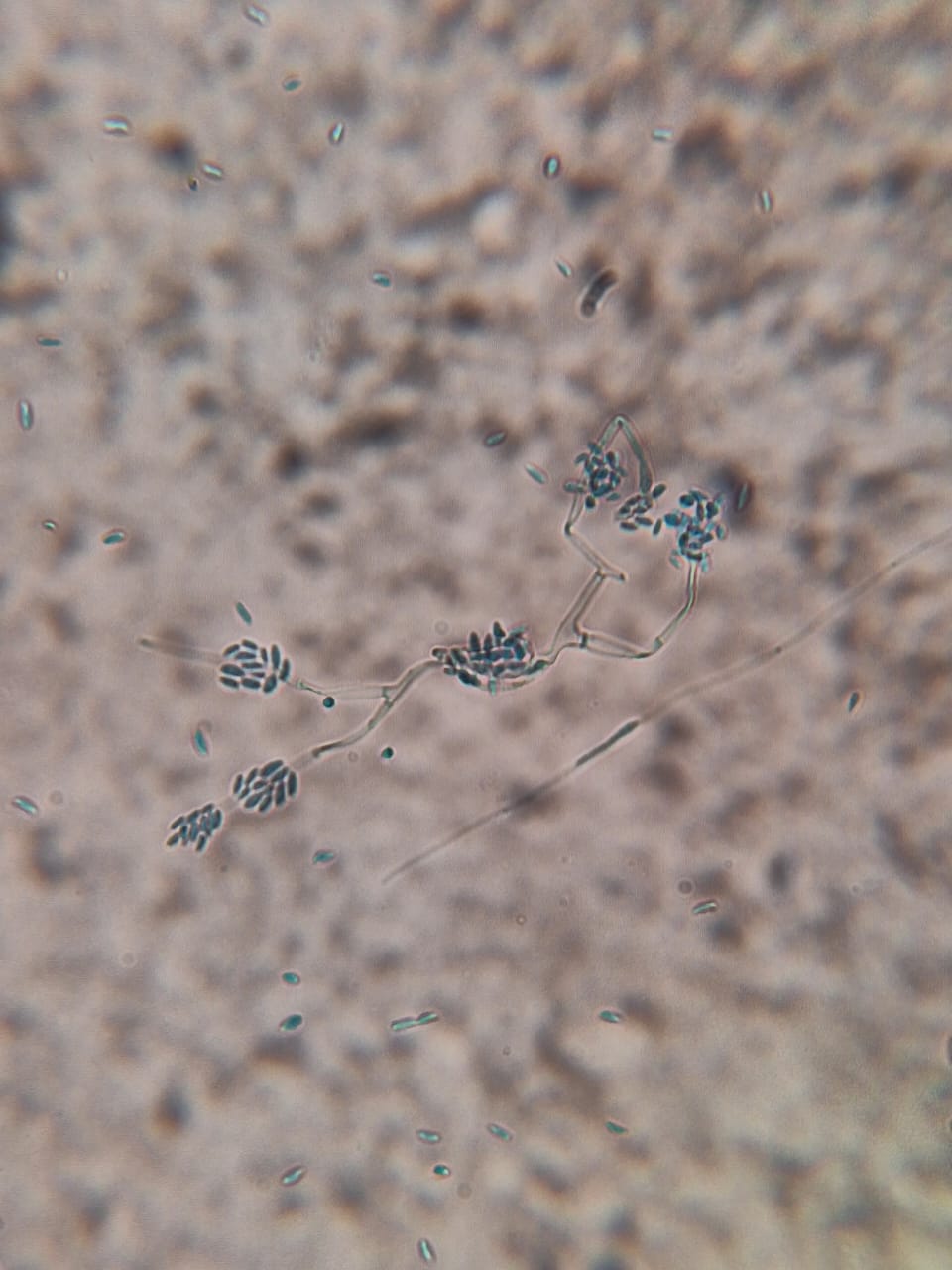

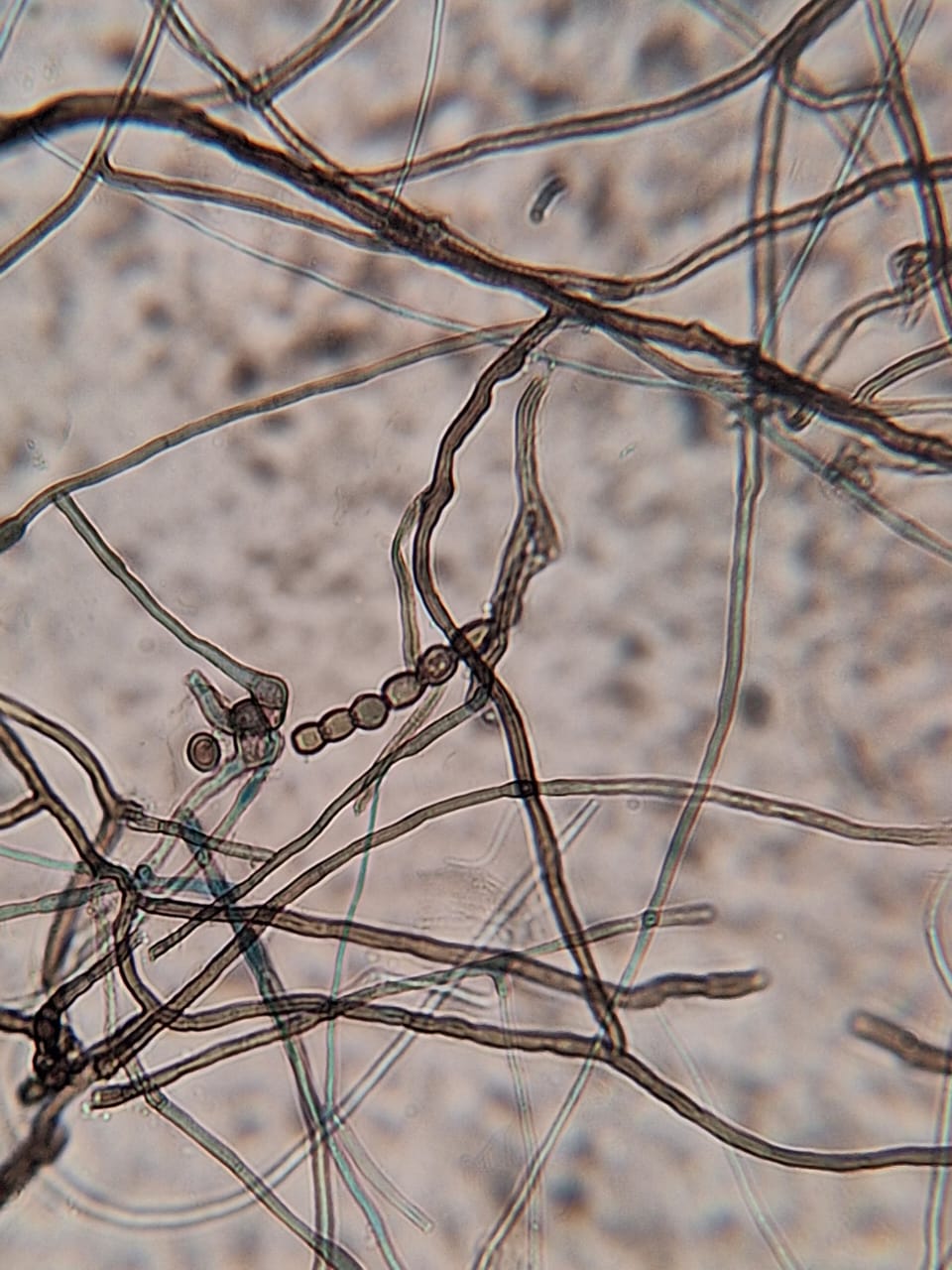


**LMC23006**

**LMC2307.2**

**LMC23012**

**LMC2307.1**

**LMC23008**

**LMC23011**

10 µm

10 µm

10 µm

10 µm

10 µm

10 µm

**LMC23014**

10 µm


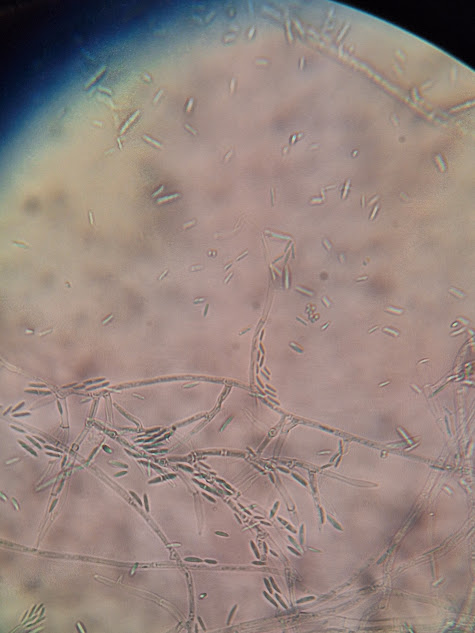

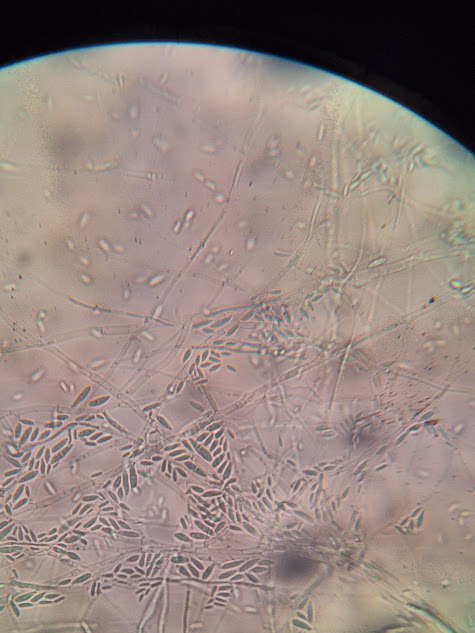

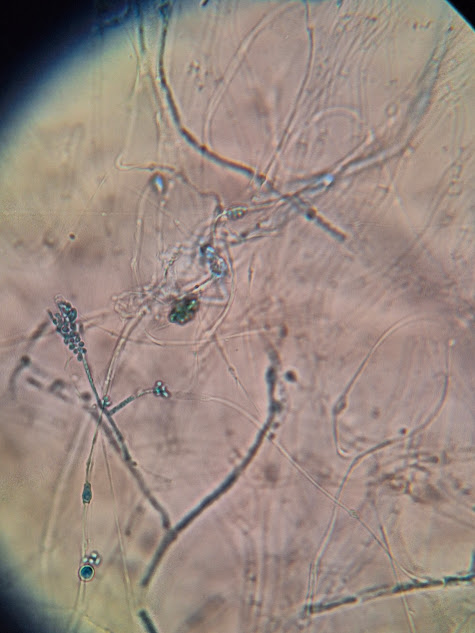


**LMC23015**

10 µm

**LMC23018**

10 µm

**LMC23017**

10 µm


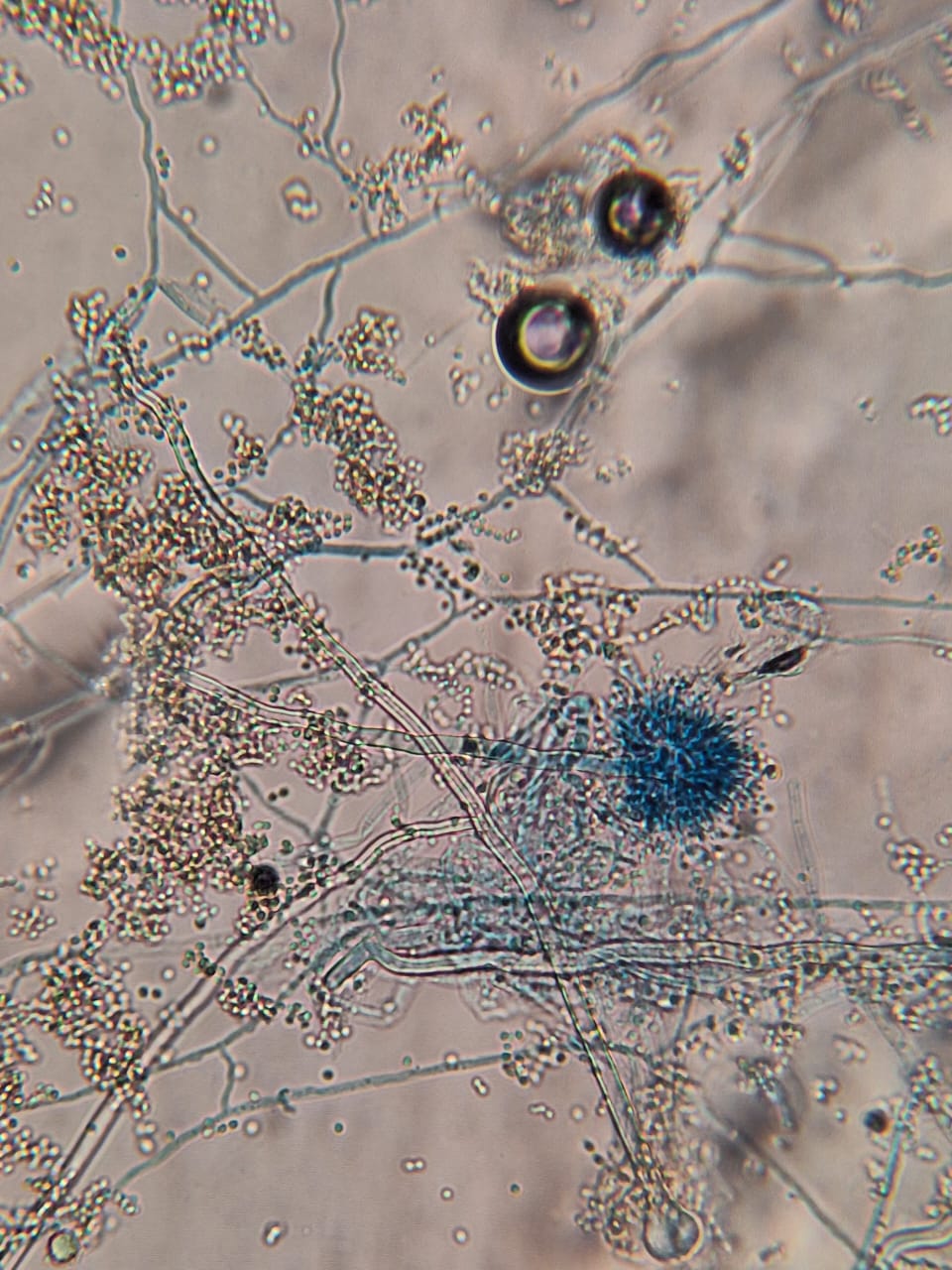


**LMC23020**

10 µm

# **Fig. S2** Morphology of fungi isolated from papaya and pineapple observed under the microscope.

# **Table S1** Reference strains and GenBank accession numbers for *ITS*, *TUB2*, and *RPB2* sequences used in phylogenetic analysis of *Neofusicoccum* sp.

| **Species** | **Fungal Code**^1^ | ***ITS*** | ***Tub2*** | ***RPB2*** |
| --- | --- | --- | --- | --- |
| *B. ribis* | CBS 118822 | DQ316075 | JF440836 | MT592440 |
| *N. umdonicola* | CBS 123646 | EU821905 | EU821845 | EU821935 |
| *N. ribis* | CBS 122553 | EU683673 | MT592742 | MT592439 |
| *N. umdonicola* | CBS 117915 | MT587517 | MT592733 | MT592430 |
| *N. ribis* | CBS 114306 | MT587514 | MT592721 | MT592418 |
| *B. ribis* | CBS 115475 | AY236935 | AY236906 | EU821958 |
| *N. batangarum* | CBS 124923 | FJ900608 | FJ900635 | FJ900616 |
| *N. batangarum* | CBS 124924 | FJ900607 | FJ900634 | FJ900615 |
| *N. batangarum* | CPC 29624 | MT587474 | MT592679 | MT592376 |
| *N. illicii* | BJFU 2037 | KY350149 | KY350155 | - |
| *N. illicii* | BJFU 2038 | KY350150 | KY350156 | - |
| *N. occulatum* | CBS 128008 | EU301030 | EU339472 | EU339558 |
| *N. occulatum* | CBS 519.74 | MT587499 | MT592706 | - |
| *N. occulatum* | CPC 32162 | MT587500 | MT592707 | MT592404 |
| *N. sinoeucalypti* | CERC2005 | KX278061 | KX278270 | KX278290 |
| *N. sinoeucalypti* | CERC3415 | KX278063 | KX278272 | KX278292 |
| *N. parvum* | CBS 112879 | AY343474 | MT592643 | MT592339 |
| *N. parvum* | CBS 161.29 | MT587450 | MT592650 | MT592346 |
| *N. parvum* | CCF109 | KC507812 | KC507806 | KC507803 |
| *N. parvum* | CERC3509 | KX278057 | KX278266 | KX278286 |
| *N. pandanicola* | CBS 118832 | MT587501 | MT592708 | - |
| *B. parva* | ATCC 58191 | AY236943 | AY236917 | EU821963 |
| *N. algeriense* | CBS 137504 | KJ657702 | KX505915 | - |
| *N. algeriense* | CAA322 | KX505906 | KX505916 | - |
| *N. algeriense* | CBS 119937 | MT587444 | MT592642 | MT592338 |
| *N. italicum* | CBS 140889 | MT587479 | MT592684 | - |
| *N. algeriense* | CBS 719.85 | KX464151 | KX464921 | KX464000 |
| *N. podocarpi* | CBS 115065 | MT587507 | MT592714 | MT592411 |
| *N. podocarpi* | CBS 131677 | MT587508 | MT592715 | MT592412 |
| *N. brasiliense* | CMM 1285 | JX513628 | KC794030 | - |
| *N. brasiliense* | CMM 1338 | JX513630 | KC794031 | - |
| *N. cordaticola* | CBS 123634 | EU821898 | EU821838 | EU821928 |
| *N. cordaticola* | CBS 123635 | EU821903 | EU821843 | EU821933 |
| *N. hongkongense* | CERC 2967 | KX278050 | KX278259 | KX278281 |
| *N. hongkongense* | CERC 2968 | KX278051 | KX278260 | KX278282 |
| *N. hongkongense* | CERC 2973 | KX278052 | KX278261 | KX278283 |
| *L. theobromae* | CBS306.58 | EF622071 | MT592638 | MT592334 |

# **Table S2** Reference strains and GenBank accession numbers for *RPB2* sequence used in phylogenetic analysis of *Fusarium solani* species complex**.**

| **Species** | **Fungal Code**^2^ | ***RPB2*** |
| --- | --- | --- |
| *F. petroliphilum* | CBS 135955 | KJ867426 |
| *F. oblongum* | NRRL 28008 | EF470135 |
| *F. cyanescens* | NRRL 37625 | EU329637 |
| *F. ferrugineum* | NRRL 32437 | EU329581 |
| *F. bostrycoides* | NRRL 31169 | EU329564 |
| *F. waltergamsii* | NRRL 32323 | EU329576 |
| *F. vanettenii* | NRRL 22278 | EU329501 |
| *F. vanettenii* | CBS 123669 | KM232364 |
| *F. vanettenii* | NRRL 45880 | JX171655 |
| *F. solani* | NRRL 43468 | EF469980 |
| *F. solani* | NRRL 43474 | EF469984 |
| *F. suttonianum* | NRRL 32858 | EU329630 |
| *F. falciforme* | CBS 47567 | LT960558 |
| *F. quercinum* | NRRL 22652 | EU329518 |
| *F. neocosmosporiellum* | NRRL 22166 | EU329497 |
| *F. protoensiforme* | NRRL 22178 | EU329498 |
| *F. riograndense* | CMF 12570 | KX534003 |
| *F. ambrosium* | NRRL 20438 | JX171584 |
| *F. euwallaceae* | NRRL 54726 | JQ038032 |
| *F. virguliforme* | NRRL 31041 | JX171643 |
| *F. brasiliense* | NRRL 31757 | EU329565 |
| *F. cuneirostrum* | NRRL 31157 | FJ240389 |
| *F. phaseoli* | NRRL 22276 | JX171608 |
| *F. verticillioides* | LC18464 | OQ126649 |

# **Table S3** Reference strains and GenBank accession numbers *RPB2* sequences used in phylogenetic analysis of *Fusarium fujikuroi* species complex.

| **Species** | **Fungal Code**^3^ | ***RPB2*** |
| --- | --- | --- |
| *F. sacchari* | LC13679 | MW474492 |
| *F. sacchari* | LC13678 | MW474491 |
| *F. sacchari* | NRRL 13999 | JX171580 |
| *F. sacchari* | LC13657 | MW474457 |
| *F. sacchari* | LC13626 | MW474411 |
| *F. erosum* | LC15877 | OQ126518 |
| *F. globosum* | CBS 120992 | MW402788 |
| *F. globosum* | CBS 430.97 | MN534265 |
| *F. fujikuroi* | CBS 119855 | MW402735 |
| *F. fujikuroi* | CBS 265.54 | MN534268 |
| *F. fujikuroi* | NRRL 13566 | JX171570 |
| *F. pseudoanthophilum* | CBS 414.97 | MT010980 |
| *F. brevicatenulatum* | CBS 404.97 | MN534295 |
| *F. planum* | LC15876 | OQ126555 |
| *F. verticillioides* | LC18464 | OQ126649 |
| *F. verticillioides* | LC18525 | OQ126583 |
| *F. verticillioides* | CBS 218.76 | MW928835 |
| *F. verticillioides* | LC2810 | MW474453 |
| *F. mundagurra* | LGS129.3 | MZ399209 |
| *F. mundagurra* | LGS129.2 | MZ399208 |
| *F. mundagurra* | LC13689 | MW474502 |
| *F. xyrophilum* | NRRL 62721 | MN193905 |
| *F. xyrophilum* | NRRL 62710 | MN193903 |
| *F. denticulatum* | CBS 407.97 | MT010970 |
| *F. nygamai* | CBS 413.97 | MW402815 |
| *F. nygamai* | NRRL 13448 | EF470114 |
| *F. pseudograminearum* | NRRL 28062 | JX171637 |

# **Table S4** Reference strains and GenBank accession numbers for *ITS* sequences used in phylogenetic analysis of *Gilbertella* spp.

| **Species** | **Fungal Code**^4^ | ***ITS*** |
| --- | --- | --- |
| *Choanephora cucurbitacearum* | CBS 178.76 | JN206235 |
| *Choanephora cucurbitacearum* | CBS 674.93 | JN206233 |
| *Choanephora cucurbitacearum* | CBS 155.58 | JN206238 |
| *Poitrasia circinans* | CBS 153.58 | MH857733 |
| *Poitrasia circinans* | CBS 647.70 | JN206240 |
| *Blakeslea trispora* | CBS 198.80 | JN206228 |
| *Blakeslea trispora* | CBS 130.59 | JN206227 |
| *Blakeslea trispora* | CBS 137.49 | JN206229 |
| *Gilbertella persicaria* | F216057 | MK301176 |
| *Gilbertella persicaria* | CBS 247.59 | MH855278 |
| *Gilbertella persicaria* | CBS 325.71A | JN206220 |
| *Gilbertella persicaria* | CBS 442.64 | JN206219 |
| *Gilbertella persicaria* | CBS 403.51 | JN206221 |
| *Gilbertella persicaria* | CBS 785.97 | JN206218 |
| *Mucor fuscus* | CBS 282.78 | JN206201 |

# **Table S5** Reference strains and GenBank accession numbers for *ITS* sequences used in phylogenetic analysis of *Mucor* spp.

| **Species** | **Fungal Code**^5^ | ***ITS*** |
| --- | --- | --- |
| *Mucor circinelloides* | CBS 108.16 | JN205954 |
| *Mucor circinelloides* | CBS 384.95 | MH862534 |
| *Mucor circinelloides* | CBS 416.77 | JN205934 |
| *Mucor ctenidius* | CBS 293.66 | MH858796 |
| *Mucor harpali* | CNUFC ICT18001 | MT192528 |
| *Mucor plumbeus* | CBS 634.74 | HM999955 |
| *Mucor racemosus* | CBS 115.08 | JN205919 |
| *Mucor amethystinus* | CBS 526.68 | JN206015 |
| *Mucor amethystinus* | CBS 846.73 | JN206014 |
| *Mucor pseudolusitanicus* | CBS 543.80 | MF495060.1 |
| *Mucor pseudolusitanicus* | CBS 540.78 | MF495059 |
| *Mucor lusitanicus* | CBS 108.17 | JN205980 |
| *Mucor variicolumellatus* | SF012536 | MF495054.1 |
| *Mucor phayaoensis* | MFLUCC 21-0043 | MZ379498 |
| *Mucor variicolumellatus* | CBS 236.35 | JN205979 |
| *Mucor pseudocircinelloides* | CBS 541.78 | JN206013.1 |
| *Mucor exponens* | CBS 141.20 | MH854686 |
| *Mucor fuscus* | CBS 132.22 | JF723619 |
| *Mucor fuscus* | CBS 230.29 | JN206204 |
| *Mucor abundans* | CBS 521.66 | JN206110 |
| *Mucor abundans* | CBS 388.35 | JN206111 |
| *Mucor parviseptatus* | CBS 417.77 | JN206108 |
| *Mucor hiemalis* | CBS 249.35 | JN206122 |
| *Mucor hiemalis* | CBS 412.71 | JN206124 |
| *Mucor rongii* | CICC 41725 | MK903014 |
| *Mucor aligarensis* | NNIBRFG6255 | MN267431 |
| *Mucor flavus* | CBS 230.35 | JN206061 |
| *Backusella dispersa* | CBS 195.28 | JN206271 |

# **Table S6** Reference strains and GenBank accession numbers for calmodulin gene (*cmd*) sequences used in phylogenetic analysis of *Aspergillus* section *Flavi*.

| **Species** | **Fungal Code**^6^ | ***cmd*** |
| --- | --- | --- |
| *Aspergillus aflatoxiformans* | CBS 121.62 | MG518089 |
| *Aspergillus aflatoxiformans* | DTO 087-A2 | MG517990 |
| *Aspergillus aflatoxiformans* | CBS 143679 | MG518076 |
| *Aspergillus austwickii* | CBS 143677T | MG518072 |
| *Aspergillus austwickii* | DTO 228-F9 | MG518074 |
| *Aspergillus pipericola* | CBS 143680T | MG518087 |
| *Aspergillus minisclerotigenes* | CBS 117635T | MG518009 |
| *Aspergillus minisclerotigenes* | CBS 117633 | MG518007 |
| *Aspergillus flavus* | CBS 110.55 | MG518005 |
| *Aspergillus flavus* | COAD 3307 | MZ467046 |
| *Aspergillus flavus* | CBS 501.65 | MG518015 |
| *Aspergillus flavus* | CBS 569.65 | EF661508 |
| *Aspergillus flavus* | CBS 542.69 | MG518016 |
| *Aspergillus cerealis* | CBS 143674 | MG518063 |
| *Aspergillus cerealis* | CBS 143676 | MG518065 |
| *Aspergillus parasiticus* | CBS 100926 | EF661516 |
| *Aspergillus parasiticus* | CBS 260.67 | MG518013 |
| *Aspergillus sojae* | CBS 133.52 | EF661517 |
| *Aspergillus novoparasiticus* | CBS 126849 | MG518055 |
| *Aspergillus novoparasiticus* | CBS 126850 | MG518057 |
| *Aspergillus sergii* | CBS 130017 | MG518059 |
| *Aspergillus arachidicola* | CBS 117610 | EF202049 |
| *Aspergillus arachidicola* | DTO 228-H9 | MG518091 |
| *Aspergillus mottae* | CBS 130016 | MG518058 |
| *Aspergillus tamarii* | CBS 104.13T | EF661526 |

# **Table S7** Reference strains and GenBank accession numbers for *ITS* sequences used in phylogenetic analysis of *Talaromyces* spp.

| **Species** | **Fungal Code**^7^ | ***ITS*** |
| --- | --- | --- |
| *Talaromyces funiculosus* | CBS 272.86 | JN899377 |
| *Talaromyces macrosporus* | CBS 317.63 | JN899333 |
| *Talaromyces galapagensis* | CBS 751.74 | JN899358 |
| *Talaromyces purpureogenus* | CBS 286.36 | JN899372 |
| *Talaromyces argentinensis* | NRRL 28750 | MH793045 |
| *Talaromyces versatilis* | CBS 140377 | KC962111 |
| *Talaromyces versatilis* | AS3.15853 | MK837960 |
| *Talaromyces sparsus* | AS3.16003 | MT077182 |
| *Talaromyces sparsus* | AS3.15880 | MK837958 |
| *Talaromyces panamensis* | CBS 128.89 | JN899362 |
| *Talaromyces xishaensis* | AS3.17995 | KU644580 |
| *Talaromyces stollii* | CBS 408.93 | JX315674 |
| *Talaromyces amestolkiae* | CBS 132696 | JX315660 |
| *Talaromyces ruber* | CBS 132704 | JX315662 |
| *Talaromyces derxii* | CBS 412.89 | JN899327 |
| *Talaromyces fusiformis* | CBS 140637 | KU866656 |
| *Talaromyces rubicundus* | CBS 342.59 | JN899384 |
| *Talaromyces duclauxii* | CBS 322.48 | JN899342 |
| *Talaromyces marneffei* | CBS 388.87 | JN899344 |
| *Talaromyces qii* | CBS 139515 | KP765384 |
| *Talaromyces aureolinus* | AS3.15865 | MK837953 |
| *Talaromyces aureolinus* | AS3.16004 | MN059095 |
| *Talaromyces dendriticus* | CBS 660.80 | JN899339 |

# **Table S8** Reference strains and GenBank accession numbers for *ITS* sequences used in phylogenetic analysis of *Aspergillus* section *Terrei*.

| **Species** | **Fungal Code**^8^ | ***ITS*** |
| --- | --- | --- |
| *Aspergillus ardalensis* | CCF CZE:4031 | FR733808 |
| *Aspergillus capensis* | CBS 138188 | KJ775550 |
| *Aspergillus capensis* | CanS-34 | MK072769 |
| *Aspergillus urmiensis* | CBS 139557 | KP987072 |
| *Aspergillus templicola* | CBS 138180 | KP987081 |
| *Aspergillus neoflavipes* | NRRL 5504 | EF669614 |
| *Aspergillus micronesiensis* | CBS 138183 | KJ775548 |
| *Aspergillus polyporicola* | NRRL 32683 | EF669595 |
| *Aspergillus spelaeus* | CCF 4425 | HG915905 |
| *Aspergillus luppii* | NRRL 6326 | EF669617 |
| *Aspergillus movilensis* | CCF 4410 | KP987089 |
| *Aspergillus aureoterreus* | NRRL 1923 | EF669580 |
| *Aspergillus terreus* | NRRL 2399 | EF669585 |
| *Aspergillus terreus* | NRRL 255 | EF669586 |
| *Aspergillus pseudoterreus* | NRRL 4017 | EF669598 |
| *Aspergillus niveus* | NRRL 5505 | EF669615 |
| *Aspergillus allahabadii* | NRRL 4539 | EF669601 |
| *Aspergillus neoindicus* | NRRL 6134 | EF669616 |
| *Aspergillus carneus* | NRRL 527 | EF669611 |
| *Aspergillus iranicus* | CBS 139560 | KP987076 |
| *Aspergillus flavus* | COAD 3307 | MZ467046 |

**
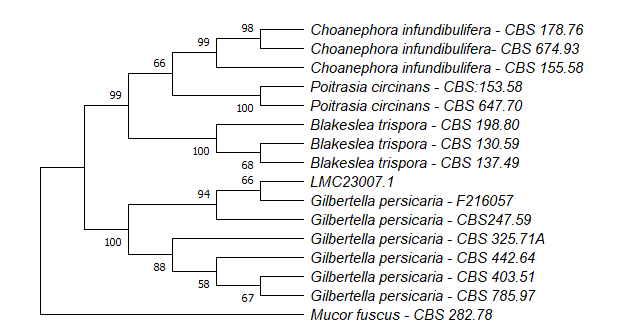
**

# **Fig. S3** Phylogenetic tree of the *Gilbertella* sp. Maximum Likelihood phylogenetic tree based on *ITS* sequences confirming the identity of LMC23007.1 as *Gilbertella persicaria*. Bootstrap support values (1,000 replicates) are indicated at the nodes. Reference sequences were retrieved from GenBank, with species names and corresponding strain codes provided.

**
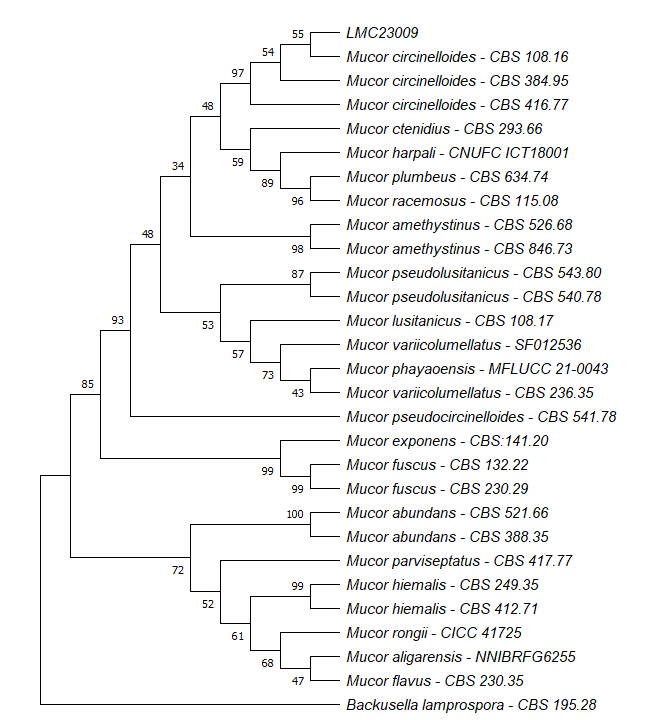
**

# **Fig. S4** Phylogenetic tree of the *Mucor* spp. Maximum Likelihood phylogenetic tree based on *ITS* sequences confirming the identity of LMC23009 as *Mucor circillenoides*. Bootstrap support values (1,000 replicates) are indicated at the nodes. Reference sequences were retrieved from GenBank, with species names and corresponding strain codes provided.

**
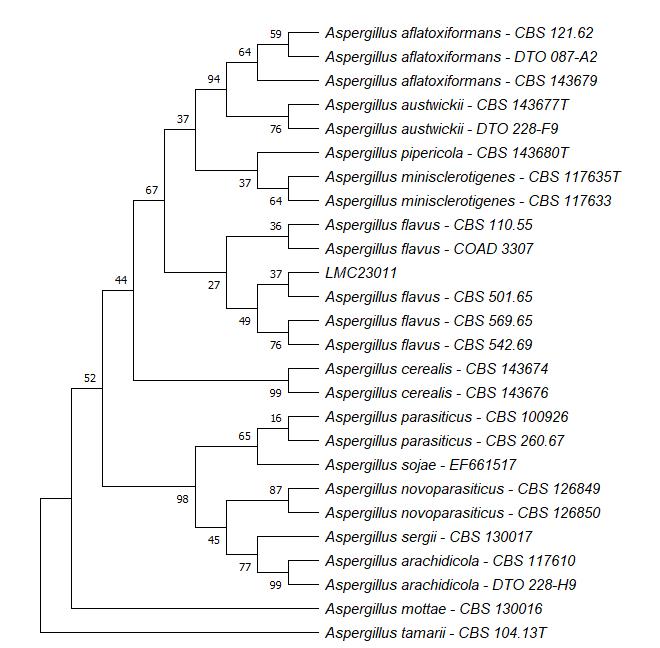
**

# **Fig. S5** Phylogenetic tree of *Aspergillus* section *Flavi*. Maximum Likelihood phylogenetic tree based on *cmd* sequences showing the placement of isolate LMC23011 within *Aspergillus* section *Flavi* as *A. flavus*. Bootstrap support values (1,000 replicates) are indicated at the nodes. Reference sequences were retrieved from GenBank, with species names and corresponding strain codes provided.

**
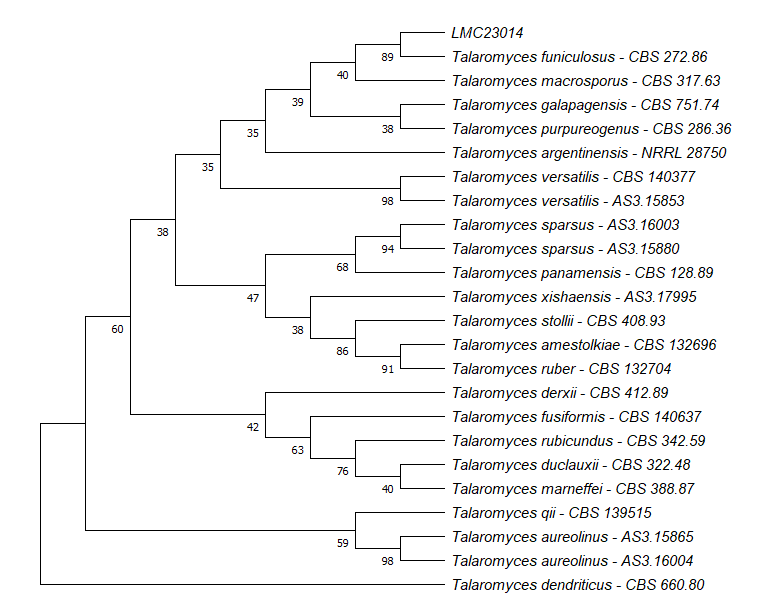
**

# **Fig. S6** Phylogenetic tree of the *Talaromyces* spp. Maximum Likelihood phylogenetic tree based on *ITS* sequences confirming the identity of isolate LMC23014 as *Talaromyces funiculosum*. Bootstrap support values (1,000 replicates) are indicated at the nodes. Reference sequences were retrieved from GenBank, with species names and corresponding strain codes provided.

**
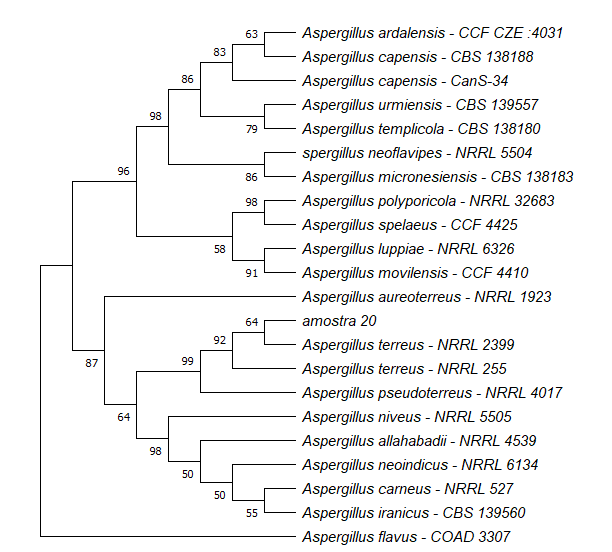
**

# **Fig. S7** Phylogenetic tree of the *Aspergillus* section *Terrei*. Maximum Likelihood phylogenetic tree based on *ITS* sequences showing the placement of isolate LMC23020 within *Aspergillus* section *Terrei* as *A. terreus*. Bootstrap support values (1,000 replicates) are indicated at the nodes. Reference sequences were retrieved from GenBank, with species names and corresponding strain codes provided.

# **Table S9** The GNPS2 workflow establishes connections between fungi, their growth conditions, and the resulting annotated compounds.

| **Workflow for sample LMC23007.2 cultivated in rice and corn.** | | | |
| --- | --- | --- | --- |
| **Compound name** | **Scan** | **Cosine** | **Spectrum ID** |
| Adenosine | 31 | 0.98 | CCMSLIB00005777992 |
| Fusaric acid | 110 | 0.84 | CCMSLIB00012431618 |
| Isomarticin | 440 | 0.82 | CCMSLIB00005727416 |
| Cyclosporin A | 1162 | 0.82 | CCMSLIB00000531481 |
| Sansalvamide | 1323 | 0.88 | CCMSLIB00000577669 |
| N-methylsansalvamide | 1345 | 0.78 | CCMSLIB00000577589 |
| 2,4-Tetradecadienedioic acid, 12-[[13-carboxy-3-[(13-carboxy-12,14-dihydroxy-3,5,7-trimethyl-1-oxo-2,4-tetradecadien-1-yl)oxy]-2-(hydroxymethyl)-8,10,12-trimethyl-1-oxo-10,12-tridecadien-1-yl]oxy]-13-(hydroxymethyl)-3,5,7-trimethyl | 1358 | 0.72 | CCMSLIB00004692771 |
| Task ID: https://gnps2.org/status?task=8dc6addc172e42c8b5c6e04bc915b5db | | | |
|  |  |  |  |
| **Workflow for sample LMC23008 cultivated in rice and corn.** | | | |
| **Compound name** | **Scan** | **Cosine** | **Spectrum ID** |
| 7-Hydroxy-3-(2-hydroxy-propyl)-5-methyl-epiisochromen-1-one | 169 | 0.86 | CCMSLIB00000845283 |
| Isomarticin | 345 | 0.88 | CCMSLIB00005727416 |
| NG-391 | 388 | 0.81 | CCMSLIB00000855001 |
| Marticin | 400 | 0.88 | CCMSLIB00005727416 |
| Task ID: https://gnps2.org/status?task=04c85b2030df4111af5c48a4bba82953 | | | |
|  |  |  |  |
| **Workflow for sample LMC23012 cultivated in rice, corn, and SCB.** | | | |
| **Compound name** | **Scan** | **Cosine** | **Spectrum ID** |
| 7,19-Dihydroxy-6,18-epoxykaur-16-en-18-one | 705 | 0.70 | CCMSLIB00000850819 |
| Oxysporidinone | 1484 | 0.73 | CCMSLIB00000478099 |
| Beauvericin D | 1634 | 0.77 | CCMSLIB00000577656 |
| Beauvericin-A | 1754 | 0.92 | CCMSLIB00000577681 |
| Task ID: https://gnps2.org/status?task=988d359aac2248188cfb5a0a1bbe9b47 | | | |
|  |  |  |  |
| **Workflow for sample LMC23015 cultivated in rice and corn.** | | | |
| **Compound name** | **Scan** | **Cosine** | **Spectrum ID** |
| Fusarin C | 747 | 0.73 | CCMSLIB00000074973 |
| epi-fusarin C | 794 | 0.75 | CCMSLIB00000074972 |
| Farnesol | 780 | 0.80 | CCMSLIB00003138783 |
| Beauvericin J | 1190 | 0.85 | CCMSLIB00000577814 |
| Beauvericin-A | 1237 | 0.75 | CCMSLIB00000478099 |
| Dimethyl ketal of oxysporidinone | 1410 | 0.90 | CCMSLIB00000478100 |
| Task ID: https://gnps2.org/status?task=0c3c8f72d8df4400b9a7ebc9495a804a | | | |
|  |  |  |  |
| **Workflow for sample LMC23018 cultivated in rice, corn, wheat.** | | | |
| **Compound name** | **Scan** | **Cosine** | **Spectrum ID** |
| Fumonisin B1 | 655 | 0.84 | CCMSLIB00005772889 |
| Fumonisin B3 | 841 | 0.79 | CCMSLIB00005775284 |
| Fumonisin B2 | 1035 | 0.90 | CCMSLIB00005775284 |
| Fumonisin A3 | 1535 | 0.78 | CCMSLIB00000845809 |
| Fumonisin A2 | 1685 | 0.79 | CCMSLIB00000845809 |
| 1,2,3-Propanetricarboxylic acid, 2-(acetyloxy)-, 1,2,3-tributyl ester | 2278 | 0.88 | CCMSLIB00005760659 |
| 1β-Hydroxycholic acid | 2396 | 0.92 | CCMSLIB00005465783 |
| Dioseptemloside B | 2493 | 0.77 | CCMSLIB00016039038 |
| Filipin II | 2508 | 0.85 | CCMSLIB00016037987 |
| 26-deoxylaidlomycin | 2521 | 0.78 | CCMSLIB00016032678 |
| 1-Linoleoylglycerol | 2723 | 0.79 | CCMSLIB00003134936 |
| 2,6,10,34-Hexatriacontatetraene-1,15,19,23,27,31-hexol, 3,7,11,15,19,23,27,31,35-nonamethyl- | 2778 | 0.80 | CCMSLIB00000847470 |
| Ergosterol peroxide | 2893 | 0.77 | CCMSLIB00000848042 |
| Task ID: https://gnps2.org/status?task=e184226052024a368d997da15a99fcbc | | | |
|  |  |  |  |
| **Workflow for sample LMC23006 cultivated in rice, corn, and SCB.** | | | |
| **Compound name** | **Scan** | **Cosine** | **Spectrum ID** |
| 6-Hydroxy-4-methylcoumarin | 270 | 0.75 | CCMSLIB00000205234 |
| 4,7-dihydroxymellein | 339 | 0.76 | CCMSLIB00000478501 |
| -Hydroximellein | 411 | 0.76 | CCMSLIB00000478502 |
| Hymecromone | 410 | 0.77 | CCMSLIB00000205234 |
| 4-Hydroximellein | 501 | 0.77 | CCMSLIB00000478502 |
| O-guaiacylglycerol | 939 | 0.85 | CCMSLIB00005748687 |
| Luminmide B | 1342 | 0.86 | CCMSLIB00001059263 |
| Beauvericin | 1562 | 0.92 | CCMSLIB00005723573 |
| Monoolein | 1847 | 0.79 | CCMSLIB00003138956 |
| Task ID: https://gnps2.org/status?task=35d545d8fb1e4ed088f5fbaac185328f | | | |
|  |  |  |  |
| **Workflow for smaple F1 cultivated in rice and corn.** | | | |
| **Compound name** | **Scan** | **Cosine** | **Spectrum ID** |
| 2-Methyl-3-carboxy-1,2,3,4-tetrahydroharman | 119 | 0.85 | CCMSLIB00010117759 |
| 1,2,3,4-Tetrahydro-3-carboxyharmane | 170 | 0.87 | CCMSLIB00010117759 |
| 2-(2-Furanyl)-4-thiazoleacetic acid | 215 | 0.88 | CCMSLIB00012877491 |
| Fusaric acid | 257 | 0.83 | CCMSLIB00012431618 |
| Task ID: https://gnps2.org/status?task=b5dc37f034df438db6eaa5f4ae51a68e | | | |
|  |  |  |  |
| **Workflow for samples LMC23007.2, LMC23008, LMC23012, LMC23015, LMC23018, F1, LMC23006 cultivated in rice and corn.** | | | |
| **Compound name** | **Scan** | **Cosine** | **Spectrum ID** |
| Austidiol | 1415 | 0.79 | CCMSLIB00012436853 |
| Oxysporidinone | 5676 | 0.74 | CCMSLIB00000478099 |
| Task ID: https://gnps2.org/status?task=1b441c15e7154e79b3c90610c8a4e01d | | | |

# **Table S10** Annotated compounds from the molecular networks and those isolated from the LMC23007.2 fungus.

| **Compound** | **Node** | **Retention**  **Time (min)** | **Name** | **Formula** | ***m/z*** | **Adduct** | **Error**  **(ppm)** | **Annotation**  **Method** |
| --- | --- | --- | --- | --- | --- | --- | --- | --- |
| **1** | 31 | 1.74 | Adenosine | C₁₀H₁₃N₅O₄ | 268.1029 | M+H | -5.6 | I,G |
| **2** | 43 | 2.48 | (R)-fusarinolic acid | C₁₀H₁₃NO₃ | 196.0967 | M+H | -3.6 | I |
| **3** | 68 | 5.76 | 9,10-Dehydrofusaric acid | C₁₀H₁₁NO₂ | 178.0856 | M+H | -6.7 | I |
| **4** | 110 | 7.86 | Fusaric acid | C₁₀H₁₃NO₂ | 180.1009 | M+H | -8.3 | I,G, F^9^ |
| **5** | 155 | 9.42 | Methyl 5-[(3*S*)-3-hydroxybutyl]-2-pyridinecarboxylater | C₁₁H₁₅NO₃ | 210.1122 | M+H | -3.8 | S |
| **6** | 162 | 9.61 | Gibepyrone D | C₁₀H₁₀O₄ | 195.068 | M+H | 11.8 | S, F^10^ |
| **7** | 301 | 12.79 | Aloesol | C₁₃H₁₄O₄ | 235.0953 | M+H | -7.2 | S, F^10^ |
| **8** | 340 | 13.51 | Methyl dehydrofusarate | C₁₁H₁₃NO₂ | 192.1019 | M+H | -2.6 | S, F^11^ |
| **9** | 387 | 14.12 | Anhydrofusarubin | C₁₅H₁₂O₆ | 289.0713 | M+H | 0.3 | S, F^10^ |
| **10** | 388 | 14.3 | 9-*O*-Demethylherbarin | C₁₅H₁₄O₆ | 291.0855 | M+H | -4.8 | S |
| **11** | 440 | 15.23 | Isomarticin | C₁₈H₁₆O₉ | 377.0892 | M+H | 5.0 | G, F^12^ |
| **12** | 475 | 15.55 | Calonectrin | C₁₉H₂₆O₆ | 351.1797 | M+H | -2.7 | S |
| **13** | 533 | 16.03 | Benzoic acid, 2-[(6*S*,8*R*)-6,8-dihydroxynonyl]-4,6-dihydroxy-, ethyl ester | C₁₈H₂₈O₆ | 363.1783 | M+Na | -0.3 | S, F^13^ |
| **14** | 532 | 16.05 | Fusarilactone A | C₁₈H₂₆O₅ | 345.1667 | M+Na | -3.2 | CW,S |
| **15** | 540 | 16.08 | Marticin | C₁₈H₁₆O₉ | 377.0902 | M+H | 7.7 | CW, F^12^ |
| **16** | 592 | 16.48 | Fusaridioic acid A | C₁₈H₃₀O₆ | 343.2107 | M+H | -4.0 | S, F^14^ |
| **17** | 663 | 16.87 | Isomarticin methyl ester | C₁₉H₁₈O₉ | 391.1021 | M+H | -2.0 | S, F^15^ |
| **18** | 711 | 17.21 | 12-Hydroxy-13-(hydroxymethyl)-3,5,7-trimethyl-2-tetradecenedioic acid | C₁₈H₃₂O₆ | 345.2219 | M+H | -16.8 | CW,S |
| **19** | 709 | 17.23 | Metoxi fusaridioic acid A | C₁₉H₃₂O₆ | 379.21 | M+Na | 1.0 | S |
| **20** | 837 | 18.24 | Hymeglusin | C₁₈H₂₈O₅ | 347.1829 | M+Na | -1.4 | CW, F^16^ |
| **21** | 942 | 18.96 | Antroquinonol T | C₂₆H₄₂O₈ | 505.2774 | M+Na | -0.6 | S |
| **22** | 1083 | 19.95 | Halymecin C | C₃₂H₅₈O₁₁ | 619.4022 | M+Na | -5.6 | S, F^17^ |
| **23** | 1122 | 20.18 | Cyclosporin A metabolite 17 | C₆₂H₁₁₁N₁₁O₁₃ | 1218.8365 | M+H | -6.2 | S |
| **24** | 1166 | 20.4 | Fusariumester A1 | C₃₆H₅₈O₁₁ | 667.4048 | M+H | -1.5 | CW,S, F^18^ |
| **25** | 1162 | 20.44 | Cyclosporin A | C₆₂H₁₁₁N₁₁O₁₂ | 1202.8463 | M+H | -2.4 | G |
| **26** | 1165 | 20.6 | Fusariumester B | C₃₆H₅₆O₁₀ | 649.3945 | M+H | -1.0 | CW,F^18^ |
| **27** | 1205 | 20.79 | Pupukeamide | C₃₄H₅₆N₄O₇ | 655.4043 | M+Na | -0.4 | S |
| **28** | 1246 | 21.03 | Fusariumester A2 | C₃₆H₅₈O₁₁ | 667.4031 | M+H | -4.0 | CW,S, F^18^ |
| **29** | 1323 | 21.69 | Sansalvamide | C₃₂H₅₀N₄O₆ | 587.3802 | M+H | -1.0 | CW, G, F^19^ |
| **30** | 1345 | 21.92 | N-methylsansalvamide | C₃₃H₅₂N₄O₆ | 601.3976 | M+H | 1.8 | CW,G, F^20^ |
| **31** | 1358 | 22.03 | 2,4-Tetradecadienedioic acid, 12-[[13-carboxy-3-[(13-carboxy-12,14-dihydroxy-3,5,7-trimethyl-1-oxo-2,4-tetradecadien-1-yl)oxy]-2-(hydroxymethyl)-8,10,12-trimethyl-1-oxo-10,12-tridecadien-1-yl]oxy]-13-(hydroxymethyl)-3,5,7-trimethyl | C₅₄H₈₆O₁₆ | 991.5939 | M+Na | -5.5 | G |
| **32** | 1372 | 22.13 | Rhodopeptin B5 | C₂₈H₅₃N₅O₄ | 546.399 | M+Na | -0.9 | S |
| **33** | 1480 | 22.82 | Cyclosporin C | C₆₂H₁₁₁N₁₁O₁₃ | 1218.8417 | M+H | -2.0 | CW, S |
| **34** | 1512 | 23.2 | Cyclosporin B | C₆₁H₁₀₉N₁₁O₁₂ | 1188.8318 | M+H | -1.5 | CW, S |

**Legend:** The codes represent: I for isolated and identified compounds; F for *Fusarium* isolates; CW for ChemWalk annotations; S for Sirius-processed data; and G for GNPS-derived data.

# **Table S11** Annotated compounds from the molecular networks and those isolated from the LMC23008 fungus

| **Compound** | **Node** | **Retention Time (min)** | **Name** | **Formula** | ***m/z*** | **Adduct** | **Error**  **(ppm)** | **Annotation**  **Method** |
| --- | --- | --- | --- | --- | --- | --- | --- | --- |
| **35** | 56 | 7.99 | Dihydrofusarubin | C₁₅H₁₆O₇ | 331.0781 | M+Na | -3.4 | I |
| **36** | 109 | 9.7 | Citreoisocoumarin | C₁₄H₁₄O₆ | 279.0865 | M+H | -1.4 | S,CW, F^21^ |
| **37** | 169 | 11 | 7-Hydroxy-3-(2-hydroxypropyl)-5-methyl-1*H*-2-benzopyran-1-one | C₁₃H₁₄O₄ | 235.0963 | M+H | -3.0 | I,F^22^ |
| **38** | 302 | 14.23 | Fusarubin | C₁₅H₁₄O₇ | 307.0813 | M+H | -1.6 | I,F^10^ |
| **11** | 345 | 15.24 | Isomarticin | C₁₈H₁₆O₉ | 377.0852 | M+H | -5.6 | G, F^12^ |
| **39** | 356 | 15.38 | Fusarin PM1 | C₂₂H₂₇NO₇ | 418.2 | M+H | -6.2 | CW,F |
| **40** | 489 | 16.72 | 5,7,10-Trihydroxy-3-methylbenz[*g*]isoquinoline-6,9-dione | C₁₄H₉NO₅ | 272.0533 | M+H | -9.6 | I |
| **41** | 388 | 15.85 | NG-391 | C₂₂H₂₇NO₇ | 418.1822 | M+H | -10.5 | G,F^23^ |
| **15** | 400 | 16.09 | Marticin | C₁₈H₁₆O₉ | 377.0851 | M+H | -5.8 | CW, F^12^ |
| **42** | 443 | 16.28 | Lucilactaene | C₂₂H₂₇NO₆ | 402.187 | M+H | -11.7 | CW,F^23^ |
| **43** | 480 | 16.56 | Bostrycoidin | C₁₅H₁₁NO₅ | 286.0695 | M+H | -7.3 | I^10^ |
| **44** | 524 | 16.99 | Fusarin F | C₂₃H₂₉NO₇ | 432.1977 | M+H | -10.4 | CW,F^24^ |
| **45** | 542 | 17.04 | Chaetiacandin | C₄₃H₆₀O₁₆ | 871.3485 | M+K | -3.8 | S |
| **46** | 536 | 17.06 | NG 393 (Isomer of **41**) | C₂₂H₂₇NO₇ | 418.1822 | M+H | -10.5 | CW F^25^ |
| **47** | 551 | 17.15 | Fusarin A | C₂₃H₂₉NO₆ | 416.2008 | M+H | -15.6 | CW,F^24^ |
| **48** | 577 | 17.38 | isomero de 443 | C₂₂H₂₇NO₆ | 402.1875 | M+H | -10.4 | CW |
| **49** | 601 | 17.56 | 3-O-methylfusarubin | C₁₆H₁₆O₇ | 343.076 | M+Na | -9.6 | I,F^10^ |
| **50** | 606 | 17.6 | 5-deoxybostrycoidin | C₁₅H₁₁NO₄ | 270.0733 | M+H | -12.2 | I,F^26^ |
| **51** | 609 | 17.65 | 8-acetylneosolaniol | C₂₁H₂₈O₉ | 425.1771 | M+H | -0.9 | S, F^27^ |
| **52** | 618 | 17.68 | 7-O-Demethylscorpinone | C₁₅H₁₁NO₄ | 270.0747 | M+H | -7.0 | S |
| **53** | 1015 | 23.58 | Unguisin B | C₃₇H₅₆N₈O₇ | 725.4353 | M+H | 0.4 | S |

**Legend:** The codes represent: I for isolated and identified compounds; F for *Fusarium* isolates; CW for ChemWalk annotations; S for Sirius-processed data; and G for GNPS-derived data.

# **Table S12** Annotated compounds from the molecular networks and those isolated from the LMC23012 fungus.

| **Compound** | **Node** | **Retention Time (min)** | **Name** | **Formula** | ***m/*z** | **Adduct** | **Error (ppm)** | **Annotation Method** |  |  |
| --- | --- | --- | --- | --- | --- | --- | --- | --- | --- | --- |
|  |  |  |  |  |  |  |  |  |  |  |
| **2** | 56 | 2.44 | Fusarinolic acid | C₁₀H₁₃NO₃ | 196.0966 | M+H | -4.1 | I |  |  |
| **54** | 628 | 13.7 | Bassiatin | C₁₅H₁₉NO₃ | 262.1435 | M+H | -3.1 | S,F^28^ |  |  |
| **55** | 705 | 14.56 | 7,18-Dihydroxykaurenolide | C₂₀H₂₈O₄ | 333.2053 | M+H | -7.5 | G,S, F^29^ |  |  |
| **43** | 855 | 16.56 | Bostrycoidin | C₁₅H₁₁NO₅ | 286.0729 | M+H | -5.9 | I |  |  |
| **56** | 1082 | 18.9 | Versicoumarin C | C₁₆H₂₀O₄ | 277.1431 | M+H | -3.6 | CW |  |  |
| **57** | 1167 | 19.76 | *7-but-15-enyl-6,8-dihydroxy-3(*R*)-penta-9,11-dienylisocoumarin* | C₁₈H₂₀O₄ | 301.1393 | M+H | -1.0 | I |  |  |
| **58** | 1191 | 19.84 | 7-but-2-enyl-6,8-dihydroxy-3-pent-3-enyl-3,4-dihydroisochromen-1-one | C₁₈H₂₂O₄ | 303.1589 | M+H | -2.3 | I |  |  |
| **59** | 1316 | 20.58 | 7-Butyl-6,8-dihydroxy-3(R)-pent-11-enylisochroman-1-one | C₁₈H₂₄O₄ | 305.1736 | M+Na | -5.6 | I |  |  |
| **60** | 1334 | 20.77 | Oxysporidinone derivative | C₂₈H₄₅NO₆ | 492.3275 | M+H | -10.2 | CW,S |  |  |
| **61** | 1349 | 20.87 | Fusapyrone | C₃₄H₅₄O₉ | 607.3799 | M+H | -7.6 | S, F^30^ |  |  |
| **62** | 1390 | 21.1 | Stylissamide E | C₃₉H₅₈N₈O₉ | 783.4486 | M+H | 10.3 | S, CW |  |  |
| **63** | 1412 | 21.22 | Beauvericin J | C₄₅H₅₇N₃O₁₀ | 800.4096 | M+H | -3.2 | S |  |  |
| **64** | 1484 | 21.61 | Oxysporidinone | C₂₈H₄₃NO₆ | 490.3135 | M+H | -6.9 | G, F^31^ |  |  |
| **65** | 1537 | 21.88 | Beauvenniatin H3 | C₃₈H₅₉N₃O₉ | 702.4288 | M+H | -6.0 | CW, S |  |  |
| **66** | 1575 | 22.05 | Beauvenniatin A | C₄₁H₅₇N₃O₉ | 736.4168 | M+H | -0.7 | CW,S, F^32^ |  |  |
| **67** | 1609 | 22.14 | Enniatin A, 2-(*N*-methyl-L-phenylalanine)- (ZCI) | C₃₉H₆₁N₃O₉ | 716.4481 | M+H | -0.7 | CW |  |  |
| **68** | 1634 | 22.2 | Beauvericin D | C₄₄H₅₅N₃O₉ | 770.4022 | M+Na | 0.6 | G,CW, F^21^ |  |  |
| **69** | 1649 | 22.24 | Beauvericin K | C₄₂H₅₉N₃O₉ | 772.4134 | M+Na | -1.9 | CW, F^33^ |  |  |
| **70** | 1652 | 22.26 | Beauvericin | C₄₅H₅₇N₃O₉ | 806.3982 | M+Na | -1.2 | I |  |  |
| **71** | 1754 | 22.76 | Beauvericin A | C₄₆H₅₉N₃O₉ | 798.4315 | M+H | -1.7 | G,CW, F^34^ |  |  |

**Legend:** The codes represent: I for isolated and identified compounds; F for *Fusarium* isolates; CW for ChemWalk annotations; S for Sirius-processed data; and G for GNPS-derived data.


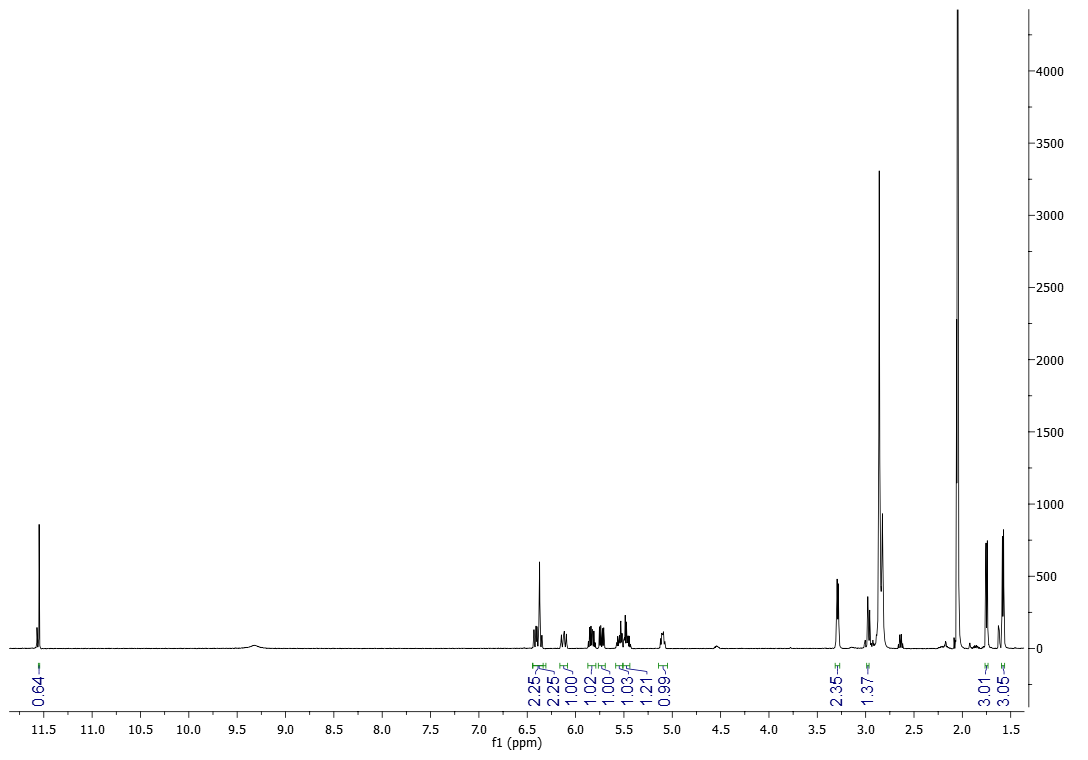


# **Fig. S8** ¹H NMR data for compound **57** in acetone-*d*₆ (500 MHz).


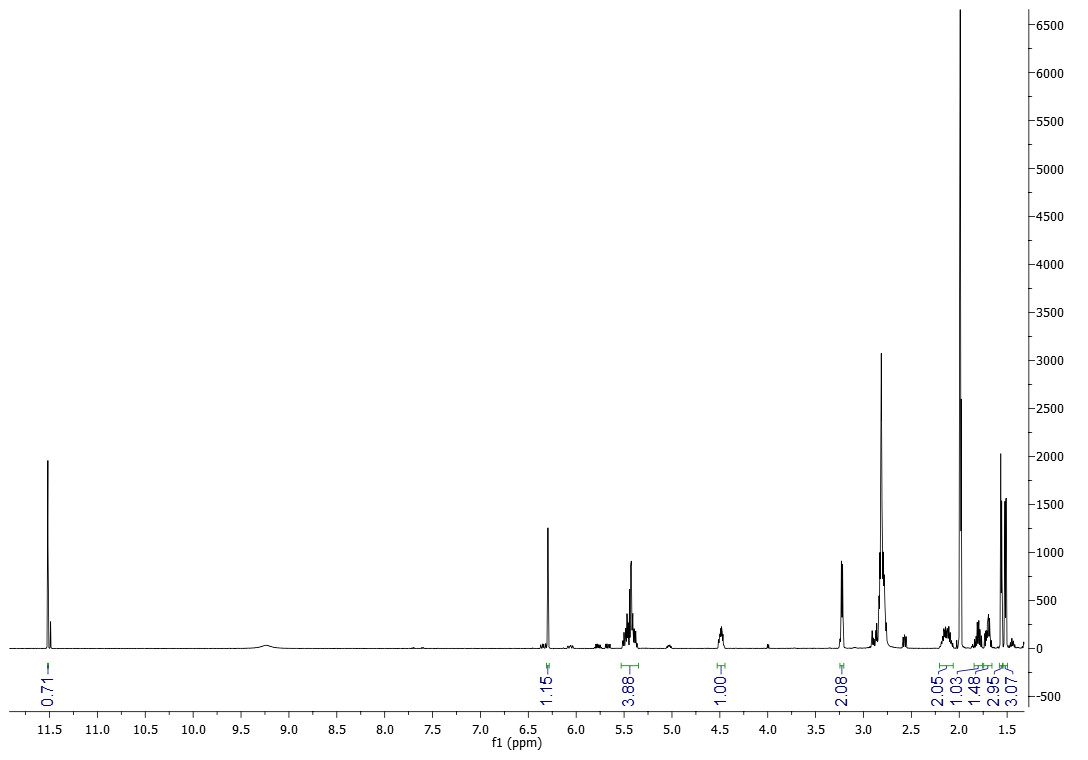


# **Fig. S9** ¹H NMR data for compound **58** in acetone-*d*₆ (500 MHz).

**
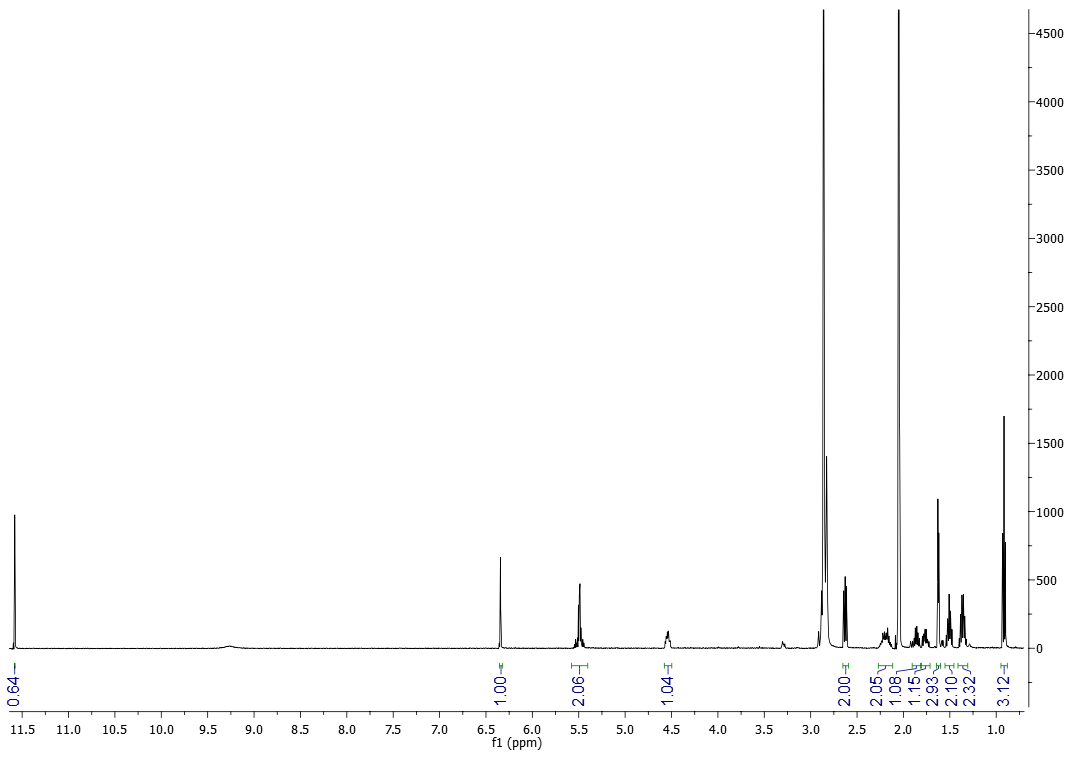
**

# **Fig. S10** ¹H NMR data for compound **59** in acetone-*d*₆ (500 MHz).

# **Table S13** Annotated compounds from the molecular networks and those isolated from the LMC23015 fungus.

| **Compound** | **Node** | **Retention Time (min)** | **Name** | **Formula** | ***m/z*** | **Adduct** | **Error(ppm)** | **Annotation Method** |  |  |
| --- | --- | --- | --- | --- | --- | --- | --- | --- | --- | --- |
|  |  |  |  |  |  |  |  |  |  |  |
| **72** | 31 | 1.56 | 5-(3,4-Dihydroxybutyl)-2-pyridinecarboxylic acid | C₁₀H₁₃NO₄ | 212.0941 | M+H | 8.5 | I |  |  |
| **73** | 50 | 2.45 | 10-hydroxyfusaric acid | C₁₀H₁₃NO₃ | 196.0977 | M+H | 1.5 | I |  |  |
| **74** | 121 | 6.63 | 2-Pyridinecarboxylic acid, 5-[3-(acetyloxy)butyl] | C₁₂H₁₅NO₄ | 238.1079 | M+H | 8.8 | I |  |  |
| **75** | 236 | 9.35 | Dimethyl 3,3'-(pyrazine-2,5-diyl)dipropanoate | C₁₂H₁₆N₂O₄ | 253.1175 | M+H | -5.1 | S |  |  |
| **76** | 240 | 9.42 | Methyl 5-(4-hydroxybutyl)-2-pyridinecarboxylate | C₁₁H₁₅NO₃ | 210.1126 | M+H | -1.9 | CW |  |  |
| **47** | 712 | 17.08 | Fusarin A | C₂₃H₂₉NO₆ | 416.2044 | M+H | 7.0 | S |  |  |
| **77** | 780 | 17.56 | Farnesol | C₁₅H₂₆O | 223.203 | M+H | -14.3 | G |  |  |
| **78** | 770 | 17.56 | Cyclonerodiol | C₁₅H₂₈O₂ | 263.1957 | M+Na | -11.4 | I,F ^9^ |  |  |
| **62** | 1181 | 21.13 | Stylissamide E | C₃₉H₅₈N₈O₉ | 783.4454 | M+H | 6.2 | CW |  |  |
| **63** | 1190 | 21.25 | Beauvericin J | C₄₅H₅₇N₃O₁₀ | 800.4092 | M+H | -3.7 | G |  |  |
| **64** | 1237 | 21.63 | Oxysporidinone | C₂₈H₄₃NO₆ | 490.3147 | M+H | -4.5 | G |  |  |
| **66** | 1287 | 22.06 | Beauvenniatin A | C₄₁H₅₇N₃O₉ | 736.4131 | M+H | -5.7 | CW |  |  |
| **69** | 1342 | 22.25 | Beauvericin K | C₄₂H₅₉N₃O₉ | 750.4357 | M+H | 3.7 | CW |  |  |
| **79** | 1354 | 22.31 | (-)-6-Deoxyoxysporidinone | C₂₈H₄₃NO₅ | 474.3166 | M+H | -11.2 | CW |  |  |
| **70** | 1365 | 22.39 | Beauvericin | C₄₅H₅₇N₃O₉ | 806.3952 | M+Na | -5.0 | I |  |  |
| **80** | 1410 | 22.57 | Dimethyl ketal of oxysporidinone | C₃₀H₄₉NO₇ | 558.3352 | M+Na | -9.8 | G |  |  |

**Legend:** The codes represent: **I** for isolated and identified compounds; **F** for *Fusarium* isolates; **CW** for ChemWalk annotations; **S** for Sirius-processed data; and **G** for GNPS-derived data.

# **Table S14** Annotated compounds from the molecular networks and those isolated from the LMC23018 fungus.

| **Compound** | **Node** | **Retention Time (min)** | **Name** | **Formula** | ***m/z*** | **Adduct** | **Error (ppm)** | **Annotation Method** |  |
| --- | --- | --- | --- | --- | --- | --- | --- | --- | --- |
|  |  |  |  |  |  |  |  |  |  |
| **2** or **73** | 38 | 2.43 | hydroxyfusaric acid | C₁₀H₁₃NO₃ | 196.097 | M+H | -2.0 | I |  |
| **4** | 106 | 7.87 | fusaric acid | C₁₀H₁₃NO₂ | 180.1016 | M+H | -4.4 | I |  |
| **5** | 191 | 9.4 | Fusarinolic acid methyl ester | C₁₁H₁₅NO₃ | 210.1133 | M+H | 1.4 | S |  |
| **81** | 308 | 11.52 | 6-hydroxy-6-(4-methoxy-2-oxo-2H-pyran-6-yl)-2,4-dimethylhexanoic acid | C₁₄H₂₀O₆ | 285.1323 | M+H | -5.3 | I |  |
| **82** | 518 | 13.72 | Desacylfumonisin B_1_ | C₂₂H₄₇NO₅ | 406.3476 | M+H | -13.8 | S, F^35^ |  |
| **83** | 593 | 14.4 | Fumonisin PH1b | C₂₈H₅₃NO₁₀ | 564.3675 | M+H | -12.9 | S, F^36^ |  |
| **84** | 617 | 14.6 | 2-[2-(19-amino-11,17,18-trihydroxy-5,9-dimethyl-6-oxoicosan-7-yl)oxy-2-oxoethyl]butanedioic acid | C₂₈H₅₁NO₁₀ | 562.3554 | M+H | -6.6 | S |  |
| **85** | 655 | 14.95 | Fumonisin B1 | C₃₄H₅₉NO₁₅ | 722.394 | M+H | -3.0 | G, F^37^ |  |
| **86** | 750 | 15.62 | Thermolide B | C₂₈H₅₁NO₉ | 546.3575 | M+H | 2.0 | S,CW |  |
| **87** | 808 | 16.04 | Hydrolyzed Fumonisin B_3_ | C₂₂H₄₇NO₄ | 390.3525 | M+H | -14.8 | S |  |
| **88** | 841 | 16.19 | Fumonisin B_3_ | C₃₄H₅₉NO₁₄ | 706.3997 | M+H | -2.3 | G, F^35^ |  |
| **89** | 856 | 16.26 | Thermolide C | C₂₈H₅₁NO₉ | 546.3586 | M+H | 4.0 | S,CW |  |
| **90** | 919 | 16.52 | Thermolide A | C₂₈H₅₁NO₉ | 546.3621 | M+H | 10.4 | S,CW |  |
| **91** | 1002 | 16.85 | Flavensomycin | C₄₇H₆₅NO₁₄ | 868.4513 | M+H | 3.5 | S |  |
| **92** | 1035 | 16.98 | Fumonisin B_2_ | C₃₄H₅₉NO₁₄ | 706.4015 | M+H | 0.3 | G, F^38^ |  |
| **93** | 1094 | 17.19 | Fumonisin A_1_ | C₃₆H₆₁NO₁₆ | 764.4065 | M+H | -0.5 | S, F^39^ |  |
| **94** | 1107 | 17.21 | 1,2,3-Propanetricarboxylic acid, 1-(13-(acetylamino)-5,10,12-trihydroxy-3-methyl-1-(2-methyl-1-oxohexyl)tetradecyl) ester | C₃₀H₅₃NO₁₁ | 604.3725 | M+H | 4.6 | S, CW |  |
| **95** | 1179 | 17.53 | Fumonisin C_4_ | C₃₃H₅₇NO₁₃ | 676.3916 | M+H | 1.2 | S, F^40^ |  |
| **96** | 1234 | 17.67 | Thermolide E | C₂₈H₅₃NO₈ | 532.3826 | M+H | -4.3 | CW |  |
| **97** | 1336 | 18.02 | Fumonisin B_4_ | C₃₄H₅₉NO₁₃ | 690.4042 | M+H | -3.2 | S, F^41^ |  |
| **98** | 1420 | 18.31 | 2-[[(5R,6R,7S,9S,11R,18R,19S)-19-amino-6-(3,4-dicarboxybutanoyloxy)-11 ,18-dihydroxy-5,9-dimethyl-icosan-7-yl]oxycarbonylmethyl]butanedioic a cid | C₃₅H₆₁NO₁₃ | 704.4215 | M+H | -0.8 | S |  |
| **99** | 1483 | 18.5 | Fellutamide F | C₂₈H₅₃N₅O₈ | 588.3892 | M+H | -13.4 | S,CW |  |
| **100** | 1535 | 18.69 | Fumonisin A3 | C₃₆H₆₁NO₁₅ | 748.4147 | M+H | 3.6 | G, F^42^ |  |
| **101** | 1685 | 19.13 | Fumonisin A2 | C₃₆H₆₁NO₁₅ | 748.4139 | M+H | 2.5 | G, F^42^ |  |
| **102** | 1736 | 19.35 | 1,2,3-Propanetricarboxylic acid, 1-[13-(acetylamino)-10,12-dihydroxy-3-methyl-1-(2-methyl-1-oxohexyl)tetradecyl] ester | C₃₀H₅₃NO₁₀ | 588.3754 | M+H | 1.0 | G |  |
| **103** | 1768 | 19.44 | Fumonisin AK1 | C₃₁H₅₅NO₁₀ | 602.3899 | M+H | -0.8 | S |  |
| **104** | 2278 | 21.25 | 1,2,3-Propanetricarboxylic acid, 2-(acetyloxy)-, 1,2,3-tributyl ester | C₂₀H₃₄O₈ | 403.2324 | M+H | -2.0 | G |  |
| **105** | 2321 | 21.43 | Beauverolide L | C₂₉H₄₅N₃O₅ | 516.3489 | M+H | 10.1 | S, CW |  |
| **106** | 2396 | 21.68 | 1β-Hydroxycholic acid | C₂₄H₄₀O₆ | 871.5509 | 2M+Na | -4.4 | G |  |
| **107** | 2432 | 21.92 | Glycocholic acid | C₂₆H₄₃NO₆ | 466.3164 | M+H | -1.1 | S |  |
| **108** | 2493 | 22.15 | Dioseptemloside B | C₃₃H₅₄O₉ | 595.3814 | M+H | -5.4 | G |  |
| **109** | 2508 | 22.18 | Filipin II | C₃₅H₅₈O₁₀ | 639.4071 | M+H | -5.8 | G |  |
| **110** | 2521 | 22.22 | 26-deoxylaidlomycin | C₃₇H₆₂O₁₁ | 683.4326 | M+H | -6.4 | G |  |
| **111** | 2723 | 22.98 | 1-Linoleoylglycerol | C₂₁H₃₈O₄ | 355.2827 | M+H | -5.9 | G |  |
| **112** | 2778 | 23.34 | 2,6,10,34-Hexatriacontatetraene-1,15,19,23,27,31-hexol, 3,7,11,15,19,23,27,31,35-nonamethyl- | C₄₅H₈₄O₆ | 721.6322 | M+H | -3.3 | G |  |
| **113** | 2791 | 23.43 | Bifurcatriol | C₂₀H₃₆O₃ | 325.2697 | M+H | -14.1 | S, CW |  |
| **114** | 2813 | 23.57 | 9-Octadecynoic acid | C₁₈H₃₂O₂ | 281.2435 | M+H | -16.0 | S, CW |  |
| **115** | 2827 | 23.61 | Monoolein | C₂₁H₄₀O₄ | 357.2978 | M+H | -5.9 | S, CW |  |
| **116** | 2893 | 24.16 | Ergosterol peroxide | C₂₈H₄₄O₃ | 411.3215 | [M-H2O]+H | -11.7 | G |  |
| **117** | 2942 | 24.63 | Protothecasterol | C₂₈H₄₂O | 395.3264 | M+H | -12.6 | S |  |

**Legend:** The codes represent: **I** for isolated and identified compounds; **F** for *Fusarium* isolates; **CW** for ChemWalk annotations; **S** for Sirius-processed data; and **G** for GNPS-derived data


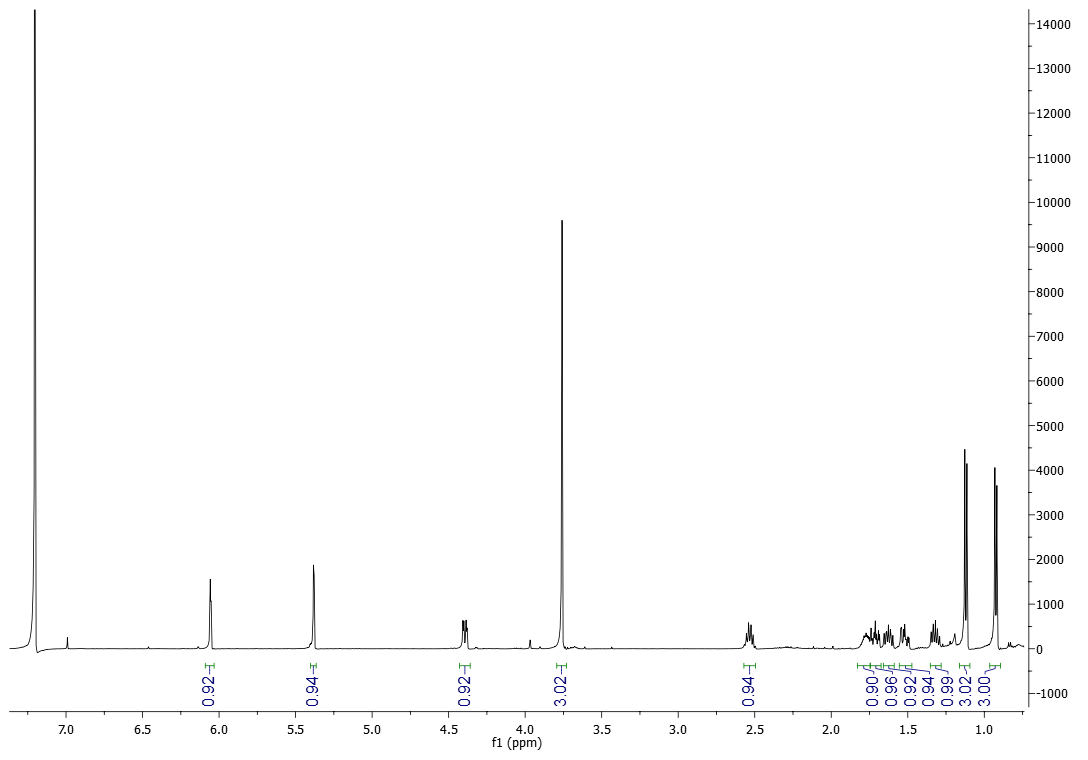


# **Fig. S11** ¹H NMR data for compound **81** in CDCl_3_ (500 MHz).


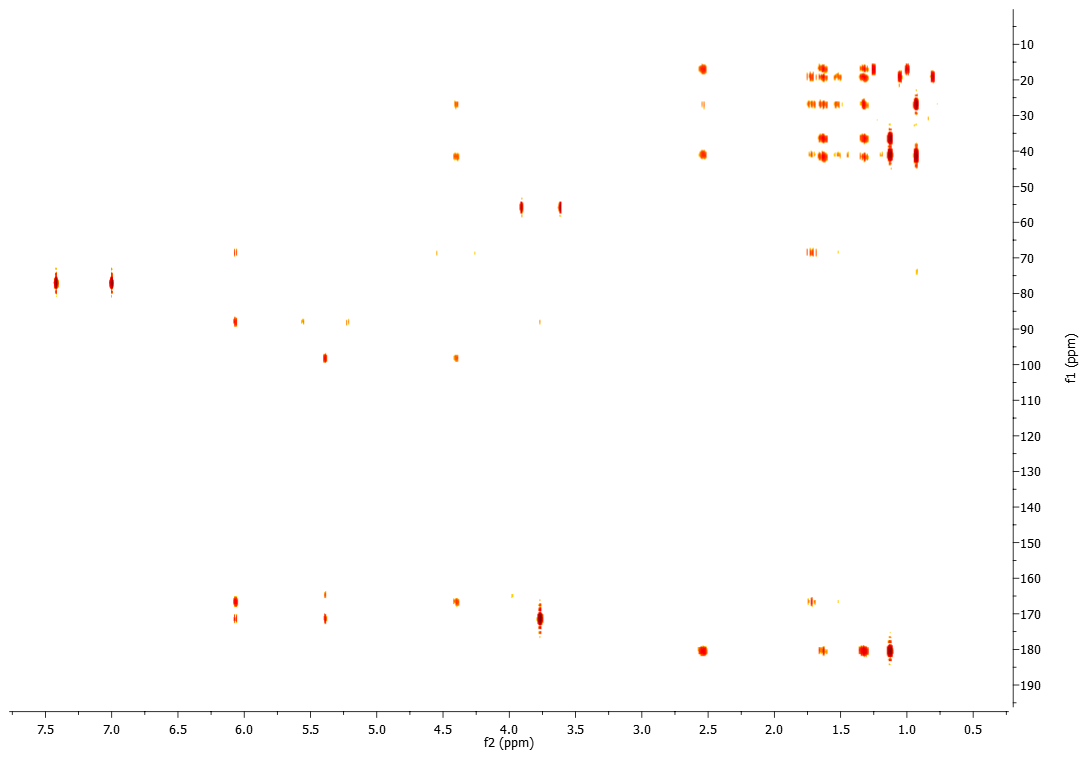


# **Fig. S12** HMBC correlations for compound **81** in CDCl₃ (500 MHz).


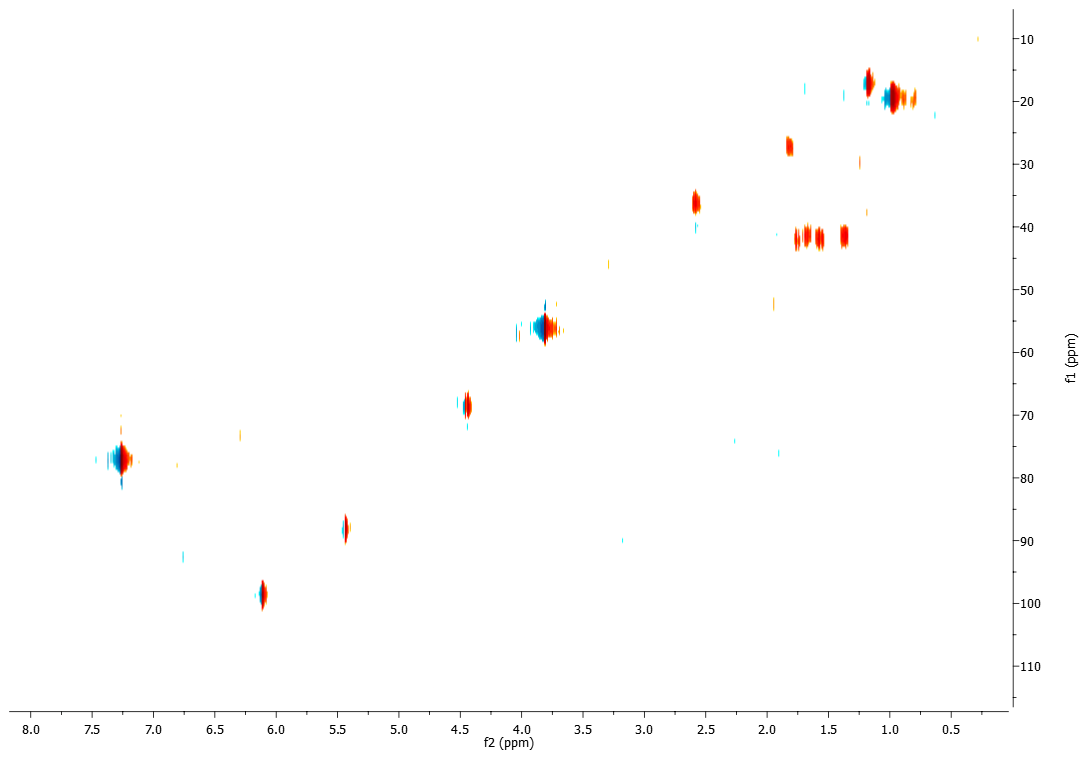


# **Fig. S13** HSQC spectrum of compound **81** in CDCl₃ (500 MHz).

# **Table S15** Annotated compounds from the molecular networks and those isolated from the F1 fungus

| **Compound** | **Node** | **Retention Time (min)** | **Name** | **Formula** | ***m/z*** | **Adduct** | **Error (ppm)** | **Annotation**  **Method** |  |
| --- | --- | --- | --- | --- | --- | --- | --- | --- | --- |
|  |  |  |  |  |  |  |  |  |  |
| **2** | 62 | 2.45 | (R)-fusarinolic acid | C₁₀H₁₃NO₃ | 196.0976 | M+H | 1.0 | I, F^9^ |  |
| **118** | 119 | 5.09 | 2-Methyl-3-carboxy-1,2,3,4-tetrahydroharman | C₁₃H₁₄N₂O₂ | 231.1113 | M+H | -1.3 | G, S |  |
| **3** | 161 | 5.71 | 9,10-Dehydrofusaric acid | C₁₀H₁₁NO₂ | 178.0858 | M+H | -5.6 | I^9^ |  |
| **119** | 170 | 5.91 | 1,2,3,4-Tetrahydro-3-carboxyharmane | C₁₃H₁₄N₂O₂ | 231.112 | M+H | -5.2 | G,S |  |
| **120** | 215 | 6.81 | 2-(2-Furanyl)-4-thiazoleacetic acid | C₉H₇NO₃S | 210.0227 | M+H | -0.9 | G,S |  |
| **4** | 257 | 7.67 | Fusaric acid | C₁₀H₁₃NO₂ | 180.1028 | M+H | 2.2 | I, G, S, F^9^ |  |
| **121** | 264 | 7.75 | 3-Ethyl-2,5-pyrazinedipropanoic acid | C₁₂H₁₆N₂O₄ | 253.1175 | M+H | -5.1 | S |  |
| **122** | 333 | 9.12 | Fusarpyrone A | C₁₀H₁₂O₃ | 181.0868 | M+H | 1.6 | CW, F^43^ |  |
| **75** | 346 | 9.34 | Dimethyl 3,3'-(pyrazine-2,5-diyl)dipropanoate | C₁₂H₁₆N₂O₄ | 253.1178 | M+H | -4.0 | S |  |
| **123** | 349 | 9.44 | Fusarinolic acid methyl ester | C₁₁H₁₅NO₃ | 210.1114 | M+H | -7.6 | S |  |
| **8** | 560 | 13.51 | Methyl dehydrofusarate | C₁₁H₁₃NO₂ | 192.1014 | M+H | -5.2 | CW, F^11^ |  |
| **124** | 627 | 15.22 | 5-azulenemethanol, 1,3a,4,5,6,8a-hexahydro-α,α,3,8-tetramethyl- | C₁₅H₂₄O | 221.1882 | M+H | -10.4 | S, CW |  |
| **125** | 632 | 15.25 | Fusaric acid methyl ester | C₁₁H₁₅NO₂ | 194.1164 | M+H | -8.7 | CW, F^11^ |  |
| **126** | 636 | 15.35 | Fusagerin C | C₁₃H₁₈N₂O₃ | 251.1399 | M+H | 1.2 | CW, F^44^ |  |
| **127** | 647 | 15.65 | 7-isopropenyl-1,4-dimethyl-3a,5,6,7,8,8a-hexahydro-3H-azulen-4-ol | C₁₅H₂₄O | 221.1886 | M+H | -8.6 | S, CW |  |
| **128** | 657 | 15.73 | Hydroxyfungerin A | C₁₃H₁₈N₂O₃ | 251.1368 | M+H | -11.1 | CW |  |
| **129** | 665 | 15.85 | Cladosporin | C₂₂H₂₄N₂O₅S₂ | 483.0987 | M+Na | -7.7 | I |  |
| **130** | 699 | 16.31 | Haematocin | C₂₄H₂₆N₂O₆S₂ | 525.1094 | M+Na | -6.9 | I, S |  |
| **131** | 767 | 17.56 | 3-Caryolen-5-ol | C₁₅H₂₄O | 221.1879 | M+H | -11.8 | S, CW |  |
| **132** | 776 | 17.57 | Guaia-6,10(14)-diene | C₁₅H₂₄ | 205.1941 | M+H | -7.3 | CW, F^45^ |  |
| **78** | 777 | 17.59 | Cyclonerodiol | C₁₅H₂₈O₂ | 263.1948 | M+Na |  | I, F^9^ |  |
| **133** | 944 | 20.79 | Norcoumingide | C₂₈H₄₅NO₆ | 492.3263 | M+H | -12.6 | CW |  |
| **134** | 946 | 20.82 | Aristolochene | C₁₅H₂₄ | 205.1937 | M+H | -9.2 | CW |  |
| **64** | 1016 | 21.6 | Oxysporidinone | C₂₈H₄₃NO₆ | 490.3102 | M+H | -13.7 | G |  |

**Legend:** The codes represent: **I** for isolated and identified compounds; **F** for *Fusarium* isolates; **CW** for ChemWalk annotations; **S** for Sirius-processed data; and **G** for GNPS-derived data.

# **Table S16** Annotated compounds from the molecular networks and those isolated from the LMC23006 fungus

| **Compound** | **Node** | **Retention Time (min)** | **Name** | **Formula** | ***m/z*** | **Adduct** | **Error (ppm)** | **Annotation Method** |  |  |
| --- | --- | --- | --- | --- | --- | --- | --- | --- | --- | --- |
|  |  |  |  |  |  |  |  |  |  |  |
| **135** | 118 | 3.08 | Asperlinol | C₁₀H₁₄O₆ | 231.0851 | M+H | -7.4 | CW |  |  |
| **136** | 150 | 3.98 | isomer Asperlinol | C₁₀H₁₄O₆ | 231.0859 | M+H | -3.9 | CW |  |  |
| **137** | 227 | 5.38 | Asperlin | C₁₀H₁₂O₅ | 213.0765 | M+H | 8.0 | I |  |  |
| **138** | 241 | 5.43 | Asperochrin F | C₁₀H₁₀O₅ | 211.0614 | M+Na | 3.9 | CW |  |  |
| **139** | 258 | 5.7 | Rhizosolaniol | C₁₂H₁₀O₆ | 273.0367 | M+Na | -2.9 | I |  |  |
| **140** | 270 | 5.71 | 6-Hydroxy-4-methylcoumarin | C₁₀H₈O₃ | 177.0560 | M+H | 4.5 | G,S |  |  |
| **141** | 273 | 5.8 | 6-Hydroximellein | C₁₀H₁₀O₄ | 195.0666 | M+H | 4.6 | S, CW |  |  |
| **142** | 302 | 6.16 | 4,6,8-trihydroxy-3-methyl-3,4-dihydroisochromen-1-one | C₁₀H₁₀O₅ | 211.0618 | M+H | 5.7 | S, CW |  |  |
| **143** | 339 | 6.66 | 4,7-dihydroxymellein | C₁₀H₁₀O₅ | 211.0614 | M+H | 3.8 | G |  |  |
| **144** | 411 | 7.47 | 4-Hydroximellein | C₁₀H₁₀O₄ | 195.0663 | M+H | 3.1 | G |  |  |
| **145** | 410 | 7.48 | Hymecromone | C₁₀H₈O₃ | 177.0547 | M+H | -2.8 | G, S |  |  |
| **146** | 501 | 8.42 | 4-Hydroximellein | C₁₀H₁₀O₄ | 195.0666 | M+H | 4.6 | G |  |  |
| **147** | 528 | 8.83 | Furan-2-carboxylic anhydride | C₁₀H₆O₅ | 207.0301 | M+H | 3.4 | I |  |  |
| **148** | 539 | 8.94 | 7-Hydroxy-4-methylcoumarin-3-acetic acid | C₁₂H₁₀O₅ | 235.0600 | M+H | -2.6 | S |  |  |
| **149** | 590 | 9.61 | 3-hydroxymethyl-8-hydroxyl-3,4-dihydroisocoumarin | C₁₀H₁₀O₄ | 195.0670 | M+H | 6.7 | S, CW |  |  |
| **150** | 599 | 9.65 | 4-Methyldaphnetin | C₁₀H₈O₄ | 193.0493 | M+H | -4.1 | S, CW |  |  |
| **151** | 935 | 14.85 | Quercetin 3,3′-dimethyl ether | C₁₇H₁₄O₇ | 331.0817 | M+H | -0.3 | S |  |  |
| **152** | 939 | 14.9 | O-guaiacylglycerol | C₂₇H₂₆O₁₁ | 527.1536 | M+H | -3.2 | G |  |  |
| **153** | 1126 | 18.85 | Terpestacin | C₂₅H₃₈O₄ | 403.2814 | M+H | -8.5 | CW |  |  |
| **154** | 1185 | 19.7 | Fusaproliferin | C₂₇H₄₀O₅ | 467.2756 | M+Na | -3.6 | CW |  |  |
| **155** | 1342 | 21.36 | Luminmide B | C₂₉H₅₃N₅O₅ | 552.4091 | M+H | -6.1 | G,S |  |  |
| **156** | 1385 | 21.54 | Clavatustide C | C₃₀H₅₅N₅O₅ | 566.4245 | M+H | -6.3 | S, CW |  |  |
| **157** | 1436 | 21.89 | cyclo[DL-Leu-DL-Leu-DL-Leu-DL-Leu-DL-xiIle] | C₃₀H₅₅N₅O₅ | 566.4269 | M+H | -2.1 | S, CW |  |  |
| **70** | 1562 | 22.36 | Beauvericin | C₄₅H₅₇N₃O₉ | 806.3944 | M+Na | -5,6 | I |  |  |
| **115** | 1847 | 23.61 | Monoolein | C₂₁H₄₀O₄ | 357.2971 | M+H | -9.5 | G, S |  |  |

**Legend:** The codes represent: **I** for isolated and identified compounds; **CW** for ChemWalk annotations; **S** for Sirius-processed data; and **G** for GNPS-derived data.

# **Table S17** Annotated compounds from the molecular networks and those isolated from the coculture.

| **Compound** | **Node** | **Retention Time (min)** | **Name** | **Formula** | ***m/z*** | **Adduct** | **Error (ppm)** | **Annotation Method** |  |  |
| --- | --- | --- | --- | --- | --- | --- | --- | --- | --- | --- |
|  |  |  |  |  |  |  |  |  |  |  |
| **137** | 337 | 5.4 | Asperlin | C₁₀H₁₂O₅ | 213.0767 | M+H | 1.9 | I |  |  |
| **158** | 453 | 6.15 | 1*H*-2-Benzopyran-1-one, 3,4-dihydro-4,6,8-trihydroxy-3-methy**l** | C₁₀H₁₀O₅ | 211.0607 | M+H | 0.5 | S, CW |  |  |
| **159** | 507 | 6.51 | 1*H*-2-Benzopyran-1-one, 3,4-dihydro-4,5,8-trihydroxy-3-methyl- | C₁₀H₁₀O₄ | 195.0672 | M+H | 7.7 | S, CW |  |  |
| **148** | 932 | 8.94 | 7-Hydroxy-4-methylcoumarin-3-acetic acid | C₁₂H₁₀O₅ | 235.0605 | M+H | -0.5 | S, CW |  |  |
| **160** | 1091 | 9.71 | Peniisocoumarin G | C₁₂H₁₂O₆ | 253.0729 | M+H | 6.7 | S, CW |  |  |
| **161** | 1209 | 10.24 | Benzenepropanal, *O*-β-D-galactopyranosyloxime | C₁₅H₂₁NO₆ | 312.142 | M+H | -8.6 | S, CW |  |  |
| **162** | 1401 | 11.08 | *O*-Acetylerucifoline *N*-oxide | C₂₀H₂₅NO₈ | 408.1594 | M+H | -9.8 | S, CW |  |  |
| **163** | 1415 | 11.11 | Austidiol | C₁₂H₁₂O₅ | 237.0795 | M+H | 6.3 | G |  |  |
| **164** | 1422 | 11.14 | 8-Ethoxy-3-oxoretrorsine | C₂₀H₂₇NO₈ | 410.1772 | M+H | -10.5 | S, CW |  |  |
| **165** | 1784 | 12.58 | Riddelline | C₁₈H₂₃NO₆ | 350.1588 | M+H | -4.6 | CW |  |  |
| **166** | 1894 | 13 | Jacozine | C₁₈H₂₃NO₆ | 350.1597 | M+H | -2.0 | S, CW |  |  |
| **167** | 2184 | 14.11 | 2-Butenoic acid, 2-(hydroxymethyl)-, [2,3,5,7a-tetrahydro-1-[(3-methyl-1-oxo-2-butenyl)oxy]-4-oxido-1*H*-pyrrolizin-7-yl]methyl ester, [1*R*-[1α,7(*Z*),7aβ]]- (9CI) | C₁₈H₂₅NO₆ | 352.1762 | M+H | 0.6 | S, CW |  |  |
| **168** | 2303 | 14.51 | Doriasenine | C₁₈H₂₅NO₆ | 352.1743 | M+H | -4.8 | S, CW |  |  |
| **169** | 2456 | 15.08 | Latifoline | C₂₀H₂₇NO₇ | 394.1857 | M+H | -2.3 | S, CW |  |  |
| **170** | 5089 | 20.35 | Andrastin A | C₂₈H₃₈O₇ | 487.2644 | M+H | -10.8 | S |  |  |
| **171** | 5553 | 21.41 | Citreohybridone G | C₂₉H₄₀O₇ | 501.2773 | M+H | -15.6 | S |  |  |
| **64** | 5676 | 21.62 | Oxysporidinone | C₂₈H₄₃NO₆ | 490.3139 | M+H | -6.1 | G |  |  |
| **172** | 6039 | 22.24 | *N*-Demethylsambutoxin | C₂₇H₃₇NO₄ | 440.2775 | M+H | -5.9 | S |  |  |
| **173** | 6215 | 22.56 | Sambutoxin | C₂₈H₃₉NO₄ | 454.2946 | M+H | -2.4 | S |  |  |

**Legend:** The codes represent: **I** for isolated and identified compounds; **F** for *Fusarium* isolates; **CW** for ChemWalk annotations; **S** for Sirius-processed data; and **G** for GNPS-derived data.

# **References**

1. Zhang W, Groenewald JZ, Lombard L, Schumacher RK, Phillips AJL, Crous PW. Evaluating species in Botryosphaeriales. Persoonia. 2021;46:63–115.

2. Liang F, Jiang X, Liu L, Wang F, Liu F, Hu S, Tan L, Chen X, Xu Y, Xu X, Jiang L, Liu Y, Yang C. White root rot of *Bletilla striata*: the pathogen, biological characterization, and fungicide screening. Front Microbiol. 2024;15.

3. Zhang M, Peng C, Li S, Tian C. Morphological and phylogenetic analyses reveal two new species of the *Fusarium fujikuroi* species complex in China. MycoKeys. 2025;112:127–163.

4. Sneha KB, Indra N, Murugavel K, Thangeswari S. Occurrence of *Gilbertella persicaria* causing soft rot of papaya in India. Physiol Mol Plant Pathol. 2024;134.

5. Ruangwong OU, Kunasakdakul K, Wonglom P, Dy KS, Sunpapao A. Morphological and molecular studies of a rare mucoralean species causing flower rot in *Hylocereus polyrhizus*. J Phytopathol. 2022;170(4):214–220.

6. Kapeua-Ndacnou M, Nóbrega TF, Batista LR, Evans HC, Abreu LM, Begoudé DAB, Reis TA, Barreto RW. *Aspergillus flavus* from coffee in Cameroon: a non-aflatoxigenic endophytic isolate antagonistic to coffee leaf rust (*Hemileia vastatrix*). J Appl Microbiol. 2023;134(5).

7. Wei S, Xu X, Wang L. Four new species of *Talaromyces* section *Talaromyces* discovered in China. Mycologia. 2021;113(2):492–508.

8. Qin J, Lyu A, Zhang QH, Yang L, Zhang J, Wu MD, Li GQ. Strain identification and metabolites isolation of *Aspergillus capensis* CanS-34A from *Brassica napus*. Mol Biol Rep. 2019;46(3):3451–3460.

9. Mazucato VS, Vieira PC. Exploring the chemical diversity of phytopathogenic fungi infecting edible fruits. Nat Prod Res. 2023;37(23):3947–3955.

10. Alfattani A, Marcourt L, Hofstetter V, Queiroz EF, Leoni S, Allard PM, Gindro K, Stien D, Perron K, Wolfender JL. Combination of pseudo-LC-NMR and HRMS/MS-based molecular networking for the rapid identification of antimicrobial metabolites from *Fusarium petroliphilum*. Front Mol Biosci. 2021;8.

11. Amalfitano C, Pengue R, Andolfi A, Vurro M, Zonno MC, Evidente A. HPLC analysis of fusaric acid, 9,10-dehydrofusaric acid and their methyl esters, toxic metabolites from weed pathogenic *Fusarium* species. Phytochem Anal. 2002;13(5):277–282.

12. Rohnert U, Heiser I, Nemec S, Baker R, Osswald W, Elstner EF. Diaphorase-mediated oxygen activation and uncoupling of mitochondrial electron transport by naphthazarin toxins produced by *Fusarium solani*. J Plant Physiol. 1998;153(5–6):684–692.

13. Shiono Y, Ariefta NR, Anwar C, Matsjeh S, Sappapan R, Murayama T, Koseki T, Kawamura T, Uesugi S, Kimura KI. New metabolites produced by *Fusarium solani* T-13 isolated from a dead branch. Phytochem Lett. 2016;17:232–237.

14. de Oliveira Almeida G, Mazucato VS, Tonani L, von Zeska Kress MR, Barbosa G, Krogh R, Andricopulo AD, Gomes Ferreira LL, Vieira PC. Exploring bioactive metabolites from *Fusarium falciforme* and *Aspergillus terreus* isolated from protease-rich fruits: antifungal, antitrypanosomal, and enzymatic inhibitory activities. Chem Biodivers. 2025;e00673.

15. Holenstein JE, Stoessl A. The marticins: confirmation of structure, elucidation of biosynthetic origin by 13C NMR studies, and revision of stereochemical assignments. Can J Chem. 1984;62(10):1971–1976.

16. Tang XX, Yan X, Fu WH, Yi LQ, Tang BW, Yu LB, Fang MJ, Wu Z, Qiu YK. New β-lactone with tea pathogenic fungus inhibitory effect from marine-derived fungus MCCC3A00957. J Agric Food Chem. 2019;67(10):2877–2885.

17. Chen C, Imamura N, Nishijima M, Adachi K, Sakai M, Sano H. Halymecins, new antimicrobial substances produced by fungi isolated from marine algae. J Antibiot. 1996;49(10):998–1005.

18. Liu SZ, Yan X, Tang XX, Lin JG, Qiu YK. New bis-alkenoic acid derivatives from a marine-derived fungus *Fusarium solani* H915. Mar Drugs. 2018;16(12):483.

19. Romans-Fuertes P, Sondergaard TE, Sandmann MIH, Wollenberg RD, Nielsen KF, Hansen FT, Giese H, Brodersen DE, Sørensen JL. Identification of the non-ribosomal peptide synthetase responsible for biosynthesis of the potential anti-cancer drug sansalvamide in *Fusarium solani*. Curr Genet. 2016;62(4):799–807.

20. Shao Y, Bai Y, Cai Z, Pu N, Zhang H. Optimization of stationary liquid fermentation conditions for N-methylsansalvamide production by the endophytic strain *Fusarium* sp. R1. Fermentation. 2024;10(3):140.

21. Wang YJ, Liu CY, Wang YL, Zhang FX, Lu YF, Dai SY, Li C, Sun Y, Pei YH. Cytotoxic cyclodepsipeptides and cyclopentane derivatives from a plant-associated fungus *Fusarium* sp. J Nat Prod. 2022;85(11):2592–2602.

22. Hemphill CFP, Sureechatchaiyan P, Kassack MU, Orfali RS, Lin W, Daletos G, Proksch P. OSMAC approach leads to new fusarielin metabolites from *Fusarium tricinctum*. J Antibiot. 2017;70(6):726–732.

23. Kakeya H, Kageyama SI, Nie L, Onose R, Okada G, Beppu T, Norbury CJ, Osada H. Lucilactaene, a new cell cycle inhibitor in p53-transfected cancer cells, produced by a *Fusarium* sp. J Antibiot. 2001;54(10):850–854.

24. Kleigrewe, K.; Aydin, F.; Hogrefe, K.; Piecuch, P.; Bergander, K.; Würthwein, E. U.; Humpf, H. U. Structure Elucidation of New Fusarins Revealing Insights in the Rearrangement Mechanisms of the *Fusarium* Mycotoxin Fusarin C. J. Agric. Food Chem. 2012, 60 (21), 5497–5505.

25. Maharjan, S.; Lee, S. B.; Kim, G. J.; Cho, S. J.; Nam, J. W.; Chin, J.; Choi, H. Isolation of Unstable Isomers of Lucilactaene and Evaluation of Anti-Inflammatory Activity of Secondary Metabolites Produced by the Endophytic Fungus *Fusarium* sp. QF001 from the Roots of *Scutellaria baicalensis*. Molecules 2020, 25 (4), 923.

26. Moni, F.; Saifullah, N.; Afroz, F.; Rony, S. R.; Sharmin, S.; Shahinuzzaman, A. D. A.; Al-Mansur, M. A.; Al-Reza, S. M.; Sohrab, M. H. Antibacterial and Cytotoxic Compounds from Endophyte *Fusarium solani* Isolated from *Centella asiatica* (L.). J. Biol. Act. Prod. Nat. 2022, 12 (5), 436–449.

27. Ibrahim, S. R. M.; Elkhayat, E. S.; Mohamed, G. A. A.; Fat’hi, S. M.; Ross, S. A. Fusarithioamide A, a New Antimicrobial and Cytotoxic Benzamide Derivative from the Endophytic Fungus *Fusarium chlamydosporium*. Biochem. Biophys. Res. Commun. 2016, 479 (2), 211–216.

28. Meng, L.; Tao, H.; Dong, G.; Yang, T.; Zhang, W.; Zhu, W.; Huang, C. ERK1/2 and Akt Pathway Activated during (3R,6R)-Bassiatin(1)-Induced Apoptosis in MCF-7 Cells. Cell Biol. Int. 2012, 36 (4), 345–348.

29. Malonek, S.; Bömke, C.; Bornberg-Bauer, E.; Rojas, M. C.; Hedden, P.; Hopkins, P.; Tudzynski, B. Distribution of Gibberellin Biosynthetic Genes and Gibberellin Production in the *Gibberella fujikuroi* Species Complex. Phytochemistry 2005, 66 (11), 1296–1311.

30. Zhang, M.; Chen, B.; Dai, H.; Sun, J.; Liu, H.; Han, J. Discovery of Antifungal Secondary Metabolites from an Intestinal Fungus *Fusarium* sp. J. Antibiot. 2023, 77 (3), 193–198.

31. Breinholt, J.; Ludvigsen, S.; Rassing, B. R.; Rosendahl, C. N.; Nielsen, S. E.; Olsen, C. E. Oxysporidinone: A Novel, Antifungal N-Methyl-4-Hydroxy-2-Pyridone from *Fusarium oxysporum*. J. Nat. Prod. 1997, 60 (1), 33–35.

32. Yang, M.; Ma, Y. Y.; Xiong, R. F.; Dong, M.; Hu, Q. F.; Li, Y. K. A New Amide from the Fermentation of *Fusarium* sp. SFS-G3 and Cytotoxic Activities. Chem. Nat. Compd. 2024, 60 (6), 1080–1083.

33. Xu, X.; Zhao, S.; Yu, Y.; Chen, Z.; Shen, H.; Zhou, L. Beauvericin K, a New Antifungal Beauvericin Analogue from a Marine-Derived *Fusarium* sp. Nat. Prod. Commun. 2016, 11 (12), 1825–1826.

34. Shi, S.; Li, Y.; Ming, Y.; Li, C.; Li, Z.; Chen, J.; Luo, M. Biological Activity and Chemical Composition of the Endophytic Fungus *Fusarium* sp. TP-G1 Obtained from the Root of *Dendrobium officinale* Kimura et Migo. Rec. Nat. Prod. 2018, 12 (6), 549–556.

35. Gu, M. J.; Han, S. E.; Hwang, K.; Mayer, E.; Reisinger, N.; Schatzmayr, D.; Park, B. C.; Han, S. H.; Yun, C. H. Hydrolyzed Fumonisin B1 Induces Less Inflammatory Responses than Fumonisin B1 in the Co-Culture Model of Porcine Intestinal Epithelial and Immune Cells. Toxicol. Lett. 2019, 305, 110–116.

36. Abbas, H. K.; Cartwright, R. D.; Xie, W.; Mirocha, C. J.; Richard, J. L.; Dvorak, T. J.; Sciumbato, G. L.; Shier, W. T. Mycotoxin Production by *Fusarium proliferatum* Isolates from Rice with Fusarium Sheath Rot Disease. Mycopathologia 1999, 147 (2), 97–104.

37. Govender, A. C.; Chuturgoon, A. A.; Ghazi, T. A Review on Fumonisin B1-Induced Mitochondrial Dysfunction and Its Impact on Mitophagy and DNA Methylation. Food Chem. Toxicol. 2025, 201, 115458.

38. Anumudu, C. K.; Ekwueme, C. T.; Uhegwu, C. C.; Ejileugha, C.; Augustine, J.; Okolo, C. A.; Onyeaka, H. A Review of the Mycotoxin Family of Fumonisins, Their Biosynthesis, Metabolism, Methods of Detection and Effects on Humans and Animals. Int. J. Mol. Sci. 2024, 26 (1), 184.

39. Groppi, E.; Haddad, M.; Cristofoli, V.; Vansteelandt, M.; Gadea, A. Unveiling the Substrate-Dependent Dynamics of Mycotoxin Production in *Fusarium verticillioides* Using an OSMAC-Metabolomics Approach. Chem. Biodivers. 2025, 22 (1), e202401747.

40. Hickert, S.; Cramer, B.; Letzel, M. C.; Humpf, H. U. Matrix-Assisted Laser Desorption/Ionization Time-of-Flight Mass Spectrometry Imaging of Ochratoxin A and Fumonisins in Mold-Infected Food. Rapid Commun. Mass Spectrom. 2016, 30 (23), 2508–2516.

41. Burgess, K. M. N.; Renaud, J. B.; McDowell, T.; Sumarah, M. W. Mechanistic Insight into the Biosynthesis and Detoxification of Fumonisin Mycotoxins. ACS Chem. Biol. 2016, 11 (9), 2618–2625.

42. Tamura, M.; Mochizuki, N.; Nagatomi, Y.; Harayama, K.; Toriba, A.; Hayakawa, K. Identification and Quantification of Fumonisin A1, A2, and A3 in Corn by High-Resolution Liquid Chromatography-Orbitrap Mass Spectrometry. Toxins 2015, 7 (2), 582–592.

43. Trisuwan, K.; Rukachaisirikul, V.; Borwornwiriyapan, K.; Phongpaichit, S.; Sakayaroj, J. Pyrone Derivatives from the Soil Fungus *Fusarium solani* PSU-RSPG37. Phytochem. Lett. 2013, 6 (3), 495–497.

44. Wen, H.; Li, Y.; Liu, X.; Ye, W.; Yao, X.; Che, Y. Fusagerins A–F, New Alkaloids from the Fungus *Fusarium* sp. Nat. Prod. Bioprospect. 2015, 5 (4), 195–203.

45. Burkhardt, I.; Siemon, T.; Henrot, M.; Studt, L.; Rösler, S.; Tudzynski, B.; Christmann, M.; Dickschat, J. S. Mechanistic Characterisation of Two Sesquiterpene Cyclases from the Plant Pathogenic Fungus *Fusarium fujikuroi*. Angew. Chem. Int. Ed. 2016, 55 (30), 8748–8751.
